# Reduced listening effort with adaptive binaural beamforming in realistic noisy environments

**DOI:** 10.1038/s41598-025-95045-3

**Published:** 2025-05-23

**Authors:** Joaquin T. Valderrama, Jorge Mejia, Angela Wong, Nicholas C. Herbert, Brent Edwards

**Affiliations:** 1 Department of Signal Theory, Telematics and Communications, University of Granada, 18014 Granada, Spain; 2 Research Centre for Information and Communications Technologies, University of Granada, 18014 Granada, Spain; 3 National Acoustic Laboratories, Sydney, NSW 2109 Australia; 4 Department of Linguistics, Macquarie University, Sydney, NSW 2109 Australia; 5 School of Computing, Macquarie University, Sydney, NSW 2109 Australia; 6 Research & Development, Sonova AG, 8712 Stäfa, Switzerland

**Keywords:** Listening effort, Directional microphones, Dual task, Reaction time, Alpha power, Ecological validity, Auditory system, Diagnostic markers

## Abstract

This study evaluates the effectiveness of adaptive binaural beamforming in a realistic cafeteria noise environment. The motivation stems from the common challenge faced by hearing aid users in such environments, where communication often demands significant mental effort. The study employed a combination of behavioural, neurophysiological, and self-reported measures to assess speech intelligibility and listening effort. Results showed that the adaptive binaural beamformer improved speech-in-noise intelligibility at signal-to-noise ratios (SNRs) yielding 80% and 95% intelligibility. Additionally, when this technology was enabled, listening effort was reduced across various metrics: faster reaction times on a dual task, decreased pre-stimulus alpha power (8–12 Hz), indicating less inhibition was needed, and increased alpha power during the encoding and retention phases, consistent with greater working memory load due to improved intelligibility. Self-reports indicated lower perceived effort in the more challenging SNR condition. The use of realistic background noise enhances the ecological validity of the findings, contributing to a better understanding of how this hearing aid technology performs in real-world listening environments. Overall, the study demonstrates that adaptive binaural beamforming can ease the cognitive burden on users in noisy, everyday environments, thereby enhancing their overall auditory experience.

## Introduction

Hearing aid users frequently report the substantial mental effort required to communicate in noisy environments^1,2^. Prolonged exertion of listening effort can lead to significant fatigue and may result in disengagement from conversations^3–5^. Regular exposure to these challenges increases the risk of developing anxiety due to fears of being misunderstood and may discourage social interaction^6,7^, leading to isolation^8^, which in turn can accelerate cognitive decline and dementia^9,10^. The hearing-aid industry has invested considerable effort in developing advanced technologies to facilitate communication in difficult acoustic environments^11,12^. The present study aims to assess the effectiveness of directional microphones in improving intelligibility and reducing listening effort within a realistic noisy scenario.

Directional microphones are designed to improve the signal-to-noise ratio (SNR) in loud acoustic scenarios with multiple sound sources, such as noisy cafeterias, restaurants and shopping centres^13^. Directional microphones enhance speech understanding by focusing on sounds coming from a specific direction, typically from in front of the user, while attenuating sounds from other directions. This is achieved through the use of two or more microphones that capture sound at different locations on the hearing aid. The device then processes the differences in timing and intensity between these signals to create a directional response, thereby helping users focus on conversations by minimising interference from surrounding noise sources^14^. Extensive literature demonstrates that directional microphones improve speech-in-noise intelligibility, particularly when the noise originates from directions other than the front of the listener^14–17^.

The Ease of Language Understanding (ELU) model is a theoretical framework that explains listening effort by describing how individuals process spoken language in various conditions^18–20^. According to the model, when auditory input matches the brain’s stored linguistic representations, understanding occurs effortlessly and automatically. However, when the input is degraded or unclear, such as in noisy environments or with hearing loss, the brain must dedicate additional cognitive resources, such as working memory, to reconstruct the message. This increased cognitive demand is perceived as listening effort. The ELU model advocates for a comprehensive assessment of hearing aid benefits, considering not only benefits in terms of speech-in-noise performance but also considering the amount of cognitive resources engaged in speech understanding^18–20^.

Listening effort can be measured using various methods, each providing unique insights into the cognitive demands of auditory processing^1^. *Behavioural* measures, such as the dual-task paradigm, assess listening effort by requiring participants to perform a primary listening task alongside a secondary task. The performance on the secondary task reflects the cognitive resources allocated to listening, with poorer performance indicating greater listening effort^21,22^. *Physiological* measures include techniques such as electroencephalography (EEG), pupillometry, and skin conductance. EEG is used to measure listening effort by analysing brain activity patterns, such as changes in neural oscillations, that reflect the cognitive load associated with processing auditory information in challenging acoustic environments^23–25^. Pupillometry, which tracks changes in pupil size, is another widely used measure, with larger pupil dilation indicating greater listening effort^24,26–28^. Skin conductance measures changes in sweat gland activity, providing an index of autonomic nervous system activation and is used to gauge stress and cognitive load during listening tasks^29,30^. *Self-reported* measures involve subjective ratings where individuals assess their own perceived listening effort, typically using scales or questionnaires. These self-assessments provide valuable insights into personal experiences of listening difficulty, complementing objective measures^31–33^.

Previous studies investigating the effects of directional microphones on listening effort have often examined them in addition to other signal processing algorithms aimed at selectively reducing noise components^34^. Recent literature presents mixed findings, highlighting the complexity of these technologies across different listening environments. Hornsby (2013) found no reduction in listening effort or mental fatigue when using directional microphones and noise reduction in hearing-impaired adults at SNRs producing 75% intelligibility in speech-shaped cafeteria babble^4^. Wu et al. (2014) examined hearing aid amplification and directional technology in two dual-task paradigms, and found that although speech recognition improved, listening effort did not decrease in older adults^35^. This contrasts with findings in younger populations, likely due to age-related declines in cognitive processing, making older adults less responsive to hearing aid technology in complex environments, where listening effort remains high despite improved speech recognition. Desjardins (2016) explored the individual and combined effects of noise reduction and directional microphones at SNRs producing 50% intelligibility, showing that listening effort was reduced with directional microphones alone or in combination with noise reduction, but not with noise reduction alone^16^. Bernarding et al. (2017) found that, relative to omnidirectional microphones, directional microphones and noise reduction enhanced intelligibility and reduced listening effort in 6-talker babble at SNRs producing 50% intelligibility, measured using self-reports and EEG biomarkers in the 7.68 Hz frequency band (alpha-theta border)^33^. Winneke et al. (2020) assessed wide versus narrow directional microphones in diffuse cafeteria noise and found that narrow directional microphones reduced both subjective listening effort and alpha power in neurophysiological measures^36^. These studies collectively suggest that while directional microphones and noise reduction can lower listening effort, their benefits are context-dependent and require further investigation in real-world listening environments.

This paper investigates the isolated effect of an adaptive binaural beamformer in a commercially available hearing aid on speech-in-noise intelligibility and listening effort. The study was conducted in a realistic Ambisonics cafeteria noise environment and used behavioural measures via a dual task, neurophysiological measures based on alpha power, and self-reported measures.

## Methods

### Ethics

The study was conducted at the National Acoustic Laboratories (NAL, Sydney, Australia) following methodologies in accordance with the Ethical Principles of the World Medical Association (Declaration of Helsinki) for medical research involving human subjects. The study protocols were approved by the Hearing Australia Human Research Ethics Committee (Ref. AHHREC2019-12). Informed consent was obtained from all subjects of the study.

### Participants

Potential candidates for the study were recruited from the NAL Research Participants Database (a registry of individuals who consented to be invited to participate in NAL research), and clients from Hearing Australia (a government-funded hearing service provider).

The study involved 20 participants (9 females, 19–81 years old, mean ± SD = 69.0 ± 18.8 years old), who met the five inclusion criteria: (1) age >18 years, (2) native English speakers, (3) absence of cognitive impairment, indicated by scoring above 85% on the Montreal Cognitive Assessment (MoCA)^37^, (4) more than 2 years of bilateral hearing aid use, and (5) bilateral downward-sloping hearing loss characterised by $\ge$30 dB hearing loss at 500 Hz, $\le$100 dB hearing loss at 3000 Hz, steepness $\le$20 dB/oct, and symmetry differences $\le$15 dB between the left and right 4-frequency average hearing loss (500, 1000, 2000, and 4000 Hz). Participants were compensated for their time at the conclusion of the study.

**Fig. 1:**
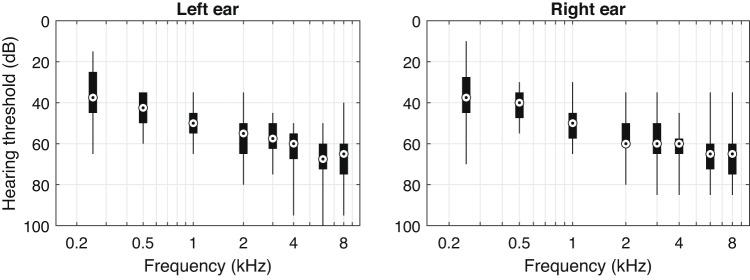
Pure-tone hearing threshold distributions from 250 to 8000 Hz. The central mark represents the median, the box edges are the 25th and 75th percentiles, and the whiskers are the maximum and minimum values.

Air-conduction pure-tone audiometry was conducted using an AC40 audiometer (Interacoustics A/S, Middelfart, Denmark). Figure 1 shows the quartile distributions of the participants’ pure-tone hearing thresholds at different frequencies ranging from 250 to 8000 Hz. Individual participant characteristics, including age, gender, first language, years of hearing aid use, MoCA score, and pure-tone audiometric thresholds at 250, 500, 1000, 2000, 3000, 4000, 6000, and 8000 Hz, are detailed in Section 1 of Appendix A in the online Supplementary Materials.

### Dual task paradigm

Listening effort was measured via a dual-task paradigm, in which participants performed two tasks simultaneously. The primary task involved repeating a sentence presented in background noise. This noise consisted of realistic cafeteria sounds obtained from the Ambisonics Recording of Typical Environments (ARTE) database^38^, presented at 65 dB sound pressure level (SPL) from an array of 41 speakers arranged spherically in five rows. Additionally, two distractors were positioned at $\pm 67^{\circ }$ azimuth to facilitate the evaluation of the adaptive binaural beamformer’s efficacy in suppressing nearby noise sources. These distractors were Australian female speakers delivering speech segments from real conversations, each presented at 65 dB SPL. Therefore, the total background noise level was approximately 70 dB SPL.

The target speech was the Australian version of the Matrix test^39^. This test uses a closed set of 10 words from the categories *Name* + *Verb* + *Quantity* + *Adjective* + *Object* to form sentences with identical syntax but unpredictable content (e.g., *Peter likes six red toys*). Words were 500 ms long with a 100 ms interval between them and were delivered from a speaker in front of the participant. The level was adjusted for each participant according to the SNR required to achieve 80% and 95% intelligibility in an aided condition, ensuring consistent speech reception thresholds (SRTs) across participants. The SRT-80 and SRT-95 were estimated in each participant from a psychometric function fitted to intelligibility scores over a range of SNRs from +15  to $-15$ dB. Detailed methodologies for estimating these SRTs are provided in Section 2 of Appendix A in the online Supplementary Materials. This appendix also shows the individual SRTs of each participant. The averaged SRT-80 and SRT-95 across participants were +0.1 dB and +4.6 dB, respectively.

**Fig. 2:**
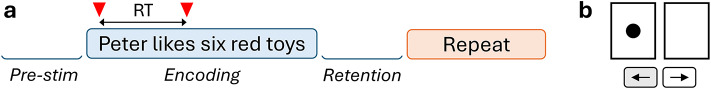
(**a**) Example of a trial in the dual-task paradigm. (**b**) A black circle is presented in the centre of the left vertical rectangle. Given that “Peter” is a male name, the correct response is to press the left arrow key (pointing *towards* the circle), which is highlighted with a grey background. Reaction time (RT) is measured from the auditory stimulus onset to the key press.

The secondary task involved a visual component triggered by the auditory stimulus of the primary task. Two large vertical rectangles were projected on an acoustically transparent screen in front of the participant. When the auditory stimulus began, a black circle appeared randomly in the centre of either the left or right rectangle. Participants were instructed to press the keyboard arrow key pointing *towards* the circle if the first word of the auditory stimulus was a *male* name, or the arrow key pointing *away* from the circle if it was a *female* name. Two seconds after the auditory stimulus ended (retention period), the black circle disappeared from the screen, and the participant repeated the words they had understood. Figure 2a illustrates the structure of an example trial. Figure 2b depicts a black circle in the centre of the left vertical rectangle. Given that the auditory stimulus in this example begins with a male name (Peter), the correct response is to press the left arrow key, highlighted with a grey background, as it points toward the circle.

Speech intelligibility was assessed by manually marking the correctly identified words. Participants’ listening effort was measured using three methods: *behaviourally*, through reaction time^21^ from sentence onset to key press; *neurophysiologically*, through alpha power^25,40^ recorded via a 64-channel SynAmps-RT NeuroScan electroencephalography (EEG) recording system (Compumedics Limited, Abbotsford, Australia) using a sampling rate of 1 kHz; and *self-reported*, with participants rating their perceived effort on a 7-point scale (i.e., no effort, very little, little, moderate, considerable, much, and extreme effort)^31^ after every five sentences. The dual-task paradigm was implemented in MATLAB (The Mathworks Inc., Natick, MA), using functions from the ‘Statistics and Machine Learning’, ‘Signal Processing’ and ‘Optimization’ toolboxes, along with the ‘Psychophysics Toolbox Version 3’ extension^41–43^.

### Preparatory & experimental sessions

The study comprised two preparatory sessions and one experimental session. In the first preparatory session, participants (i) received an overview of the study’s rationale and methods and signed a consent form; (ii) underwent a hearing assessment with otoscopy and air-conduction audiometry; (iii) completed the Montreal Cognitive Assessment (MoCA) to screen for cognitive impairment^37^; and (iv) had two sets of slim-tip earmold impressions taken: one vented appropriate for an acclimatisation period, and one with occluded vents for the experimental session.

Participants were scheduled for a second preparatory session after their earmolds were received at NAL facilities. This session included a practice session of the dual-task methodology, in which participants (i) read the test instructions (see Section 3 of Appendix A in the online Supplementary Materials), (ii) practised the test procedure by marking responses on a printed document (also available in the same appendix) without time constraints; and (iii) practised a simplified version of the dual-task test delivered on a laptop using headphones, first in quiet (only target speech presented), and then with background noise present. This structured approach ensured participants were well-prepared to perform the full dual-task test during the experimental session.

In the second preparatory session, participants were also fitted with Phonak Audéo M90-312 hearing aids (Sonova AG, Stafa, Switzerland) using Phonak Target 7.0.5 software and vented SlimTip earmolds. The hearing aids were adjusted to meet the NAL-NL2 target at 65 dB, as verified by real-ear measurement using Aurical FreeFit (Natus Medical Inc., Middleton, WI), and individual feedback tests were conducted. Participants were subsequently sent home with these devices for a 4-week acclimatisation period. During this acclimatisation period, the hearing aids defaulted to an automatic program that independently adjusted hearing aid settings based on the listening environment. To emulate a fitting with typical settings, all hearing aid features were left enabled at their default values. Frequency lowering was also permitted but could be disabled at the participant’s discretion. In addition to the automatic program, two manually selectable programs were also made available to the listener: (i) *Quasi-omnidirectional* (Q-Omni) and (ii) *Directional microphone* (DM). Both manual programs used the same settings as the default automatic program for speech in noise but differed in their microphone modes. Q-Omni employed a quasi-ominidirectional microphone strategy that simulates the ear’s natural directionality, whereas DM used an adaptive binaural microphone system, providing a highly directional listening beam. During the 4-weeks acclimatisation, participants were encouraged to try the two manual programs in loud acoustic scenarios to familiarize themselves with the sound. The two manual programs were selected for use in the experimental session. Compared to Q-Omni, acoustic measures showed that DM provided a +5.6 dB improvement in the articulation index-weighted directivity index^44^, and a +4.8 dB advantage in the articulation index SNR^44^ (details available in Section 4 of Appendix A in the Supplementary Materials online).

Participants attended the experimental session, which took place in the anechoic chamber of the Australian Hearing Hub (Sydney, Australia), after completing their acclimatization period. This session involved: (i) estimating the SNRs for 80% and 95% speech-in-noise intelligibility (SRT-80 and SRT-95) with hearing aids in Q-Omni mode, as detailed in Section 2 of Appendix A in the online Supplementary Materials; (ii) practising the dual-task test in quiet (without background noise); and (iii) conducting the dual-task test under four conditions–SRT-80 and SRT-95 in both the Q-Omni and DM hearing aid programs. Each condition was tested four times, resulting in a total of 16 blocks. Each block comprised 20 sentences, amounting to 80 sentences per condition. The order of conditions was pseudo-randomised by randomly varying the sequence of the four conditions. For example: [SRT-95 (Q-Omni) – SRT-80 (Q-Omni) – SRT-80 (DM) – SRT-95 (DM)] – [SRT-80 (DM) – SRT-95 (Q-Omni) – SRT-80 (Q-Omni) – SRT-95 (DM)] – [SRT-80 (Q-Omni) – SRT-95 (Q-Omni) – SRT-95 (DM) – SRT-80 (DM)] – [SRT-80 (DM) – SRT-95 (Q-Omni) – SRT-95 (DM) – SRT-80 (Q-Omni)]. This pseudo-randomisation aimed to balance the effects of learning across the test conditions. During the experimental session, hearing aids were fitted with occluded-vent earmolds to enhance the effectiveness of directional microphones^45^, and manual programs were used to select the desired hearing aid settings.

### Data analysis

#### Speech-in-noise intelligibility, reaction time, and self-reported effort

Data analysis was conducted in MATLAB using functions from the ‘Statistics and Machine Learning’ toolbox. The DM effect relative to Q-Omni was characterised in the two evaluated SRTs via a series of generalised linear mixed-effects (GLME)^46^ models. Speech-in-noise intelligibility, reaction time, or self-reported measures were considered as test variables; the hearing-aid program (Q-Omni or DM) was included as predictor variable; and participants were treated as a random effect. Lilliefors tests indicated that none of the test variables were normally distributed^47^, which justified the use of GLME models. GLME models also offer the advantage of accounting for repeated measures and provide robustness against missing and unbalanced data^48^. Additional GLME models were considered, incorporating the run order as a predictor variable to model any potential learning effects during the test.

Given that speech-in-noise intelligibility scores and self-reported measures consisted of nonnegative integers, the GLME models employed a Poisson distribution family with a natural logarithmic link function, i.e., $g(\cdot ) = \ln (\cdot )$, and an exponential inverse link function, i.e., $h(\cdot ) = g^{-1} = e^{(\cdot )}$^48^. Reaction times were deemed valid if the first word of the acoustic stimulus was correctly understood, thus excluding unreliable estimates of listening effort due to intelligibility issues. Considering the reaction time distributions across participants, reaction times below 400 ms were considered unreliable and were discarded from analysis. As reaction times consisted of positive numbers, a Gamma distribution family was used with $g(\cdot ) = (\cdot )^{-1}$ as link function, and $h(\cdot ) = g^{-1} = g(\cdot )^{-1}$ as inverse link function^48^.

Appendix B in the online Supplementary Materials provides the raw data for speech-in-noise intelligibility, reaction time, and self-reported measures of effort, along with custom MATLAB scripts for re-generating figures and performing the statistical analyses presented in the Results section.

#### Neurophysiological measures

Recorded EEG signals were processed using MATLAB, employing functions from the FieldTrip^49^ and EEGLAB^50^ toolboxes. Participant #P06 was excluded due to a technical issue with the triggers, resulting in a final sample size of 19 participants.

Participants’ EEG signals were processed in each test condition following the steps below: (i) data loading; (ii) visual identification of noisy channels; (iii) re-reference to the average across all channels, excluding the identified noisy channels, as well as the horizontal and vertical eye channels; (iv) interpolation of noisy channels by replacing them with the average of its neighbours, weighted by distance; (v) segmentation of data into 9-seconds trials; (vi) estimation of independent components via Independent Component Analysis^51^; (vii) visual identification of components related to eye-blinks and saccades; (viii) recomposition of data excluding eye-activity components; (ix) high-pass filtering of EEG signals with a 1 Hz cutoff frequency; (x) identification of noisy-trials based on absolute values exceeding $100~\upmu$V; (xi) interpolation of noisy trials by averaging neighbouring channels weighted by distance if fewer than 10 noisy channels were present, otherwise rejection of the entire trial; and (xii) power spectrum estimation through time-frequency analysis using a Morlet wavelet of 5 cycles within the 0–30 Hz range. Section 5 of Appendix A in the online Supplementary Materials provides the MATLAB script used for processing the EEG files from a selected participant, detailing each methodological step.

Differences in brain activity between Q-Omni and DM were investigated in the two evaluated SRTs across the time intervals [-1.0 – 0.0] s, [0.0 – 2.0] s, [2.0 – 3.5] s, and [3.5 – 5.0] s. These time intervals were chosen based on the averaged power spectra across participants and electrodes, which follow a related but distinct time structure to the time sections of the dual-task paradigm shown in Fig. 2. A cluster-based permutation test was used for statistical analysis to correct for multiple comparisons^52^. This test followed a two-stage process: (stage 1) calculation of a cluster-based test statistic, and (stage 2) determination of the significance probability. In stage 1, the procedure was as follows: (i) The alpha power difference (8–12 Hz) between the two hearing aid programs was characterised in each EEG channel using *t*-values; (ii) EEG channels with *t*-values exceeding the $97.5^{th}$ percentile (corresponding to a 0.05 significance threshold for a two-sided *t*-test) were identified; (iii) these selected channels were grouped into clusters; (iv) cluster-based statistics were computed by summing the *t*-values within each cluster; and (v) the largest cluster-level statistic was chosen as the cluster-based test statistic. In stage 2, the significance probability was calculated using a Monte Carlo method, as follows: (i) All trials were pooled into in a single dataset; (ii) a random partition was obtained by randomly selecting from the pooled dataset a number of trials equal to the size of the identified cluster in stage 1; (iii) the test statistic (i.e., the maximum cluster-level summed *t*-value) was computed for the random partition; (iv) steps (ii) and (iii) were repeated many times to generate a histogram of test statistics, using all possible number of randomisations to ensure optimal accuracy; and (v) the proportion of random partitions with a test statistic exceeding the cluster-based test statistic from stage 1 was calculated, yielding the Monte Carlo significance probability (*p*-value). Clusters were deemed statistically significant if their *p*-values were below the 0.05 threshold. Section 6 of Appendix A in the online Supplementary Materials provides the MATLAB script used for the statistical analysis.

## Results

### Speech-in-noise intelligibility

**Fig. 3:**
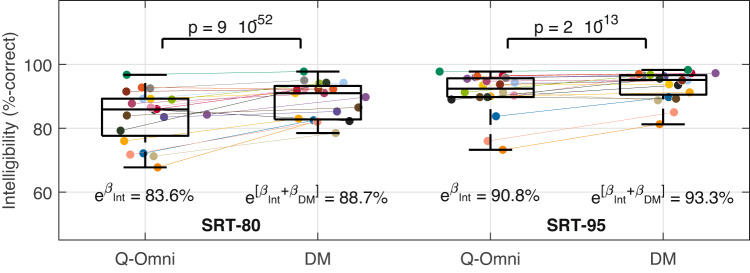
Mean intelligibility scores (%-correct) per participant are shown for the two speech reception threshold (SRT) conditions and the two hearing aid programs—Quasi-Omnidirectional (Q-Omni) and Directional Microphone (DM). Boxplots represent the quartiles of each distribution. Estimated scores and their associated *p*-values for each scenario are obtained from the generalised linear mixed-effects model in Table 1 (top-section). The colours assigned to each participant are detailed in Section 7 of Appendix A in the online Supplementary Materials. Int: Intercept.

Figure 3 shows the mean speech-in-noise intelligibility scores per participant across the four testing conditions. The GLME models presented in the top section of Table 1 indicate that at SRT-80, intelligibility improved from 83.6% with Q-Omni (estimated as $e^{\beta _{Intercept}}$) to 88.7% with DM ($p = 9\cdot 10^{-52}$, estimated as $e^{\beta _{Intercept}+\beta _{DM}}$); and at SRT-95, from 90.8% with Q-Omni to 93.3% with DM ($p = 2\cdot 10^{-13}$). Additionally, the GLME models in the bottom section of Table 1 demonstrated a statistically significant learning effect in both SRTs. At SRT-80, relative to the first run, intelligibility improved by 2.8% in the second run ($p = 2\cdot 10^{-10}$, estimated as $e^{\beta _{Intercept}+\beta _{Run2}} - e^{\beta _{Intercept}}$), 4.6% in the third run ($p = 2\cdot 10^{-24}$), and 5.6% in the fourth run ($p = 1\cdot 10^{-34}$). At SRT-95, compared to the first run, intelligibility improved by 1.8% in the third run ($p = 0.0001$), and 2.1% in the fourth run ($p = 9\cdot 10^{-6}$).

**Table 1:**
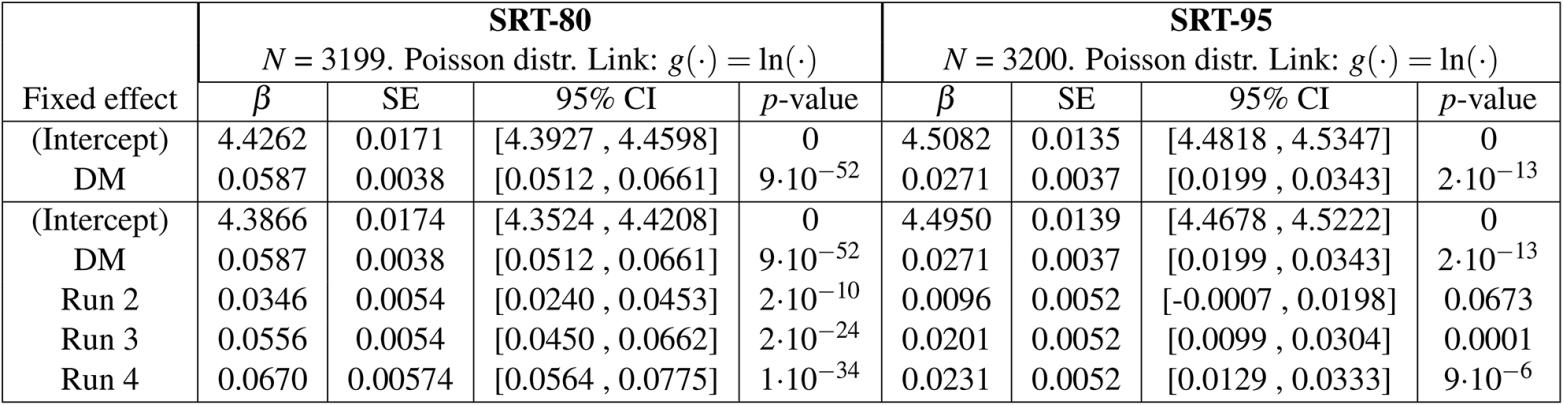
(Top section) Generalised linear mixed-effects (GLME) models for speech-in-noise intelligibility at the 80% and 95% speech reception threshold (SRT-80 and SRT-95), with the hearing aid program (Q-Omni: Quasi-Omnidirectional, DM: Directional Microphone) as a predictor variable and participants as a random effect. The intercept refers to the Q-Omni program. (Bottom section) Equivalent GLME models incorporating the run order as a predictor variable. The intercept corresponds to the Q-Omni program in the first run. GLME models used a Poisson distribution with a natural logarithmic link function. *N* number of observations, $\beta$ estimated value of the coefficient, *SE* standard error of the coefficient. *CI* confidence interval.

### Reaction time

**Fig. 4:**
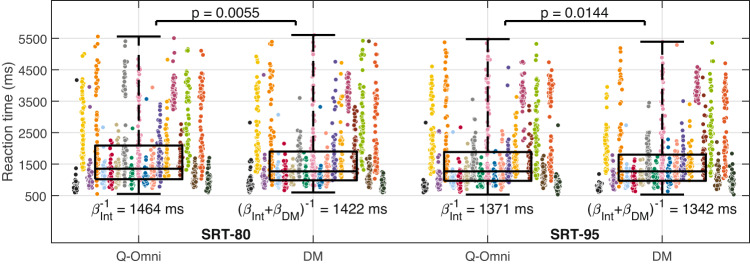
Individual reaction times (ms) per participant in the two speech reception threshold (SRT) conditions and the two hearing aid programs—Quasi-Omnidirectional (Q-Omni) and Directional Microphone (DM). Boxplots represent the quartiles of each distribution. Estimated scores and their associated *p*-values are obtained from the generalised linear mixed-effects model in Table 2 (top section). Participants #P01 to #P20 reaction time distributions are organised from left to right in each scenario. The colours assigned to each participant are detailed in Section 7 of Appendix A in the online Supplementary Materials. Int: Intercept.

Figure 4 shows the individuals’ reaction time data in the four testing conditions. Results from the GLME models presented in the top section of Table 2 show that the predicted reaction times for SRT-80 [Q-Omni], SRT-80 [DM], SRT-95 [Q-Omni] and SRT-95 [DM] were 1464 ms (estimated as $\beta _{Intercept}^{-1}$), 1422 ms (estimated as $[\beta _{Intercept} + \beta _{DM}]^{-1}$), 1371 ms, and 1342 ms, respectively. The DM effect over Q-Omni was found to be statistically significant, both at SRT-80 ($p = 0.0055$) and SRT-95 ($p = 0.0144$). The bottom section of Table 2 also shows a statistically significant learning effect in the two SRTs. At SRT-80, compared to the first run, reaction times were 255 ms shorter in the second run ($p=4\cdot 10^{-31}$), calculated as $(\beta _{Intercept}+\beta _{Run 2})^{-1} - \beta _{Intercept}^{-1}$, 309 ms shorter in the third run ($p=5\cdot 10^{-46}$), and 289 ms shorter in the fourth run ($p=7\cdot 10^{-39}$). At SRT-95, relative to the first run, reaction times were 143 ms shorter in the second run ($p=5\cdot 10^{-18}$), 208 ms shorter in the third run ($p=7\cdot 10^{-37}$), and 270 ms shorter in the fourth run ($p=6\cdot 10^{-59}$).

**Table 2:**
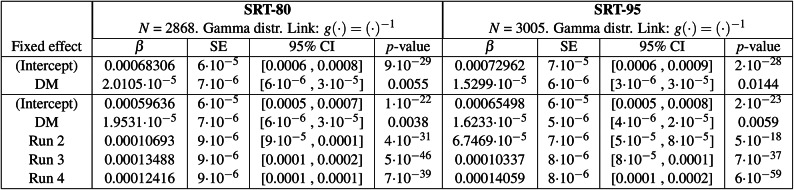
(Top section) Generalised linear mixed-effects (GLME) models for reaction time at the 80% and 95% speech reception threshold (SRT-80 and SRT-95), with the hearing aid program (Q-Omni: Quasi-Omnidirectional, DM: Directional Microphone) as a predictor variable and participants as a random effect. The intercept refers to the Q-Omni program. (Bottom section) Equivalent GLME models incorporating the run order as a predictor variable. The intercept corresponds to the Q-Omni program in the first run. GLME models used a Gamma distribution with an inverse function as link function. *N*: Number of observations. $\beta$: Estimated value of the coefficient. SE: Standard error of the coefficient. CI: Confidence interval.

### Self-reported effort

**Fig. 5:**
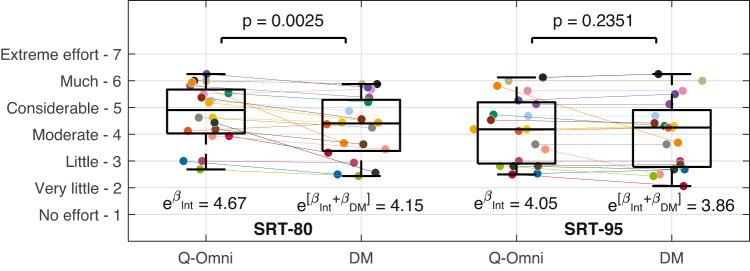
Mean self-reported effort scores of participants in the two speech reception threshold (SRT) conditions and hearing aid programs—Quasi-Omnidirectional (Q-Omni) and Directional Microphone (DM). Boxplots represent the quartiles of each distribution. Estimated scores and their associated *p*-values for each scenario are derived from the generalised linear mixed-effects model shown in Table 3 (top section). The colours assigned to each participant are detailed in Section 7 of Appendix A in the online Supplementary Materials. Int: Intercept.

Figure 5 shows the mean self-reported effort per participant for each test condition, along with the predicted scores and *p*-values resulting from the GLME model presented in the top section of Table 3. On a scale from 1 (*no effort*) to 7 (*extreme effort*), participants reported 0.52 units less effort with DM compared to Q-Omni at SRT-80 ($p=0.0025$). At SRT-95, DM resulted in 0.19 units less effort than Q-Omni, though this was not statistically significant ($p=0.2351$). No learning effect was observed in this measure.

**Table 3:**
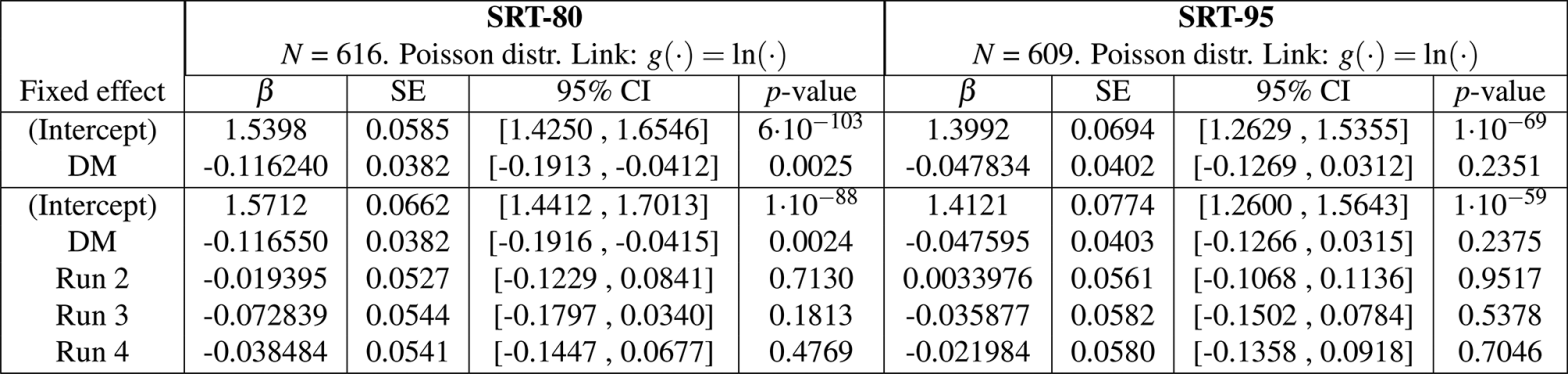
(Top section) Generalised linear mixed-effects (GLME) models for self-reported effort at the 80% and 95% speech reception threshold (SRT-80 and SRT-95), with the hearing aid program (Q-Omni: Quasi-Omnidirectional, DM: Directional Microphone) as a predictor variable and participants as a random effect. The intercept refers to the Q-Omni program. [Bottom section] Equivalent GLME models incorporating the run order as a predictor variable. The intercept corresponds to the Q-Omni program in the first run. GLME models used a Poisson distribution with a natural logarithmic link function. *N* number of observations, $\beta$ estimated value of the coefficient, *SE* standard error of the coefficient, *CI* confidence interval.

### Neurophysiological measures

**Fig. 6:**
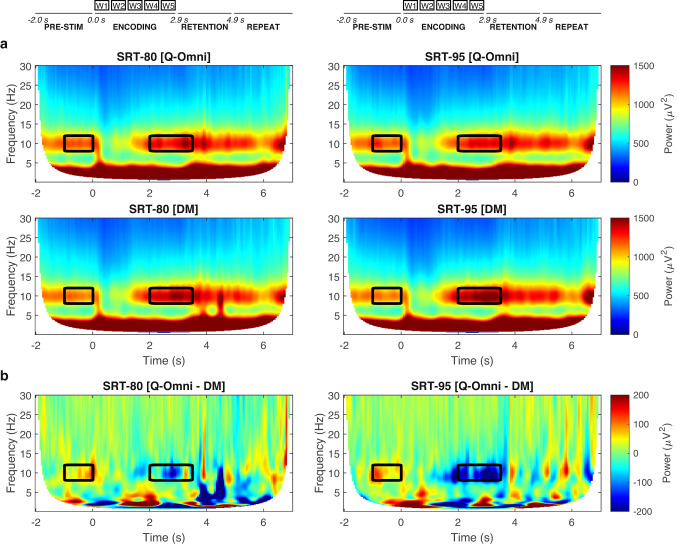
(**a**) Averaged power spectrum across participants and electrodes for the Quasi-Omnidirectional (Q-Omni) and Directional Microphone (DM) hearing aid programs in the two SRT conditions. A trial structure diagram is shown at the top. The black rectangles indicate the time invervals presenting statistically significant differences in alpha power (8–12 Hz) between Q-Omni and DM, i.e. [$-1.0$ – 0.0] s and [2.0 – 3.5] s. (**b**) Averaged power spectrum differences between the Q-Omni and DM programs across participants and electrodes for both SRTs.

Figure 6.a presents the averaged spectrogram across participants and electrodes for the Q-Omni (top) and DM (bottom) hearing aid programs under SRT-80 (left) and SRT-95 (right) conditions. A trial structure diagram is shown at the top. Brain activation patterns are consistent across all scenarios, showing prominent alpha power during the pre-stimulus, the final portion of encoding and in the retention periods. The black rectangles indicate the time intervals where Q-Omni and DM exhibited statistically significant differences in alpha power for each SRT. No significant differences were observed in the [0.0 – 2.0] s or [3.5 – 5.0] s intervals for any SRT condition. Individual spectrograms for each participant are provided in Section 8 of Appendix A in the online Supplementary Materials. These figures show consistent brain activation patterns across participants, and substantial individual variability in alpha power magnitude.

Figure 6.b shows the averaged power spectrum differences between the two hearing aid programs (Q-Omni – DM) across participants and electrodes for both SRTs. These plots show positive alpha power (greater in Q-Omni relative to DM) in the [$-1.0$ , 0.0] ms window, and negative alpha power (greater in DM relative to Q-Omni) in the [2.0 , 3.5] ms window.

**Fig. 7:**
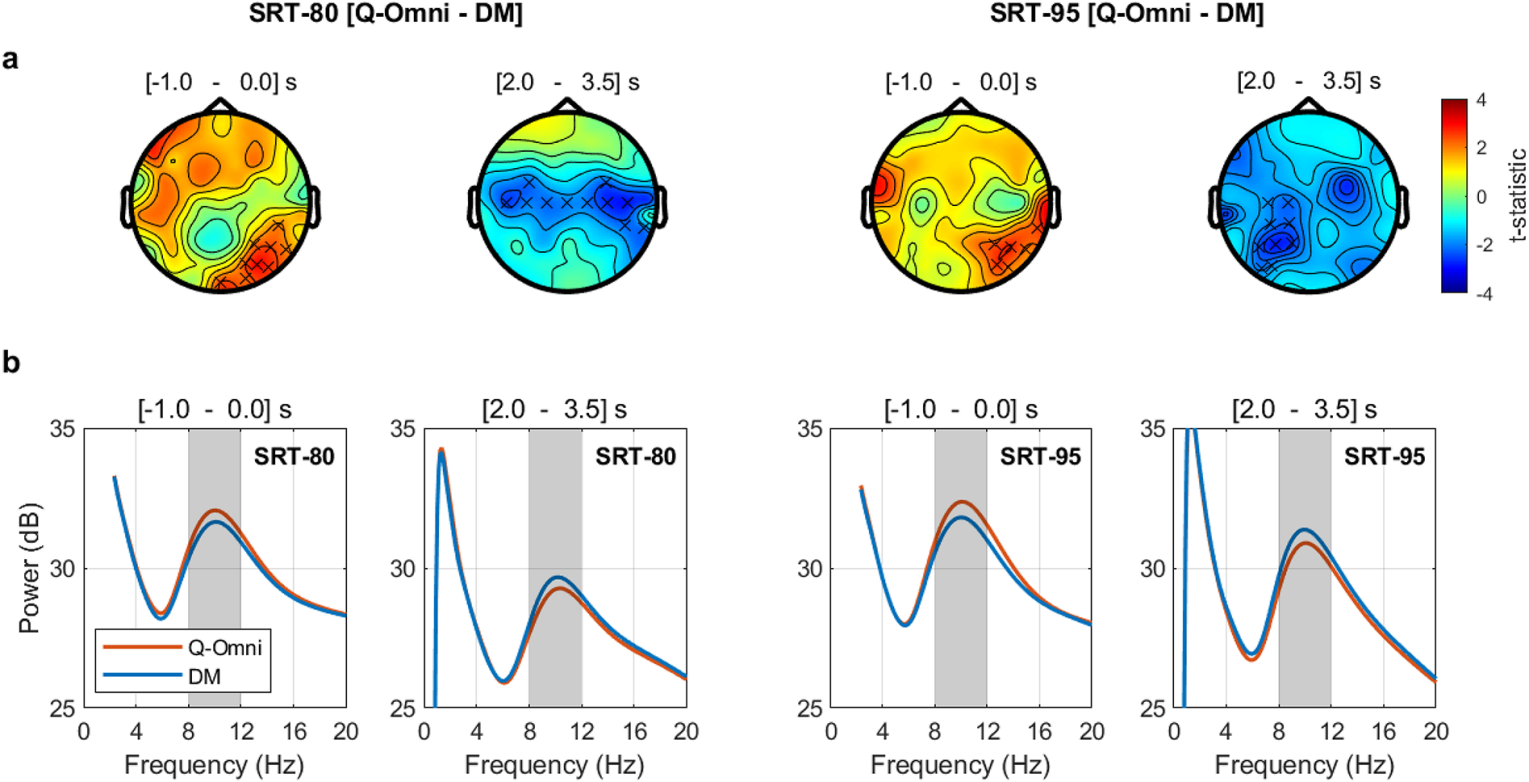
(**a**) Alpha power differences between Q-Omni and DM at SRT-80 (left) and SRT-95 (right) represented in terms of *t*-statistics. Crosses indicate EEG channels that form clusters with statistically significant absolute differences between the two hearing aid programs. (**b**) Averaged power spectrum across participants and EEG channels within each cluster for the two evaluated SRTs at the [$-1.0$ – 0.0] s and [2.0 – 3.5] s time intervals. The alpha frequency band is highlighted with a grey background.

Figure 7.a presents topographic maps of the *t*-statistic distribution across the scalp from the cluster-based permutation analysis on absolute alpha power differences between Q-Omni and DM in the [$-1.0$ – 0.0] s and [2.0 – 3.5] s intervals for SRT-80 and SRT-95. Crosses denote EEG channels within statistically-significant clusters. Results indicate a consistent effect of DM over Q-Omni in both SRTs. In the [$-1.0$ – 0.0] s interval, DM showed decreased alpha power (positive *t*-statistic values) in the right parietal-occipital region for both SRT-80 (cluster *t*-statistic = 20.98, $p=0.0106$) and SRT-95 (cluster *t*-statistic = 14.18, $p=0.0389$). In the [2.0 – 3.5] s interval, DM resulted in increased alpha power (negative *t*-statistic values) in centro-temporal areas for SRT-80 (cluster *t*-statistic = $-25.05$, $p=0.0219$) and in the left centro-parietal region for SRT-95 (cluster *t*-statistic = $-20.09$, $p=0.0272$).

Figure 7.b shows the averaged power spectrum across participants and EEG channels within each cluster for the [$-1.0$ – 0.0] s and [2.0 – 3.5] s intervals in both SRTs. Consistent with the topographic representations shown in Panel a, these plots visually show decreased alpha power with DM in the [$-1.0$ – 0.0] s interval and increased alpha power in the [2.0 – 3.5] interval across both SRTs.

## Discussion

This study aimed to investigate the effect of an adaptive binaural beamformer in a commercially available hearing aid on speech intelligibility in noise and listening effort. The research was conducted in a realistic Ambisonics-simulated cafeteria noise environment, utilising for the first time simultaneous measures of behavioural dual-task performance, neurophysiological alpha power monitoring, and self-reported data. By integrating these methods, we captured both objective and subjective aspects of auditory processing. Behavioural measures, such as dual-task performance, provided direct evidence of reduced cognitive load, while neurophysiological data (specifically alpha power) offered insights into the underlying brain activity associated with listening effort. Self-reports complemented these by capturing participants’ perceptions of effort. This multi-faceted approach allowed for a comprehensive evaluation of how hearing aids with directionality alleviate listening challenges in complex auditory environments.

Results show that adaptive binaural beamforming improves speech intelligibility in noisy environments. This aligns with previous research showing that directional microphones enhance speech perception by improving the SNR^15,26,35,36,53^. The binaural beamformer directs focus towards frontal speech signals while suppressing noise from other directions, thereby creating a more favourable listening environment. This directional focus enables hearing aid users to converse more effectively in noisy conditions, particularly in diffuse sound environments like a busy cafeteria.

The effect of directional microphones on listening effort were more complex. Behavioural dual-task performance revealed a reduction in listening effort, evidenced by faster reaction times when the hearing aid’s directionality was active. This reduction is consistent with the Ease of Language Understanding (ELU) model^18–20^, suggesting that a lower cognitive load allows for better performance in secondary tasks^21^. Neurophysiological measures further supported this finding, showing a reduction in pre-stimulus alpha power and an increase during the later portion of encoding when the beamformer was engaged. The reproducibility of this brain activation pattern across the two SRT conditions reinforces its reliability. The observed pattern likely reflects the dual roles of alpha-band oscillations: inhibition and information processing^25^. On one hand, pre-stimulus alpha, associated with inhibition^40^, was higher in the Q-Omni program, suggesting increased cognitive resource allocation to attenuate the effect of the louder background noise in that condition, thus indicating heightened listening effort in the Q-Omni program relative to DM. On the other hand, the increased alpha power during the later encoding phase in the DM condition likely reflects a greater working memory load due to retaining more words as a result of improved intelligibility^54–57^. While participants’ subjective ratings of listening effort were reduced in both SRT conditions, the effect of DM was minimal and statistically insignificant at SRT-95. The combination of behavioural, neurophysiological, and self-reported data supports the positive impact of these technologies on listening effort in challenging acoustic environments.

The findings of this study hold important clinical implications, particularly regarding how clinicians assess and manage listening effort. While self-reported measures are commonly used, this study highlights the potential for more objective measures, such as behavioural measures based on a dual-task, in revealing effort reductions that may not be consciously perceived. In this study, significant effects of directional microphones were observed in behavioural and neurophysiological measures at SRT-95, but these were not reflected in self-reports. Clinicians should be aware that improvements in listening effort due to directional microphones may occur even when patients do not report significant changes. It is therefore essential for audiologists to inform patients about the benefits of these technologies, even if the effects are not immediately obvious, in order to manage expectations and enhance satisfaction, ultimately improving clinical outcomes.

Interestingly, a statistically significant learning effect was observed in both speech intelligibility and reaction time measures over multiple dual-task runs, though not in self-reported listening effort. This discrepancy between objective performance improvements and subjective effort aligns with previous studies, suggesting that repeated exposure to difficult listening conditions can lead to perceptual learning, improving intelligibility and reaction times^58–60^. However, self-reported effort may remain unchanged, as these assessments are influenced more by cognitive load, emotional state, and individual differences rather than task mastery^1,61^. This highlights the complexity of measuring listening effort and the importance of using both objective and subjective assessments to provide a comprehensive evaluation, as subjective reports may not always reflect objective improvements^3^.

We also observed that the effects of DM were more pronounced at the lower SRT. This was reflected in greater improvements in speech-in-noise intelligibility (a 5.1% increase at SRT-80 compared to 2.5% at SRT-95), larger reductions in reaction times (42 ms at SRT-80 compared to 29 ms at SRT-95), greater self-reported benefits (a 0.52-point reduction on a 7-point scale at SRT-80 compared to 0.19 at SRT-95), and more distinct brain activation differences (cluster *t*-statistics of 20.98 and -25.05 during pre-stimulus and encoding at SRT-80, versus 14.18 and -20.09 at SRT-95). This pattern aligns with existing research, which shows that directional microphones and noise reduction systems are most effective in environments with lower SNRs^26,32,53,62^. These findings suggest that DM systems offer greater benefit in more challenging listening conditions, particularly where auditory processing demands are higher. By selecting SRTs of 80% and 95%, we also ensured that intelligibility was high enough for participants to engage meaningfully with the dual-task paradigm, minimising potential confounds related to motivation. Excessively low SNRs could lead to a lack of participant engagement, diminishing the perceived benefits of the hearing aid features^63,64^. Therefore, it is possible that at extremely low SNRs, the benefits of directionality may plateau, as the cognitive demand may surpass the technology’s capacity to assist.

A key strength of this study lies in the use of a realistic Ambisonics-simulated cafeteria noise environment at relatively high SNRs, which closely mimics the complex auditory landscapes that hearing aid users encounter in daily life. Previous studies have often relied on simplified laboratory settings that do not fully capture the spatial and temporal dynamics of real-world listening situations^16,32,53,62^. By simulating a realistic environment, this study provides insights that are more applicable to everyday listening conditions, thus enhancing the ecological validity of the findings. However, the effectiveness of hearing aid technologies may vary across different environments and user populations. While this study focused on cafeteria noise, further research should explore the generalisability of these findings to other challenging listening contexts, such as fluctuating outdoor noise or environments with significant reverberation. Methodological improvements to increase ecological validity could include using more realistic speech stimuli in the primary task, such as the Everyday Conversational Sentences in Noise (ECO-SiN) test^65^, which presents natural conversation in real-world background noise, and employing Ecological Momentary Assessment (EMA) methods to assess self-reported DM benefits in a broad range of everyday listening environments^66,67^.

## Conclusion

This study provides robust evidence that hearing aids incorporating adaptive binaural beamforming significantly enhance speech intelligibility and reduce listening effort in noisy environments, particularly under challenging conditions with lower signal-to-noise ratios. The combination of behavioural, neurophysiological, and self-reported data offers a comprehensive assessment of these benefits. Objective measures, such as faster reaction times and changes in alpha power, indicate a clear reduction in cognitive load when these hearing aid features are activated. However, subjective ratings of listening effort did not fully reflect the extent of these improvements, underscoring the need for both objective and subjective measures in assessing listening effort. Self-reports alone may not capture the nuanced reductions in cognitive load observed through behavioural and neurophysiological data. The use of a realistic Ambisonics-simulated cafeteria environment further strengthens the ecological validity of the findings, offering insights applicable to everyday listening challenges faced by hearing aid users. Clinically, this study highlights the value of directional microphones in improving listening outcomes, while emphasising that patient expectations should be carefully managed, given that subjective perceptions of benefit may not always align with measurable improvements in performance. Ultimately, these findings support the adoption of advanced hearing aid technologies to alleviate listening effort, particularly in complex auditory environments.

Portions of this research were presented at the (i) 5th International Conference on Cognitive Hearing Science for Communication(CHS-COM), Linköping, Sweden (2019)^68^, (ii) 45th Association for Research in Otolaryngology (ARO) Annual MidwinterMeeting, San Jose, CA (2022)^69^, and (iii) 7th International Conference on Cognitive Hearing Science for Communication(CHS-COM), Linköping, Sweden (2024)^70^.

## Supplementary Information

Supplementary Information.

## Data Availability

All raw data, processed data, programming scripts, and other research materials supporting the findings of this study are available upon reasonable request. Correspondence and requests for materials should be addressed to J.T.V.
