# Supplementary material for: Reduced listening effort with adaptive binaural beamforming in realistic noisy environments
Source: Sci Rep. 2025 May 23;15:17998. doi: 10.1038/s41598-025-95045-3 (PMC12102238; doi:10.1038/s41598-025-95045-3)
Supplement: Supplementary file 1 — Supplementary Information. [file 41598_2025_95045_MOESM1_ESM.zip › Appendix_A.pdf]

Reduced listening effort with adaptive binaural beamforming in realistic noisy environments

Supplementary material

Joaquin T. Valderrama[1,2,3,4,*], Jorge Mejia[4,5], Angela Wong[4], Nicholas C. Herbert[6], Brent Edwards[3,4]

[1] *University of Granada, Department of Signal Theory, Telematics and Communications, Granada, 18014, Spain*
[2] *University of Granada, Research Centre for Information and Communications Technologies, Granada, 18014, Spain*
[3] *Macquarie University, Department of Linguistics, Sydney, NSW 2109, Australia*
[4] *National Acoustic Laboratories, Sydney, NSW 2109, Australia*
[5] *Macquarie University, School of Computing, NSW 2109, Australia*
[6] *Research & Development, Sonova AG, Stäfa, 8712, Switzerland*
[*] *Corresponding author: jvalderrama@ugr.es*

Contents

1 Participants **2**

2 SNRs corresponding to 80% and 95% intelligibility **4**
2.1 Speech reception threshold (SRT) estimation 4
2.2 SRT results 5

3 Dual-task instructions **7**

4 Acoustic measures **9**
4.1 Directionality 9
4.1.1 Methods 9
4.1.2 Results 10
4.2 SNR advantage 10
4.2.1 Methods 10
4.2.2 Results 11

5 MATLAB script that processes the EEG files from a selected participant **12**

6 MATLAB script that performs a cluster-based permutational test on alpha power **19**

7 Assigned colours to each participant in Figures 3, 4 and 5 **22**

8 Individual spectrograms **23**

1 Participants

Table 1 presents the individual chacteristics of the participants, including their age, gender, first language, left and right 4-frequency average hearing loss (L-4FA, R-4FA, respectively, i.e.), years of hearing aid use, and their score on the Montreal Cognitive Assessment (MoCA)[Nasreddine et al., 2005] test. Table 2 presents the air-conduction pure-tone audiometric thresholds of each participant for frequencies ranging from 250 to 8000 Hz. These thresholds are also depicted in Figure 1.

ID	Gender	Age	First language	L-4FA	R-4FA	Years of hearing aid use	MoCA
P01	Male	26.06	English	53	53	22	87%
P02	Male	74.19	English	51	49	6	87%
P03	Male	75.68	English	52	55	7	97%
P04	Male	79.41	English	73	67	30	93%
P05	Male	33.98	English	54	56	8	90%
P06	Male	66.50	English	64	61	20	97%
P07	Female	79.86	English	44	49	10	93%
P08	Female	71.74	English	60	56	20	97%
P09	Male	75.67	English	40	38	10	93%
P10	Female	19.61	English	61	63	18	90%
P11	Male	81.35	English	49	56	12	93%
P12	Male	74.20	English	57	56	7	100%
P13	Female	77.42	English	52	51	7	100%
P14	Female	77.73	English	46	39	3	93%
P15	Male	80.15	English	52	49	6	77%
P16	Female	80.51	English	52	50	3	97%
P17	Female	81.26	English	47	50	20	80%
P18	Male	73.88	English	57	54	10	77%
P19	Female	75.60	English	55	48	5	100%
P20	Female	75.17	English	69	73	10	93%

Table 1: Individual characteristics of the participants. L- and R-4FA: left and right 4-frequency average hearing loss, respectively (500, 1000, 2000, 4000 Hz). MoCA: Montreal Cognitive Assessment.

ID	L-250	L-500	L-1000	L-2000	L-3000	L-4000	L-6000	L-8000	R-250	R-500	R-1000	R-2000	R-3000	R-4000	R-6000	R-8000	L-4FA	R-4FA
P01	35	35	50	60	60	60	55	40	35	35	50	60	60	60	50	40	53	53
P02	40	45	45	50	55	60	60	55	45	45	45	45	60	50	35	35	51	49
P03	20	40	55	50	50	65	60	60	20	35	50	60	65	65	70	65	52	55
P04	65	50	65	80	75	95	100	95	70	45	65	75	65	85	85	70	73	67
P05	30	35	50	65	60	60	55	50	30	35	50	65	65	65	60	50	54	56
P06	45	50	60	70	70	70	70	65	40	50	60	65	65	65	65	65	64	61
P07	25	40	40	45	45	50	60	60	35	40	45	55	45	60	60	65	44	49
P08	50	60	65	55	55	65	70	70	45	50	65	55	50	60	65	60	60	56
P09	25	35	35	35	45	50	65	70	25	35	30	40	35	50	60	75	40	38
P10	50	50	55	65	65	70	70	75	50	50	60	60	70	75	75	75	61	63
P11	45	45	50	50	50	50	50	65	45	45	55	60	60	60	60	75	49	56
P12	40	40	45	65	65	70	80	90	40	40	45	60	65	70	85	85	57	56
P13	45	50	45	50	60	55	80	60	35	45	45	55	55	55	75	60	52	51
P14	20	35	45	45	50	55	60	55	15	30	40	35	45	45	60	50	46	39
P15	35	50	55	45	50	60	60	75	40	50	45	35	50	65	70	75	52	49
P16	15	35	50	60	55	60	70	65	10	30	45	55	55	65	65	60	52	50
P17	15	35	45	55	45	55	70	90	10	35	50	60	50	55	70	85	47	50
P18	40	40	55	70	60	60	80	80	30	35	55	60	60	60	65	65	57	54
P19	55	50	50	50	60	65	75	70	45	40	40	45	55	60	70	80	55	48
P20	30	50	65	75	75	80	65	60	40	55	65	80	85	80	75	70	69	73

Table 2: Individual air-conduction pure-tone audiometric thresholds for frequencies ranging from 250 to 8000 Hz in the left (L) and right (R) ears.

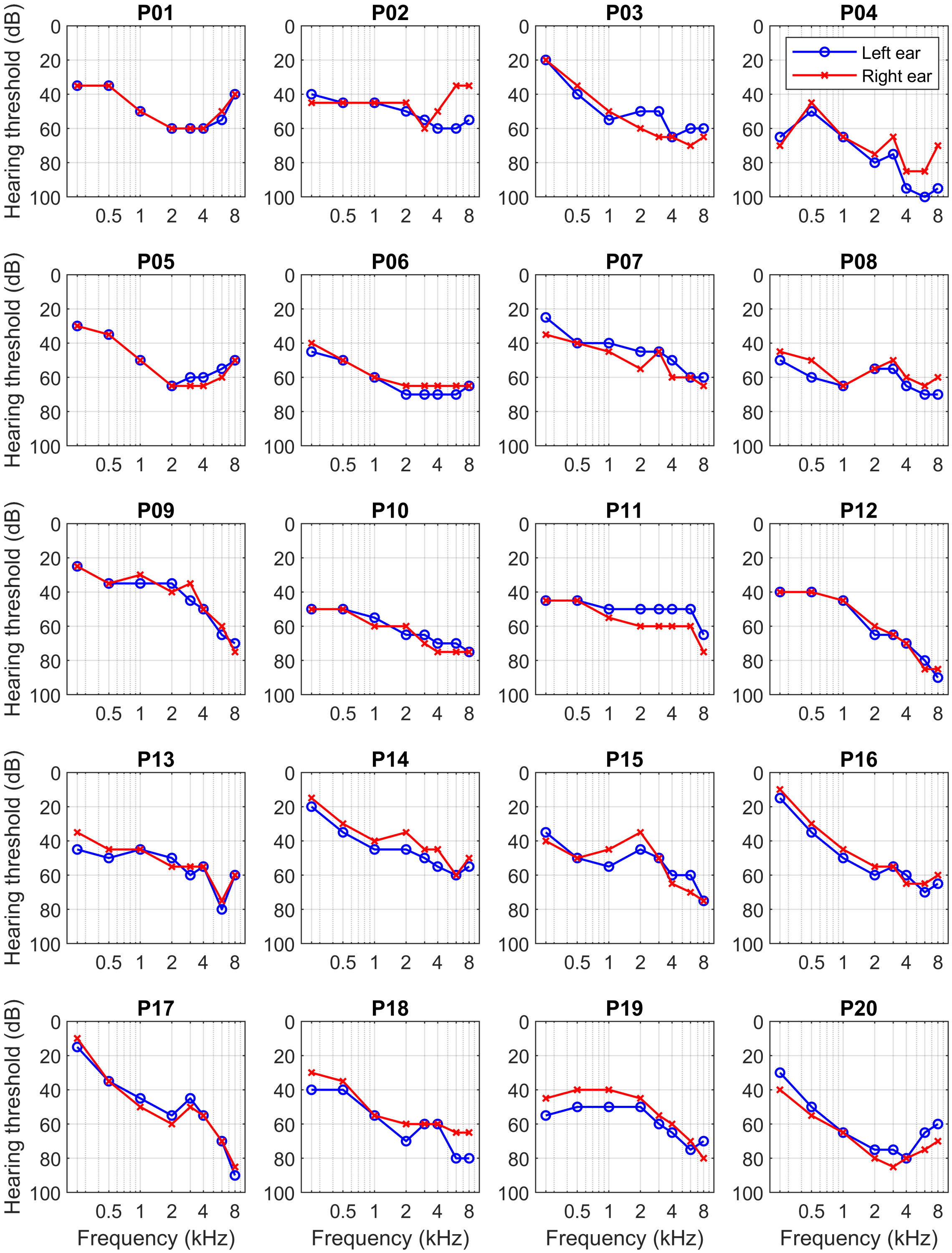

Figure 1: Individual air-conduction pure-tone audiometric thresholds for each participant.

2 SNRs corresponding to 80% and 95% intelligibility

2.1 Speech reception threshold (SRT) estimation

A custom-made MATLAB script (The Mathworks Inc., Natick, MA) was developed to estimate the signal-to-noise ratios (SNRs) required for correctly understanding 50%, 80%, and 95% of the words, known as speech-reception thresholds (SRT) SRT-50, SRT-80, and SRT-95, respectively. Participants were aided with hearing aids set up in Q-Omni program. Figure 2 displays the program's front-end, showing result estimates for participant #P01. The process involved (i) presenting sentences from the Australian version of the Matrix test[Kelly et al., 2017] with realistic background cafeteria noise and two additional distractors, and (ii) marking the correctly identified words. The background noise level was fixed at around 70 dB sound pressure level (SPL), while the target speech level varied to assess the percentage of correct words across a wide range of SNRs. Four sentences were presented at each SNR, ranging from +15 dB to -15 dB in 3 dB steps. Blue circles in Figure 2 represent the percentage of correctly identified words at each SNR. Since each sentences contains 5 words, percentages are reported in increments of 20%. At the end of the test, a sigmoidal curve is fitted to the data, and the SRT-50, SRT-80, and SRT-95 are estimated from this fitted curve.

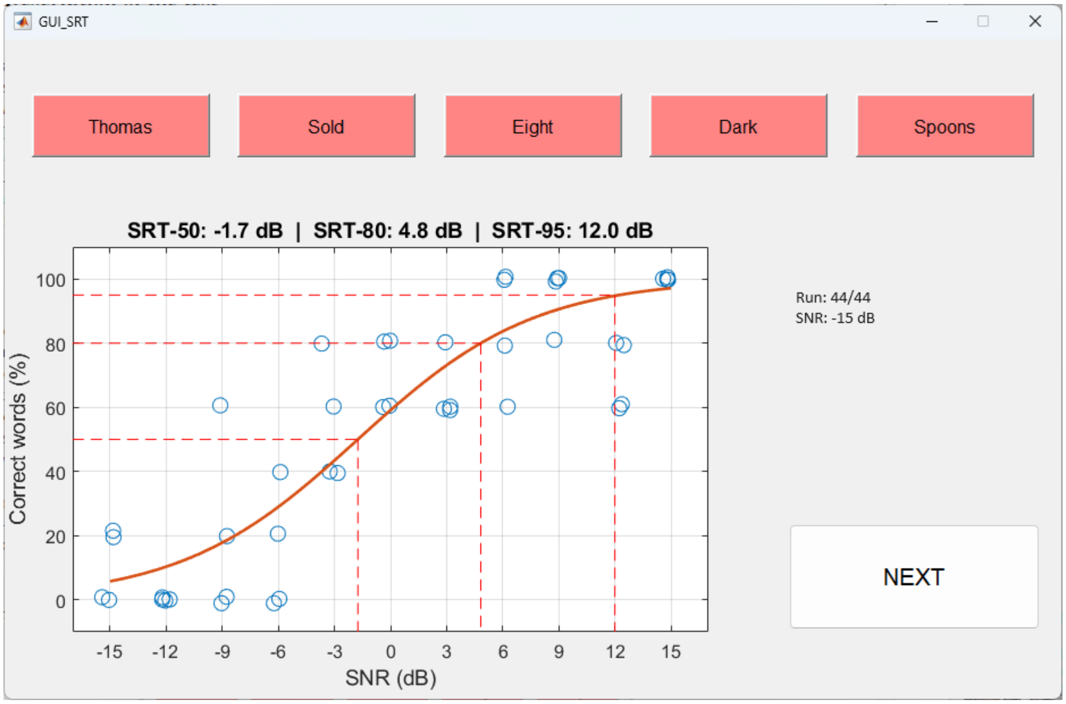

Figure 2: Front end of the custom-made MATLAB program designed to estimate the SRT-50, SRT-80, and SRT-95.

The specific **instructions to the participants** to conduct this part of the test are provided below.

In this test you will be asked to repeat back sentences presented in cafeteria noise. For example:<br><br>`Peter wants three old desks`<br><br>The level of the noise will always be the same but the level of the sentences will change. There are 48 sentences in total and your task is to repeat each sentence after you hear it. If you only hear part of the sentence, please just repeat what you have heard. If you are unsure about a particular word/s but think you know what is was you may take a guess.<br>The test is designed to be very challenging in places so please don't worry or feel frustrated if you do not understand all the words of a sentence. Just do the best you can.

2.2 SRT results

Table 3 shows the SNR (in dB) corresponding to each participant's SRT at 80% and 95% intelligibility (SRT-80 and SRT-95). For participants P04, P10, P12, and P19, who did not achieve 80% or 95% intelligibility, the SNR was set at +8 dB for SRT-80 and +12 dB for SRT-95. On average, the SRT-80 and SRT-95 across all participants were +0.1 dB and +4.6 dB, respectively. Figure 3 presents the fitted curves for intelligibility scores over an SNR range of -15 dB to +15 dB, along with individual SRT estimates for each participant.

	**P01**	**P02**	**P03**	**P04**	**P05**	**P06**	**P07**	**P08**	**P09**	**P10**
SRT-80	+4.8	-1.3	-5.6	+8.0	-3.2	-1.1	-5.6	+2.0	-2.2	+6.6
SRT-95	+12.0	+1.2	-2.2	+12.0	+0.8	+3.1	-2.8	+6.1	+3.5	+12.0
	**P11**	**P12**	**P13**	**P14**	**P15**	**P16**	**P17**	**P18**	**P19**	**P20**
SRT-80	-5.9	+8.0	-5.2	-3.6	+0.4	+1.7	-3.8	+2.6	+6.0	-0.7
SRT-95	-2.5	+12.0	-1.7	+0.8	+5.1	+7.4	+0.5	+8.1	+12.0	+3.5

Table 3: Signal-to-noise ratios (in dB) corresponding to each participant's 80% and 95% intelligibility (SRT-80 and SRT-95).

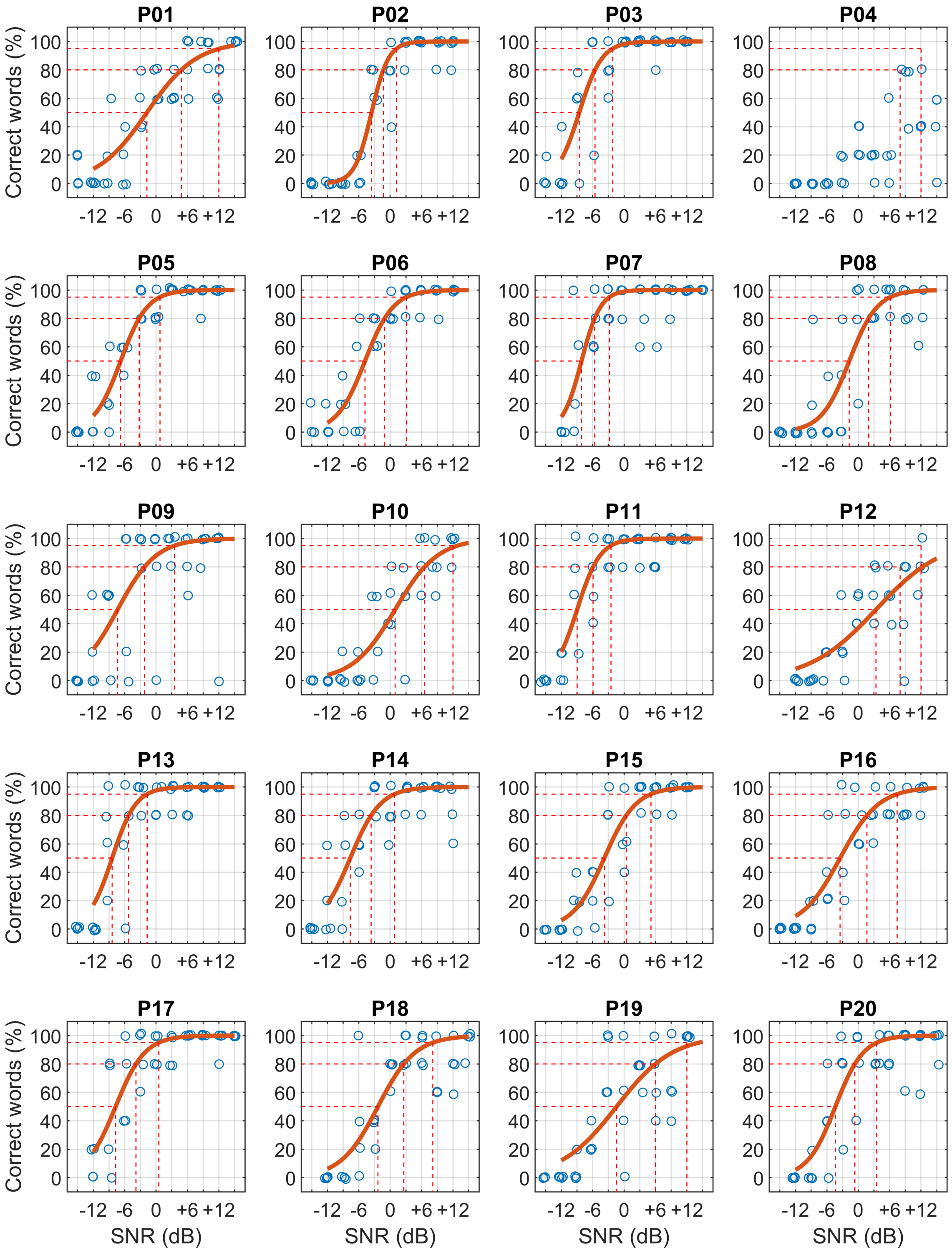

Figure 3: Intelligibility scores over a range of signal-to-noise ratios, and speech-reception threshold estimates for 80% and 95% intelligibility.

3 Dual-task instructions

Participants were asked to read the written instructions below on the dual-task methodology, and seek clarification if needed. Once they confirmed their understanding, they were given the document shown in Figure 4 to aid in learning and consolidating the test instructions. The first page of this document included a visual diagram and examples of the instructions. On the second page, participants were asked to mark the correct arrow key to press in 10 different scenarios.

INSTRUCTIONS TO PARTICIPANTS
This test measures how much **effort it takes to understand a sentence in noise**. You will be asked to do two tasks simultaneously:

1. The **first task** consists of repeating back a sentence of five words in the presence of realistic cafeteria noise. An example of the sentence is "Peter wants three old desks".
2. The **second task** is an audio-visual task. In this task, at the onset of the sentence a black circle will appear in any of the two vertical rectangles on the large screen in front of you. Your task is to press the left or right arrow in the keyboard according to the following rule:
    - If the name at the start of a sentence is *MALE*, press the arrow pointing *TOWARDS* the circle.
    - If the name of the person is from a *FEMALE*, press the arrow pointing *AWAY* the circle.

Please follow these guidelines:

- Look straight ahead to the center of the screen;
- use one hand, and leave your fingers just over the arrow buttons along the test so you don't have to look down;
- always use the same two fingers to press the two arrow buttons;
- press the button as fast as you can but not so fast that it stops from you being accurate;
- keep in your memory the words that you understood; and after the circle disappears, repeat back these words;
- when you feel ready for the next sentence, press any key to continue; and
- try not to feel frustrated if you don't understand most of the words in a sentence, the test is designed to be quite challenging in some parts.

The figure below illustrates an example corresponding to the example sentence "Peter wants three old desks". Since the name **Peter** is **male**, and the circle is in the right rectangle, the correct arrow to press is the **right arrow** (the arrow pointing towards the circle).

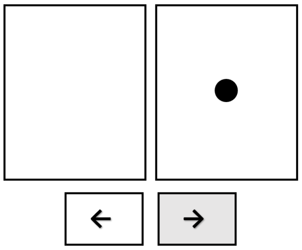

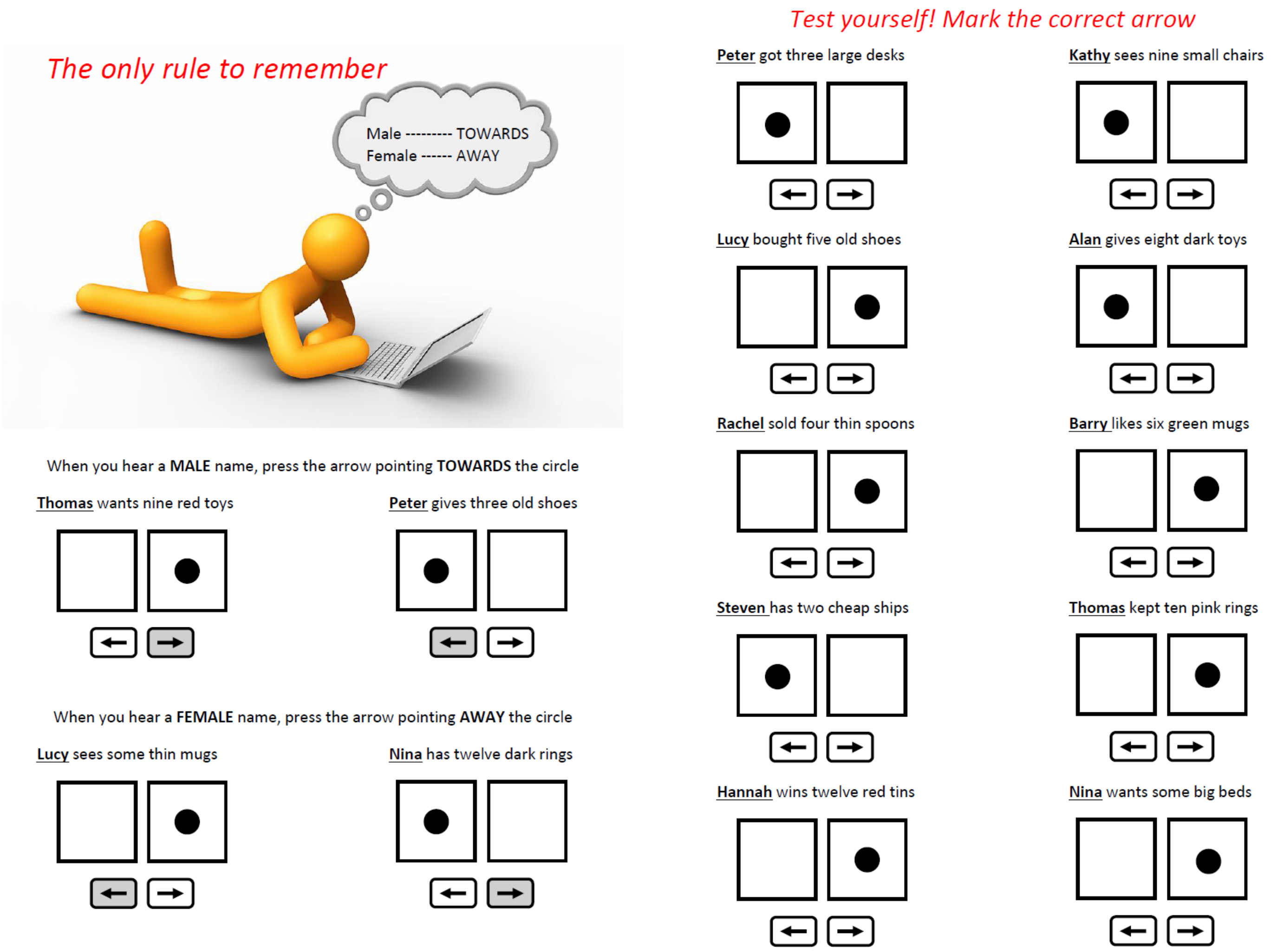

Figure 4: Document page used to learn and practice the dual-task instructions.

4 Acoustic measures

This section presents the experiments conducted to characterise the acoustic benefit provided by the *Directional Microphone* (DM) program–designed to enhance speech understanding in environments with high levels of background noise, incorporating an adaptive binaural microphone system providing a highly directional listening beam, relative to *Quasi-Omnidirectional* (Q-Omni)–a program designed to preserve the natural sound quality and spatial awareness using a quasi-ominidirectional microphone strategy that simulates the natural ear's directionality. These experiments consisted of (i) a directionality study, and (ii) an estimate of the SNR advantage. Results show that compared to Q-Omni, DM provides a benefit of +5.6 dB in the articulation index-weigthed directivity index [Killion et al., 1998] (section 4.1), and an articulation index SNR advantage of +4.8 dB [Killion et al., 1998] (section 4.2).

4.1 Directionality

4.1.1 Methods

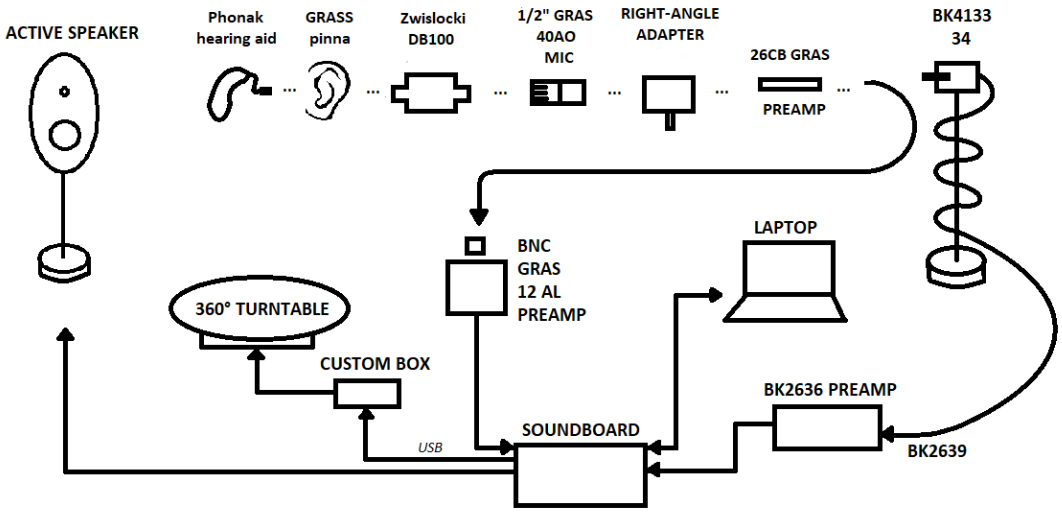

Figure 5: Hardware elements and connections.

Figure 5 shows a diagram with the hardware elements and connections. Phonak Audéo M90-312 hearing aids were fixed to a manikin for hearing aid testing (KEMAR 1974, GRAS Sound & Vibration A/S, Holte, Denmark). The earmolds were placed inside the pinna for KEMAR ([left ear] GRAS KB0066; [right ear] GRAS KB0065). KEMAR was fitted with a DB-100 2cc Zwislocki coupler, connected to a ½" GRAS 40AO microphone, which was connected to a 26CB GRAS preamplifier using a right-angle adapter, and then to a GRAS 12AL microphone preamplifier using a BNC cable. The output of the preamplifier was connected to a Multiface II soundcard (RME Audio, Haimhausen, Germany), which was connected to a laptop via an HDSP Cardbus card. KEMAR was placed on a chair, situated at the centre of a BK-3922 turntable, which was controlled by a MATLAB script (The Mathworks Inc., Natick, MA) and custom-made hardware. A speaker was placed at 2 meters from KEMAR, at the same height as the hearing aids.

Regarding the hearing-aid fitting, M receivers were used with an occluded cShell earpiece (due to the narrow canal size, SlimTip earmolds, as used in the main experiment, could not be employed). The NAL-NL2 [Keidser et al., 2012] fitting formula at 100% target gain with prescribed compression was used, applying the averaged air-conduction hearing thresholds across all participants of the study as input audiogram. The Q-Omni and DM programs were manually added and all features (besides microphone directionality) were disabled. The directional characteristics of the hearing aids in both programs were evaluated in linear mode, with the compression settings switched *off* [Kates, 2008].

The auditory stimulus consisted of 25 seconds of pink noise presented at 75 dB SPL. In all the measures, the first 20 seconds were allowed for activating the steering and noise reduction features of the hearing aid; and the remaining 5

seconds were used for analysis. The stimulus was presented firstly from a frontal noise source (0° azimuth), then at 15° azimuth increments for a full 360° rotation. Polar plots were constructed with a 15° precision by applying a $1/3^{rd}$ octave band filter-bank and then normalising each trace to the direction with maximum level. The plots used in this analysis had centre frequencies of 500 Hz, 2500 Hz, and 5000 Hz. The polar plots obtained from the left and right ears were averaged to form one single plot. Directionality was evaluated via (i) the *directivity index* (DI) – the ratio of the microphone output for sounds coming from 0° azimuth to the average of microphone output for sounds from all other directions in a diffuse field [Beranek, 1954]; and (ii) the *articulation index-weighted directivity index* (AI-DI) – the sum and average of the directivity index at each frequency multiplied by the articulation index weighting of the frequency band for speech intelligibility [Killion et al., 1998].

4.1.2 Results

Figure 6 shows the polar plots obtained at the three evaluated frequency bands. This figure shows that DM provides a higher degree of directionality, particularly in the 2500 Hz center frequency. Table 4 compares the directivity index of Q-Omni and DM at the three evaluated center frequencies. Considering the full frequency spectrum, the difference in AI-DI between DM and Q-Omni was +5.6 dB (i.e. AI-DI was equal to -1.8 dB in Q-Omni and +3.8 dB in DM).

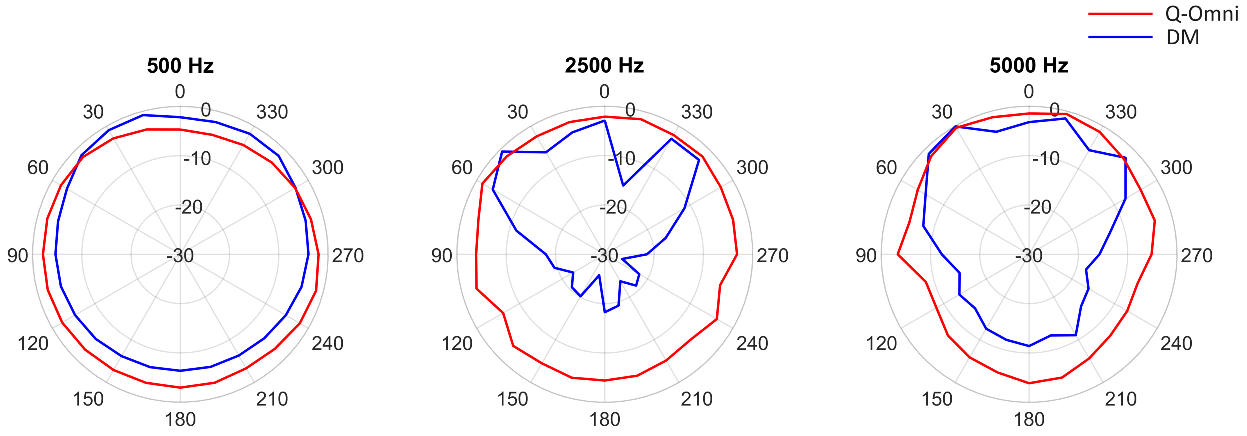

Figure 6: Polar plots at different $1/3^{rd}$ octave frequencies in 'Quasi-Omnidirectional' (Q-Omni) and 'Directional Microphone' (DM) programs.

Center frequency	Q-Omni	DM
500 Hz	-1.81 dB	+0.34 dB
2500 Hz	+3.81 dB	+5.27 dB
5000 Hz	+0.46 dB	+3.02 dB

Table 4: Directivity Index of 'Quasi-Omnidirectional' (Q-Omni) and 'Directional Microphone' (DM) programs at 500 Hz, 2500 Hz and 5000 Hz.

4.2 SNR advantage

4.2.1 Methods

The purpose of this experiment was to the measure the objective SNR advantage provided by DM relative to Q-Omni at different frequencies. The hardware setup was similar to the one presented in Figure 5. KEMAR hearing aids were fitted as described in section 4.1.1, but this time with compression-*on*.

The acoustic scenario was similar to the material used in the dual-task, described in the 'Dual task paradigm' section of the main paper. The auditory stimulus consisted of 50 concatenated words of 0.5 seconds corresponding to the Australian version of the Matrix test [Kelly et al., 2017], presented from the front speaker of the 41-channel Ambisonics array of speakers at 68 dB sound pressure level (SPL). Noise consisted of realistic cafeteria noise from the Ambisonics Recordings of Typical Environments (ARTE) database [Weisser et al., 2019] and two added distractors at ±67° presented at 65 dB SPL (with a combined noise level around 70 dB SPL). The total level of target + noise was around 72 dB SPL.

The SNR benefit between different conditions was estimated according to the methodology described by Hagerman and Olofsson [2004]. This methodology consisted in dividing the auditory stimulus into segments of 6 seconds with some degree of overlapping, and presenting these segments in the following polarities with respect the polarity of the noise: (A) [- -] (i.e., stimulus in negative polarity and noise in negative polarity); (B) [+ -] (i.e., stimulus in positive polarity and noise in negative polarity); and (C) [+ +]. This way: A+B ends up with just the noise; A-B ends up with just the stimulus; B+C ends up with just the stimulus; and B-C ends up with just the noise. The error can be estimated by comparing the two corresponding calculations, i.e., [A+B vs B-C] for the noise and [A-B vs B+C] for the stimulus. The first 20 seconds of auditory stimulation were discarded from the analysis in order to allow for adaptation of the hearing aids. The overall benefit was evaluated via the articulation index SNR benefit [Killion et al., 1998].

4.2.2 Results

Figure 7 shows the SNR advantage of DM relative to Q-Omni at different frequencies. The articulation index – SNR benefit of DM over Q-Omni was estimated as +4.6 dB.

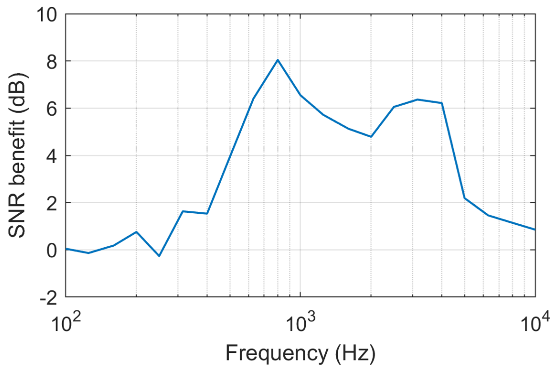

Figure 7: SNR benefit (dB) of 'Directional Microphone' (DM) relative to 'Quasi-Omnidirectional' (Q-Omni) at different frequencies.

5 MATLAB script that processes the EEG files from a selected participant

```
%% EEG processing of a selected participant
clear,clc
addpath('FieldTrip')                  % Add the FieldTrip directory

%% Variables initialisation
Participant = 1;                      % Selected participant
% TestCond: 1.SRT80-QOmni;  2.SRT80-DM;  3.SRT95-QOmni;  4.SRT95-DM
A = load('TestCond');                 % Test condition order per participant
TestCond = A.TestCond;      SBJ = A.SBJ;        clear A
NTests = 16;                          % Number of tests per participant
NSentences = 20;                      % Sentences per file
Thr_Value = 100;                      % Threshold for trial rejection in uV
Thr_Range = 500:5000;                 % Time range for artifact rejection
L_Window = 9;                         % Length in seconds of the time window
FMax = 30;                            % Maximum evaluated frequency
DEBUG = 0;                            % Flag to view EEG data

%% Select Noisy channels and ICA components to reject
switch Participant
    case 1
        NoisyChans = {'TP7','TP8','FT8','T8'};
        ICA_ComponentsReject = [12,20,46];
    case 2
        NoisyChans = {'T7','PO7','FPZ','FP2'};
        ICA_ComponentsReject = [1,22,47,59,60];
    case 3
        NoisyChans = {};
        ICA_ComponentsReject = 1;
    case 4
        NoisyChans = {'FT8'};
        ICA_ComponentsReject = [1,3,52,54,58];
    case 5
        NoisyChans = {'F3','T7','T8','CP2'};
        ICA_ComponentsReject = [2,3,4];
    case 7
        NoisyChans = {'T7','T8','TP7','F7','O1'};
        ICA_ComponentsReject = [1,2,3,23,31,37,51,52];
    case 8
        NoisyChans = {'F6','P7','TP7'};
        ICA_ComponentsReject = [2,3,4,5,23];
    case 9
        NoisyChans = {'F5','FC5','C6','C5','T8','T7'};
        ICA_ComponentsReject = [1,2,12,40,58];
    case 10
        NoisyChans = {'F6','FC2','FT7','T8','F8'};
        ICA_ComponentsReject = [1,2,3,27,38,50,54,59];
    case 11
        NoisyChans = {'C6','T7','T8','HEO','TP7','FP1'};
```

```
        ICA_ComponentsReject = [1,2,23,34,36,44,48,51];
    case 12
        NoisyChans = {'FP1','F8','T7','O2','FT8','T8'};
        ICA_ComponentsReject = [1,2,16,50];
    case 13
        NoisyChans = {'FPZ','FZ','FC1','C5','C3','CP6','P2','PO4','F6',...
            'O1','TP8'};
        ICA_ComponentsReject = [1,2,5,13,23,36,44,50];
    case 14
        NoisyChans = {'F4','P8','M2','T8','PO5'};
        ICA_ComponentsReject = [1,2,3,6,10,19,23,30,42,50];
    case 15
        NoisyChans = {'FC2','FC5','F8'};
        ICA_ComponentsReject = [1,2,6,8,12,13,15,23,37,41];
    case 16
        NoisyChans = {'F6','F8','FT7','FC5','FC6','FT8','O1'};
        ICA_ComponentsReject = [1,4,6,10,13,16,25,30,35,42,44,51,52];
    case 17
        NoisyChans = {'FPZ','FC6','C5','CP5'};
        ICA_ComponentsReject = [1,2,3,6,7,8,11,13,14,15,18,19,23,25,28,...
            29,30,31,35,36,39,41,42,44,49,50,54,56,58,59];
    case 18
        NoisyChans = {'T8'};
        ICA_ComponentsReject = [1,32,43,48,61];
    case 19
        NoisyChans = {'T7','TP8','TP7'};
        ICA_ComponentsReject = [1,2,3,4,5,24,31,36,45,47,59];
    case 20
        NoisyChans = {'T8','FC6','P7','TP7','TP8','T8','O2','OZ','M2'};
        ICA_ComponentsReject = [1,2,42,44,50,51];
end

%% Segment all files from one participant
% Section initialisation
Segmented_Data = cell(NTests,1);
cfg = [];
cfg.method = 'triangulation';
cfg.layout = 'EEG1010.lay';
neigh = ft_prepare_neighbours(cfg); % Prepare neighbour channels

for test=1:NTests
    % LOAD data
    FileName = ['Database\P0' SBJ(Participant,:) '\test' Idx2Str(test) ...
        '.cnt'];
    cfg = [];
    cfg.dataset = FileName;
    cfg.channel = {'all','-CB1','-CB2','-EKG','-EMG'};
    data = ft_preprocessing(cfg);
    fs = data.fsample;

```

```
    % VIEW data and identify noisy channels by visual inspection
    if DEBUG
        cfg = [];
        cfg.viewmode = 'vertical';
        cfg.ylim = [-20 20];
        cfg.blocksize = 9;
        cfg.preproc.hpfilter = 'yes';
        cfg.preproc.hpfreq = 1;
        cfg.channel = 1:64;
        ft_databrowser(cfg,data)
    end

    % RE-REFERENCE excluding noisy channels
    cfg = [];
    cfg.reref = 'yes';
    cfg.refchannel = {'all','-VEO','-HEO'};
    for k=1:length(NoisyChans)
        cfg.refchannel{k+3} = join(['-' NoisyChans{k}]);
    end
    data = ft_preprocessing(cfg,data);

    % INTERPOLATION
    cfg = [];
    cfg.method = 'nearest';
    cfg.layout = 'EEG1010.lay';
    cfg.neighbours = neigh;
    for k=1:length(NoisyChans)
        cfg.badchannel = NoisyChans(k);
        data = ft_channelrepair(cfg,data);
    end

    % SEGMENTATION
    % Read triggers
    Triggers = ft_read_event(FileName);
    Triggers = ft_filter_event(Triggers,'type','trigger','value',20);
    Triggers = Triggers(1:NSentences);

    % Segmentation features
    Triggers = [Triggers.sample]';
    Prestim = 2.0*fs;        Poststim = (L_Window-2.0)*fs;
    % [1column: start    2column: end      3column: eventstart or offset]
    Triggers = [Triggers-Prestim Triggers+Poststim ...
        -Prestim*ones(length(Triggers),1)];

    % Segment data
    cfg = [];
    cfg.trl = floor(Triggers);
    Segmented_Data{test} = ft_redefinetrial(cfg,data);
end

```

```
%% Remove eye blinks using ICA
% Combine data from all files - this speeds up ICA
data = Segmented_Data{1};
SampleInfo = [data.sampleinfo ; nan(300,2)];
for k=2:NTests
    data = ft_appenddata([],data,Segmented_Data{k});
    SampleInfo((k-1)*NSentences+1:k*NSentences,:) = ...
        Segmented_Data{k}.sampleinfo;
end
data.sampleinfo = SampleInfo;
clear SampleInfo

% Triggers adjustment to avoid error in later stages
for k=1:NTests
    data.sampleinfo((k-1)*NSentences+1:k*NSentences,:) = ...
        data.sampleinfo((k-1)*NSentences+1:k*NSentences,:)+(k-1)*1000000;
end

% Separate independent components using ICA
cfg = [];
cfg.channel = {'all'};
cfg.randomseed = 1;                          % Set to 1 for replicability
cfg.numcomponent = 64-length(NoisyChans);    % Number of healthy channels
cfg.method = 'runica';
data = ft_componentanalysis(cfg,data);       % Takes time [around 15 mins]

% Identify noisy components
if DEBUG
    set(0,'DefaultFigureColormap',feval('jet'));                          %#ok
    cfg = [];
    cfg.layout = 'EEG1010.lay';
    cfg.viewmode = 'component';
    ft_databrowser(cfg,data);
end

% Recompose data excluding components identified as blinks and eye movement
cfg = [];
cfg.component = ICA_ComponentsReject;
data = ft_rejectcomponent(cfg,data);

%% Trials rejection
% High-pass filter the data [1 Hz]
cfg = [];
cfg.hpfilter = 'yes';
cfg.hpfreq = 1;
data = ft_preprocessing(cfg,data);

% Section initialisation
AcceptedTrials = [];                     % Accepted trials initialisation
cfg = [];
```

```
cfg.method = 'nearest';
cfg.layout = 'EEG1010.lay';
cfg.neighbours = neigh;

% Interpolate noisy trials if there are <10 noisy trials
for k=1:NTests*NSentences
    Noisychans = 0;
    for chan=1:62
        if max(abs(data.trial{k}(chan,Thr_Range)))>Thr_Value
            % Interpolate noisy sweeps from neighbour channels
            cfg.trials = k;
            cfg.badchannel = data.label(chan);
            tmp = ft_channelrepair(cfg,data);
            data.trial{k} = tmp.trial{1};
            Noisychans = Noisychans+1;
            clear tmp
        end
    end
    if Noisychans<10, AcceptedTrials = [AcceptedTrials k]; end          %#ok
    clear Noisychans
end
data.acc = AcceptedTrials;
clear AcceptedTrials

%% Time-Frequency analysis
TrialsSRT80_QOmni = nan(1,NTests*NSentences);   % SRT80-QOmni
TrialsSRT80_DM = nan(1,NTests*NSentences);      % SRT80-DM
TrialsSRT95_QOmni = nan(1,NTests*NSentences);   % SRT95-QOmni
TrialsSRT95_DM = nan(1,NTests*NSentences);      % SRT95-DM
for test=1:NTests
    for sentence=1:NSentences
        idx = (test-1)*NSentences+sentence;
        cond_Acc = ismember(idx,data.acc);      % The trial is accepted
        % The trial is SRT80-QOmni, SRT80-DM, SRT95-QOmni, or SRT95-DM
        cond_SRT80_QOmni = ismember((floor(idx/NSentences)+1),...
            find(TestCond(Participant,:)==1)); % SRT80-QOmni
        cond_SRT80_DM = ismember((floor(idx/NSentences)+1),...
            find(TestCond(Participant,:)==2)); % SRT80-DM
        cond_SRT95_QOmni = ismember((floor(idx/NSentences)+1),...
            find(TestCond(Participant,:)==3)); % SRT95-QOmni
        cond_SRT95_DM = ismember((floor(idx/NSentences)+1),...
            find(TestCond(Participant,:)==4)); % SRT95-DM
        % Determine valid Trials in all test conditions
        TrialsSRT80_QOmni(idx) = cond_Acc & cond_SRT80_QOmni;
        TrialsSRT80_DM(idx) = cond_Acc & cond_SRT80_DM;
        TrialsSRT95_QOmni(idx) = cond_Acc & cond_SRT95_QOmni;
        TrialsSRT95_DM(idx) = cond_Acc & cond_SRT95_DM;
    end
end
clear idx cond_Acc cond_SRT80_QOmni
```

```
clear cond_SRT80_DM cond_SRT95_QOmni cond_SRT95_DM

% TF analysis configuration
cfg = [];
cfg.method = 'wavelet';                   % Morlet wavelet
cfg.width = 5;                            % Number of cycles of the wavelet
cfg.foilim = [0 FMax];                    % Frequency band of interest
cfg.toi = -2:0.05:L_Window-2;             % Time axis
cfg.keeptrials = 'yes';                   % Keeps the trials (heavy files)
cfg.channel = {'all','-VEO','-HEO'};      % Exclude ocular channels
cfg.output = 'pow';                       % Power spectra

% TF analysis for SRT-80 QOmni (TestCond = 1)
cfg.trials = find(TrialsSRT80_QOmni);
data_TFR_SRT80_QOmni = ft_freqanalysis(cfg,data);

% TF analysis for SRT-80 DM (TestCond = 2)
cfg.trials = find(TrialsSRT80_DM);
data_TFR_SRT80_DM = ft_freqanalysis(cfg,data);

% TF analysis for SRT-95 QOmni (TestCond = 3)
cfg.trials = find(TrialsSRT95_QOmni);
data_TFR_SRT95_QOmni = ft_freqanalysis(cfg,data);

% TF analysis for SRT-95 DM (TestCond = 4)
cfg.trials = find(TrialsSRT95_DM);
data_TFR_SRT95_DM = ft_freqanalysis(cfg,data);

%% Visualise the mean Power spectrum across electrodes
figure('PaperSize',[30 20])
colormap('jet');
cfg = [];
cfg.figure = 'gca';
cfg.ylim = [0 FMax];
cfg.zlim = [0 2000];
cfg.layout = 'EEG1010.lay';
subplot(2,2,1)
ft_singleplotTFR(cfg,data_TFR_SRT80_DM)
xlabel('Time (s)')
ylabel('Frequency (Hz)')
title('SRT-80 [DM]')
text(-1.5,FMax+4,['P0' SBJ(Participant,:) ' - Power Spectrum'])
subplot(2,2,2)
ft_singleplotTFR(cfg,data_TFR_SRT95_DM)
xlabel('Time (s)')
ylabel('Frequency (Hz)')
title('SRT-95 [DM]')
subplot(2,2,3)
ft_singleplotTFR(cfg,data_TFR_SRT80_QOmni)
xlabel('Time (s)')
```

```
ylabel('Frequency (Hz)')
title('SRT-80 [Q-Omni]')
subplot(2,2,4)
ft_singleplotTFR(cfg,data_TFR_SRT95_QOmni)
xlabel('Time (s)')
ylabel('Frequency (Hz)')
title('SRT-95 [Q-Omni]')
orient tall
print('-dpng','-r300',['Figures/' SBJ(Participant,:) '_PowSpec'])
close all

%% Get the Mean Averages (MA)
cfg = [];
cfg.keeptrials = 'no';
cfg.channel = {'all','-VEO','-HEO'};
cfg.trials = 'all';
cfg.frequency = 'all';
cfg.latency = 'all';
MA_SRT80_QOmni = ft_freqdescriptives(cfg,data_TFR_SRT80_QOmni);
MA_SRT80_DM = ft_freqdescriptives(cfg,data_TFR_SRT80_DM);
MA_SRT95_QOmni = ft_freqdescriptives(cfg,data_TFR_SRT95_QOmni);
MA_SRT95_DM = ft_freqdescriptives(cfg,data_TFR_SRT95_DM);

%% Store data
save(['Results/P0' SBJ(Participant,:)],'MA_SRT80_QOmni','MA_SRT80_DM',...
    'MA_SRT95_QOmni','MA_SRT95_DM','TrialsSRT80_QOmni','TrialsSRT80_DM',...
    'TrialsSRT95_QOmni','TrialsSRT95_DM')
```

6 MATLAB script that performs a cluster-based permutational test on alpha power

```
%% Cluster permutation analysis on aggregated data from 19 participants
clear,clc
addpath('FieldTrip')                   % Add the FieldTrip directory

%% Combine individual Mean Averages (MAs)
% P06 excluded due to corrupted triggers
SBJ = ['01';'02';'03';'04';'05';'07';'08';'09';'10';'11';'12';...
    '13';'14';'15';'16';'17';'18';'19';'20'];
NSbj = length(SBJ);
All_SRT80_QOmni = cell(NSbj,1);       % PowSpec   SRT-80 Q-Omni
All_SRT80_DM = cell(NSbj,1);          % PowSpec   SRT-80 DM
All_SRT95_QOmni = cell(NSbj,1);       % PowSpec   SRT-95 Q-Omni
All_SRT95_DM = cell(NSbj,1);          % PowSpec   SRT-95 DM
for sbj=1:NSbj
    fprintf(sprintf('Subject %d/%d\n',sbj,NSbj))
    load(['Results/P0' SBJ(sbj,:)])
    All_SRT80_QOmni{sbj} = MA_SRT80_QOmni;
    All_SRT80_DM{sbj} = MA_SRT80_DM;
    All_SRT95_QOmni{sbj} = MA_SRT95_QOmni;
    All_SRT95_DM{sbj} = MA_SRT95_DM;
end

%% Grand-averages across subjects
cfg = [];
cfg.keepindividual = 'yes';
cfg.foilim = 'all';
cfg.toilim = 'all';
cfg.channel = 'all';
cfg.parameter = 'powspctrm';

GA_SRT80_QOmni = ft_freqgrandaverage(cfg,All_SRT80_QOmni{1},...
    All_SRT80_QOmni{2},All_SRT80_QOmni{3},All_SRT80_QOmni{4},...
    All_SRT80_QOmni{5},All_SRT80_QOmni{6},All_SRT80_QOmni{7},...
    All_SRT80_QOmni{8},All_SRT80_QOmni{9},All_SRT80_QOmni{10},...
    All_SRT80_QOmni{11},All_SRT80_QOmni{12},All_SRT80_QOmni{13},...
    All_SRT80_QOmni{14},All_SRT80_QOmni{15},All_SRT80_QOmni{16},...
    All_SRT80_QOmni{17},All_SRT80_QOmni{18},All_SRT80_QOmni{19});
GA_SRT80_DM = ft_freqgrandaverage(cfg,All_SRT80_DM{1},...
    All_SRT80_DM{2},All_SRT80_DM{3},All_SRT80_DM{4},...
    All_SRT80_DM{5},All_SRT80_DM{6},All_SRT80_DM{7},...
    All_SRT80_DM{8},All_SRT80_DM{9},All_SRT80_DM{10},...
    All_SRT80_DM{11},All_SRT80_DM{12},All_SRT80_DM{13},...
    All_SRT80_DM{14},All_SRT80_DM{15},All_SRT80_DM{16},...
    All_SRT80_DM{17},All_SRT80_DM{18},All_SRT80_DM{19});
GA_SRT95_QOmni = ft_freqgrandaverage(cfg,All_SRT95_QOmni{1},...
    All_SRT95_QOmni{2},All_SRT95_QOmni{3},All_SRT95_QOmni{4},...
    All_SRT95_QOmni{5},All_SRT95_QOmni{6},All_SRT95_QOmni{7},...
    All_SRT95_QOmni{8},All_SRT95_QOmni{9},All_SRT95_QOmni{10},...
```

```
    All_SRT95_QOmni{11},All_SRT95_QOmni{12},All_SRT95_QOmni{13},...
    All_SRT95_QOmni{14},All_SRT95_QOmni{15},All_SRT95_QOmni{16},...
    All_SRT95_QOmni{17},All_SRT95_QOmni{18},All_SRT95_QOmni{19});
GA_SRT95_DM = ft_freqgrandaverage(cfg,All_SRT95_DM{1},...
    All_SRT95_DM{2},All_SRT95_DM{3},All_SRT95_DM{4},...
    All_SRT95_DM{5},All_SRT95_DM{6},All_SRT95_DM{7},...
    All_SRT95_DM{8},All_SRT95_DM{9},All_SRT95_DM{10},...
    All_SRT95_DM{11},All_SRT95_DM{12},All_SRT95_DM{13},...
    All_SRT95_DM{14},All_SRT95_DM{15},All_SRT95_DM{16},...
    All_SRT95_DM{17},All_SRT95_DM{18},All_SRT95_DM{19});

%% Cluster analysis
% Neighbour channels
cfg = [];
cfg.layout = 'EEG1010.lay';
lay = ft_prepare_layout(cfg);
cfg.layout = lay;
cfg.method = 'triangulation';
neigh = ft_prepare_neighbours(cfg);     % Prepare neighbour channels

% Cluster Permutation Analysis (configuration)
cfg = [];
cfg.layout = 'EEG1010.lay';
cfg.neighbours = neigh;
cfg.minnbchan = 2;                      % Min number of chans per cluster
cfg.frequency = [8.0 12.0];             % Alpha frequency range
cfg.channel  = {'all'};                 % Channels
cfg.parameter = 'powspctrm';            % Analysis on the power spectrum
cfg.avgovertime = 'yes';                % We average over the time domain
cfg.avgoverfreq = 'yes';                % We average over the freq domain
cfg.avgoverchan = 'no';                 % We keep separated info on chans
cfg.statistic = 'ft_statfun_depsamplesT';   % Paired test
cfg.numrandomization = 'all';           % All possible permutations
cfg.method = 'montecarlo';
cfg.correctm = 'cluster';               % Correct for multiple comparisons
cfg.computeprob = 'yes';
cfg.computecritval = 'yes';
cfg.clusterthreshold = 'nonparametric_common';
cfg.clusteralpha = 0.05;
cfg.clusterstatistic = 'maxsum';
cfg.clustertail = 0;
cfg.tail = 0;
cfg.alpha = 0.05;
    NSbj = 19;
    design(1,1:2*NSbj) = repmat(1:NSbj,[1,2]);
    design(2,1:2*NSbj) = [ones(1,NSbj),2*ones(1,NSbj)];
cfg.design = design;
cfg.uvar = 1;
cfg.ivar = 2;
cfg.spmversion = 'spm12';
```

```
% Selection of time interval and SRT
Time_Interval = 'PreStim';                % Time interval selection
switch Time_Interval
    case 'PreStim',     cfg.latency = [-1.5 0.0];
    case 'Stimulus',    cfg.latency = [0.0 2.0];
    case 'Encoding',    cfg.latency = [2.0 3.5];
    case 'Retention',   cfg.latency = [3.5 5.0];
end
SRT = 'SRT-80';
switch SRT
    case 'SRT-80'
        QOmni_Data = GA_SRT80_QOmni;
        DM_Data = GA_SRT80_DM;
    case 'SRT-95'
        QOmni_Data = GA_SRT95_QOmni;
        DM_Data = GA_SRT95_DM;
end

% Cluster permutation analysis
stat = ft_freqstatistics(cfg,QOmni_Data,DM_Data);

%% Visualization of selected clusters
cfg = [];
cfg.alpha = 0.05;
cfg.parameter = 'stat';
cfg.zlim = [-4 4];
cfg.layout = 'EEG1010.lay';
cfg.subplotsize = [1 1];
ft_clusterplot(cfg,stat); colormap(jet)
```

7 Assigned colours to each participant in Figures 3, 4 and 5

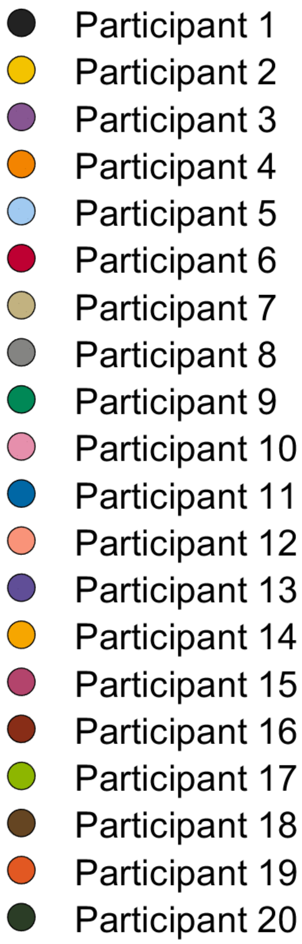

8 Individual spectrograms

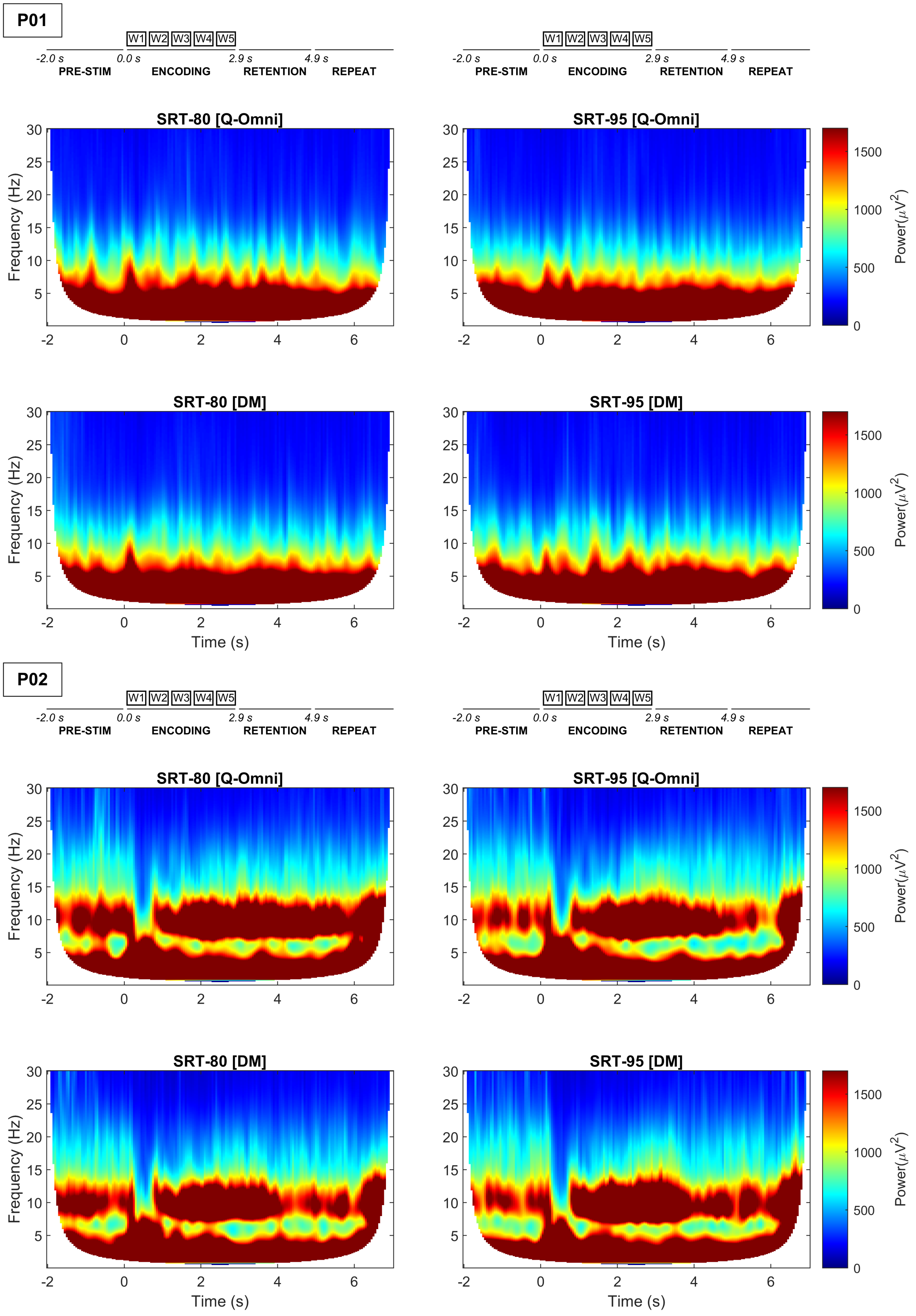

Figure 8: Individual spectrograms from the #P01 and #P02 participants.

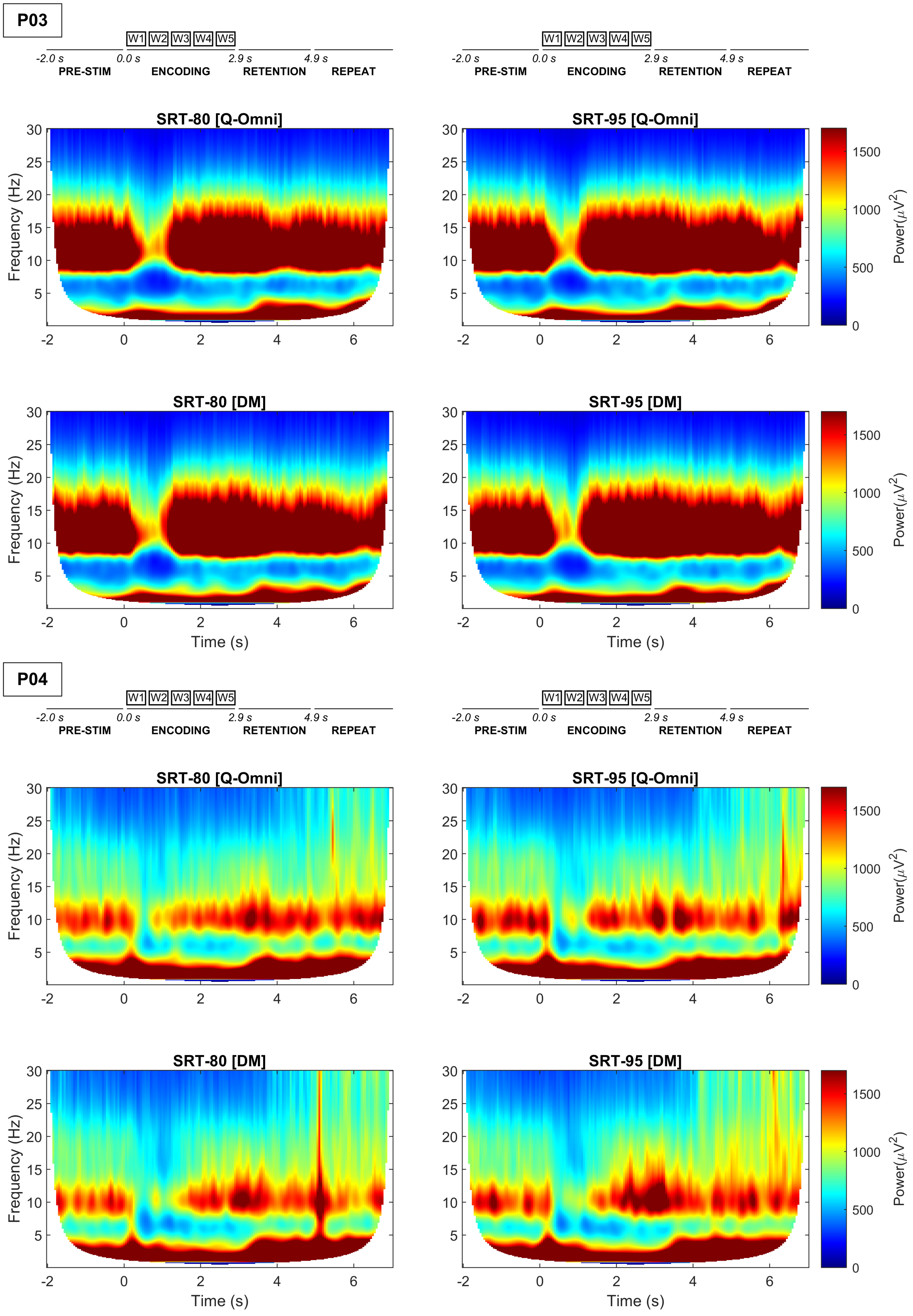

Figure 9: Individual spectrograms from the #P03 and #P04 participants.

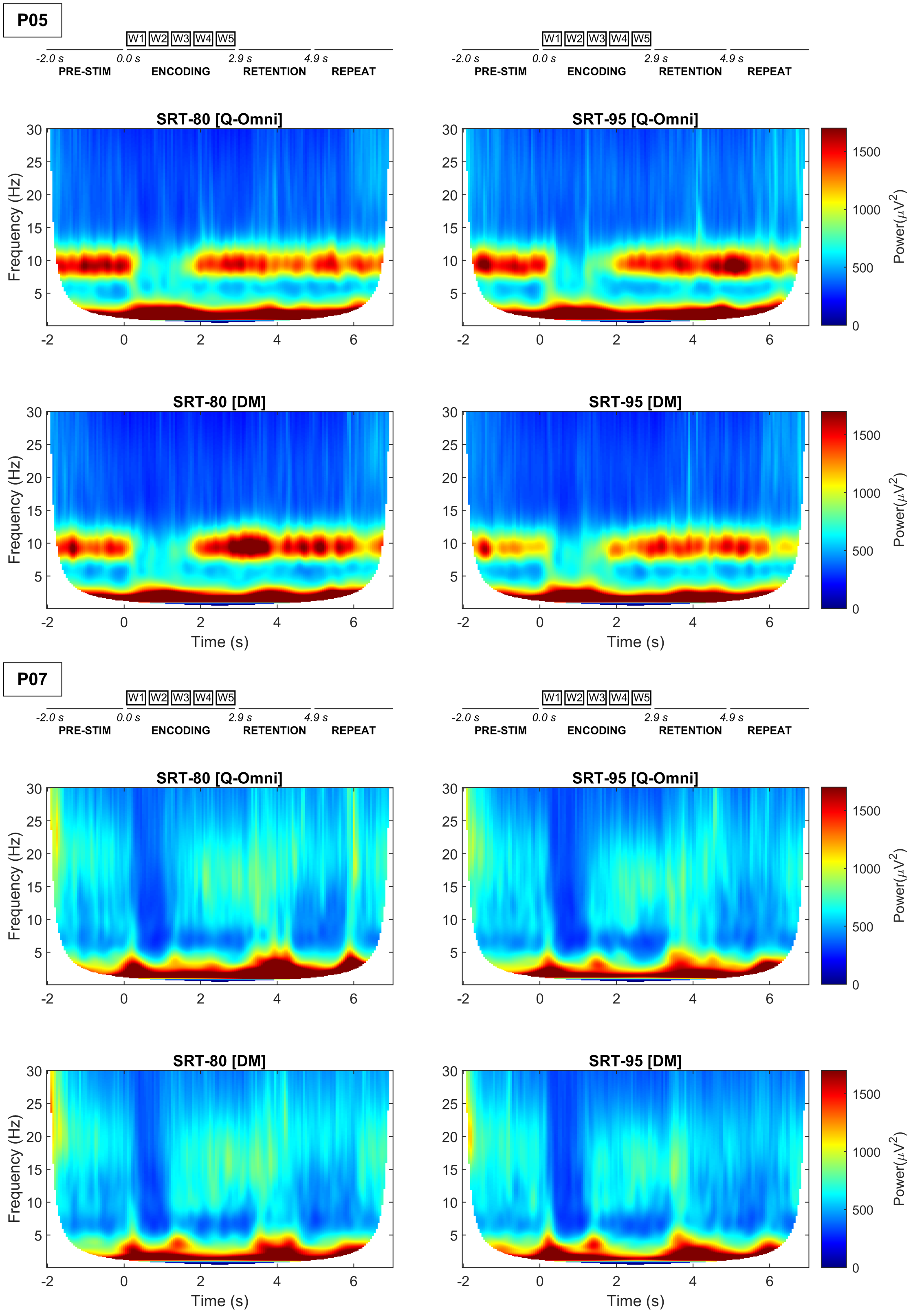

Figure 10: Individual spectrograms from the #P05 and #P07 participants.

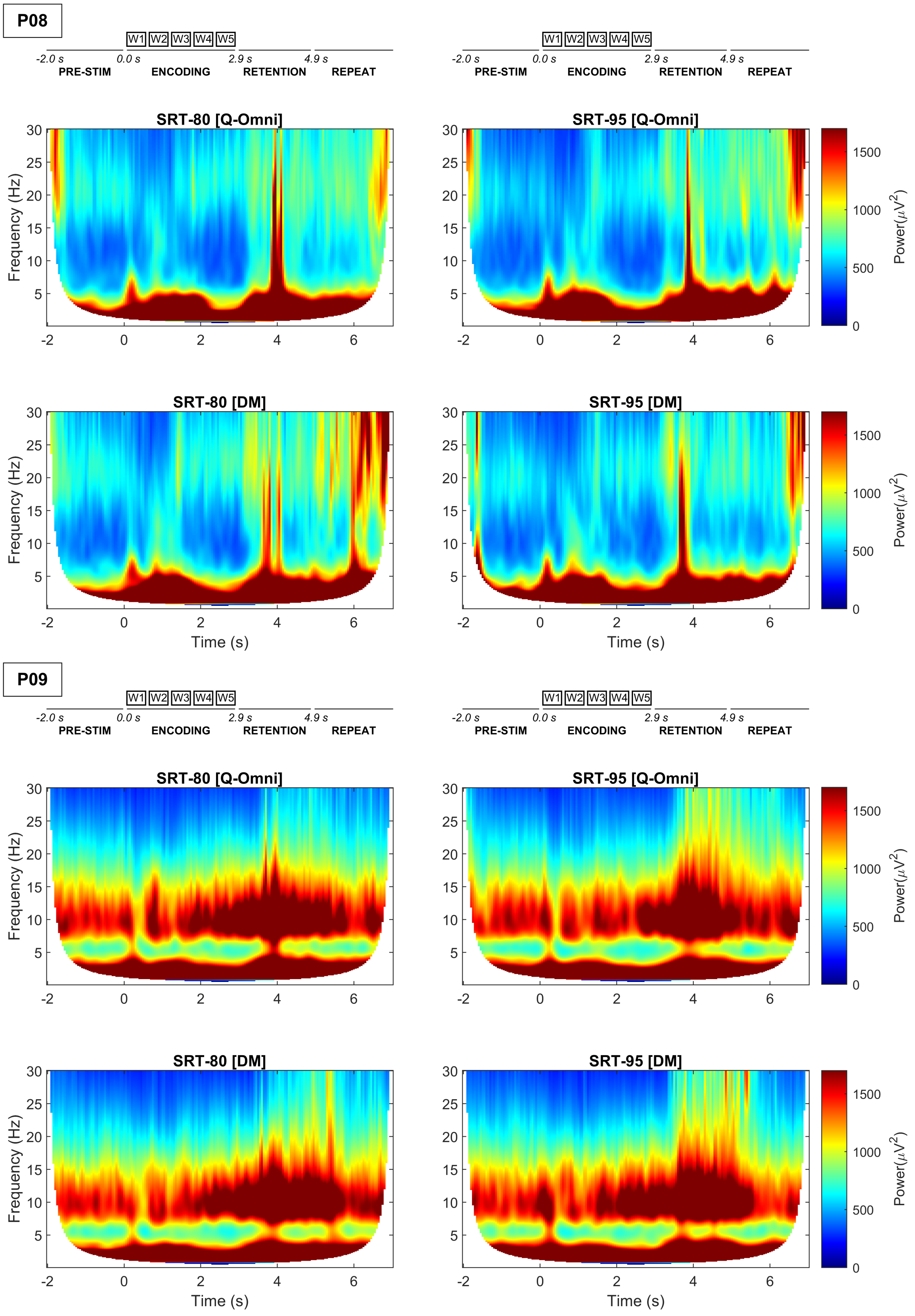

Figure 11: Individual spectrograms from the #P08 and #P09 participants.

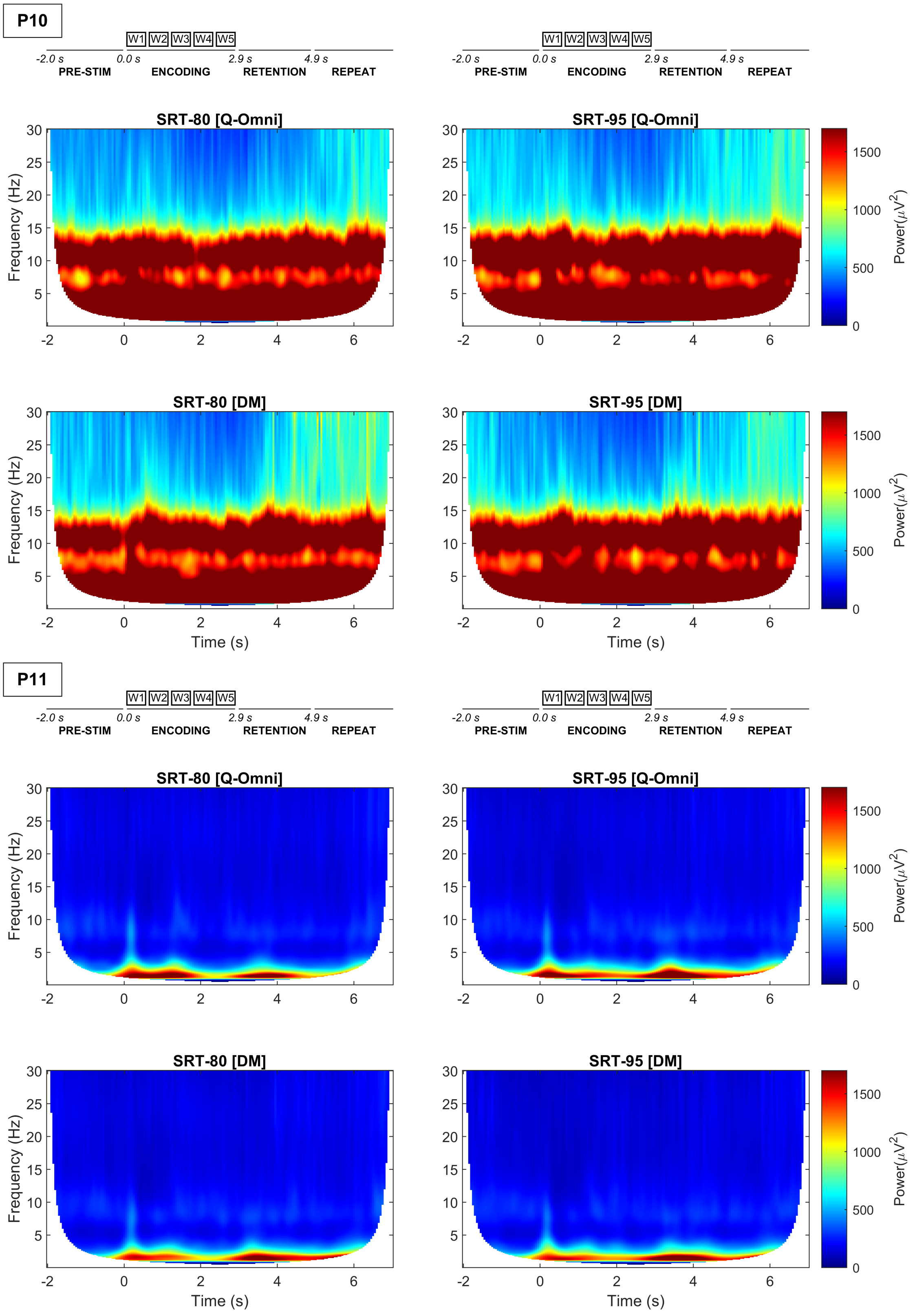

Figure 12: Individual spectrograms from the #P10 and #P11 participants.

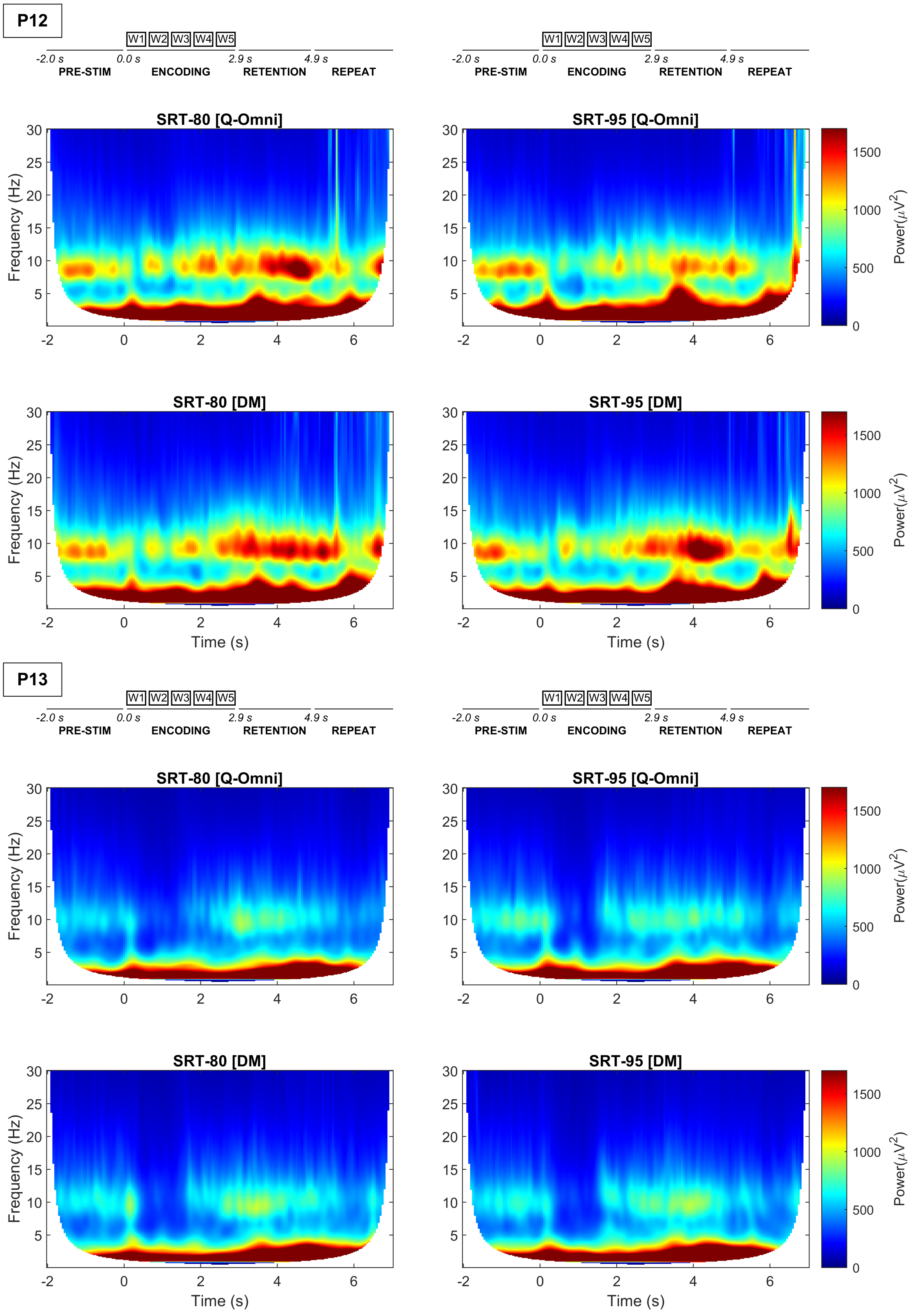

Figure 13: Individual spectrograms from the #P12 and #P13 participants.

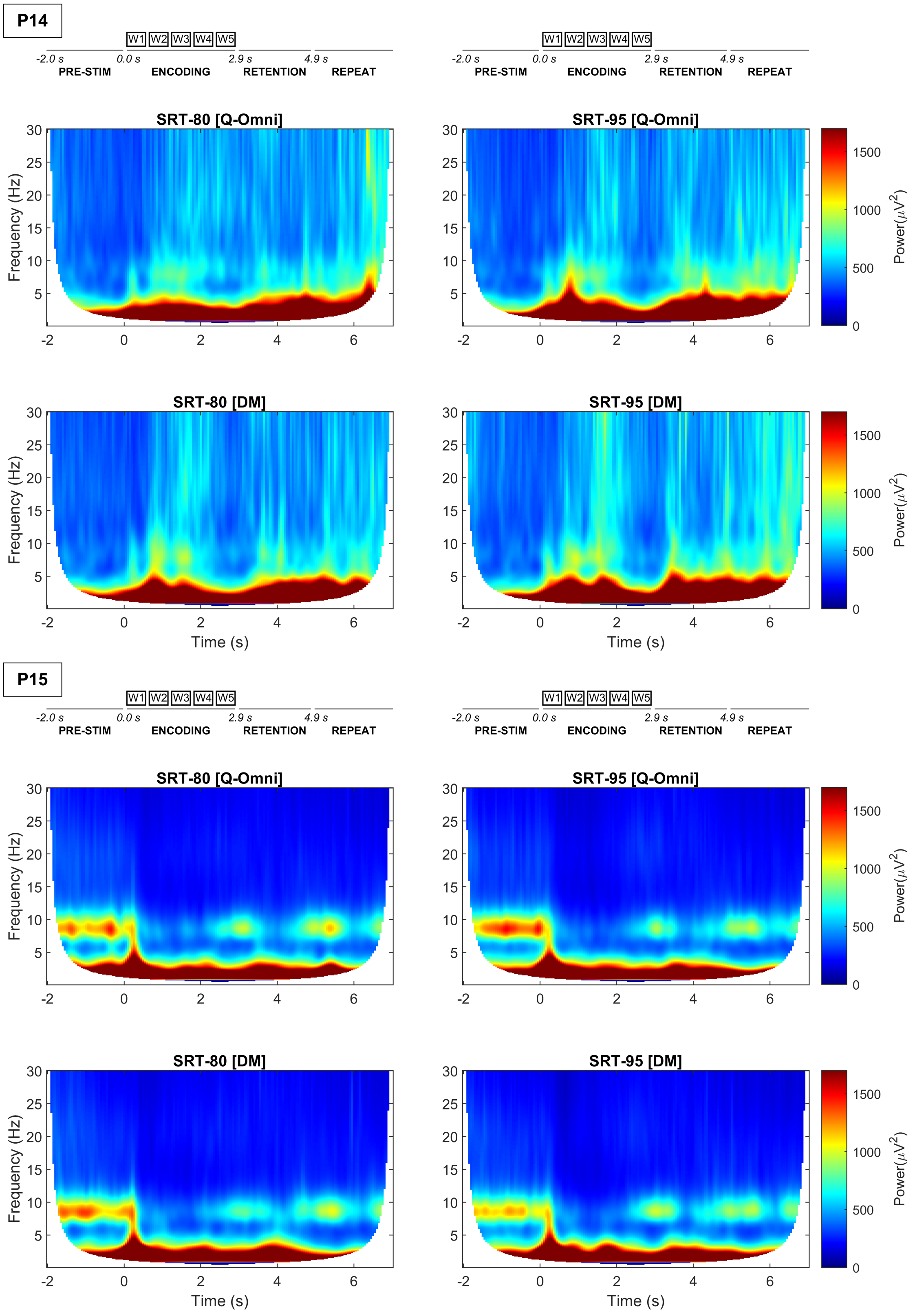

Figure 14: Individual spectrograms from the #P14 and #P15 participants.

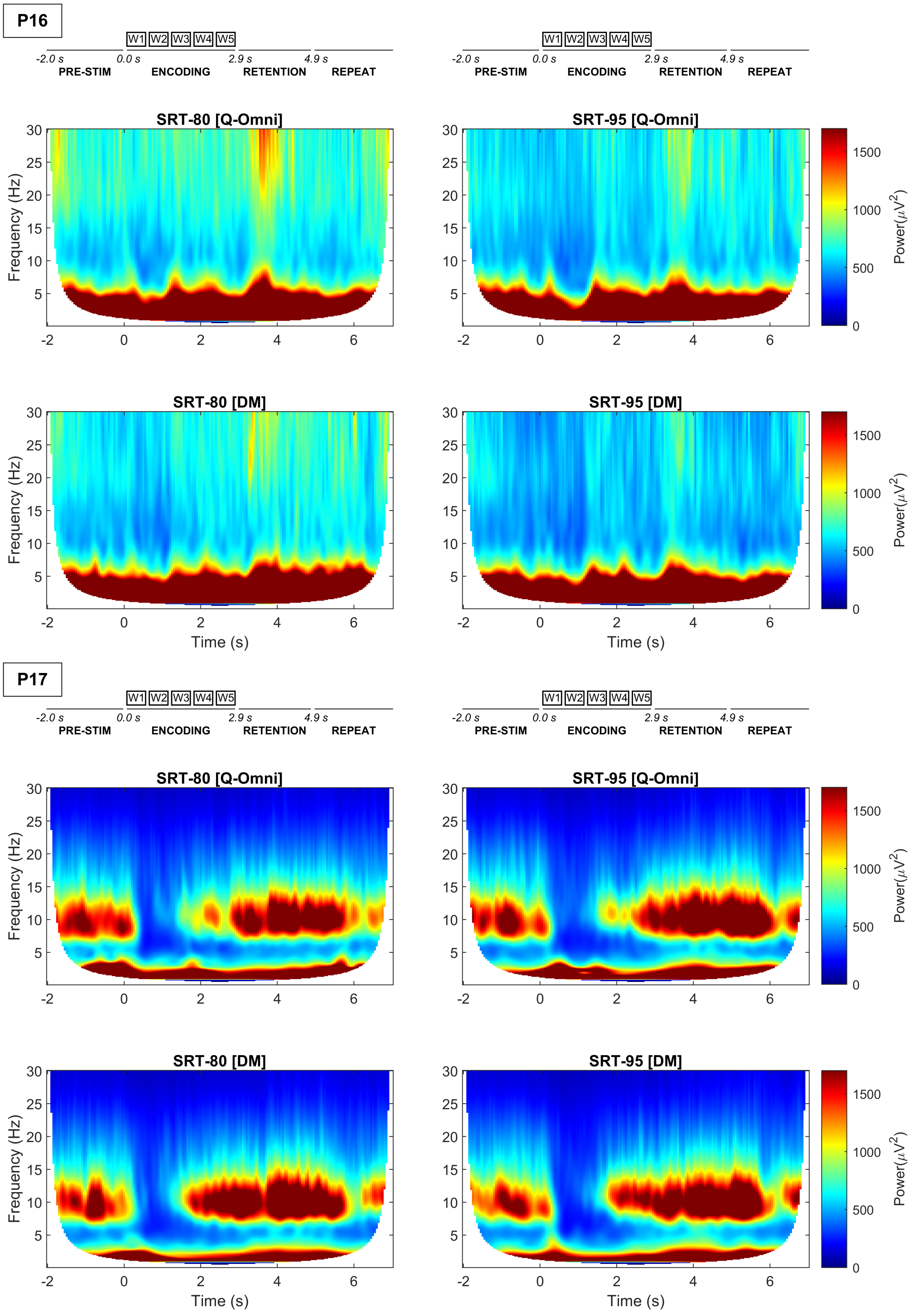

Figure 15: Individual spectrograms from the #P16 and #P17 participants.

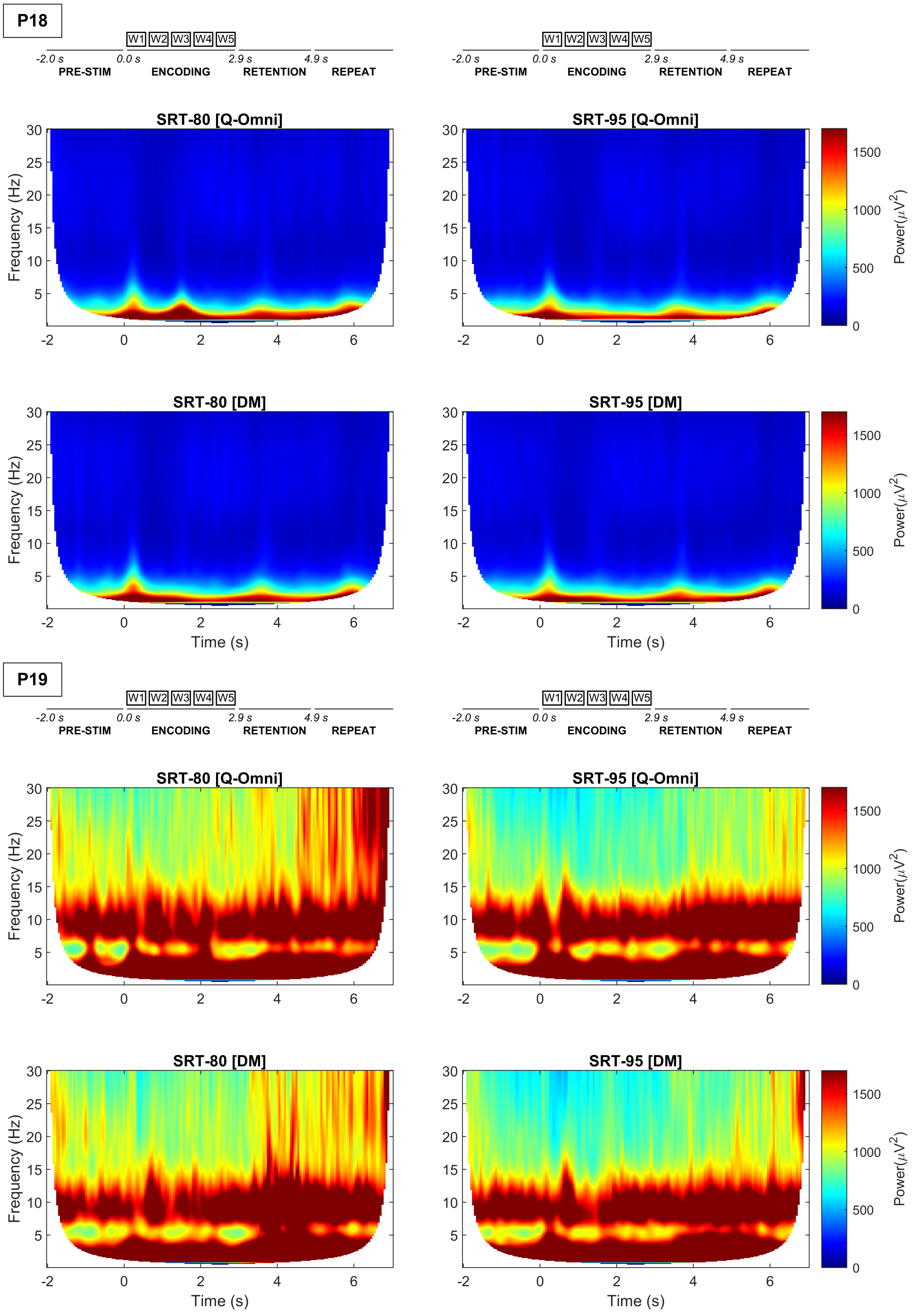

Figure 16: Individual spectrograms from the #P18 and #P19 participants.

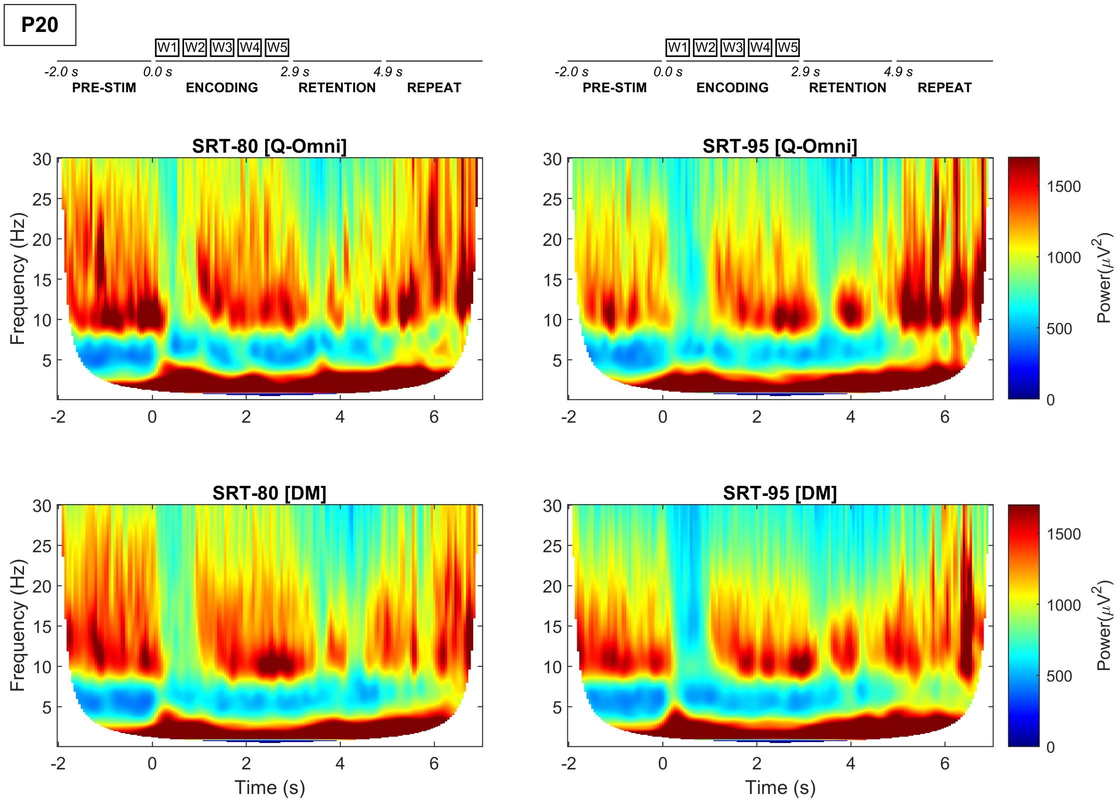

Figure 17: Individual spectrograms from the #P20 participant.

References

L. L. Beranek. *Acoustics McGraw-Hill – Electrical and Electronic Engineering series*. McGraw-Hill, New York, NY, 1954.

B. Hagerman and A. Olofsson. A method to measure the effect of noise reduction algorithms using simultaneous speech and noise. *Acta Acustica united with Acustica*, 90:356–361, 2004. doi: https://www.ingentaconnect.com/content/dav/aaua/2004/00000090/00000002/art00016#.

J. M. Kates. *Digital hearing aids*. Plural Publishing Inc., San Diego, CA, 2008.

G. Keidser, H. Dillon, L. Carter, and A. O'Brien. NAL-NL2 empirical adjustments. *Trends in Amplification*, 16:211–223, 2012. doi: https://doi.org/10.1177/1084713812468511.

H. Kelly, G. Lin, N. Sankaran, J. Xia, S. Kalluri, and S. Carlile. Development and evaluation of a mixed gender, multi-talker matrix sentence test in Australian English. *International Journal of Audiology*, 56:85–91, 2017. doi: https://doi.org/10.1080/14992027.2016.1236415.

M. Killion, R. Schulien, L. Christensen, D. Fabry, L. Revit, P. Niquette, and K. Chung. Real-world performance of an ITE directional microphone. *The Hearing Journal*, 51:24–38, 1998. doi: https://www.etymotic.com/wp-content/uploads/2021/05/erl-0038-1998.pdf.

Z. S. Nasreddine, N. A. Phillips, V. Bédirian, S. Charbonneau, V. Whitehead, I. Collin, J. L. Cummings, and H. Chertkow. The Montreal Cognitive Assessment, MoCA: a brief screening tool for mild cognitive impairment. *Journal of the American Geriatrics Society*, 53:695–699, 2005. doi: https://doi.org/10.1111/j.1532-5415.2005.53221.x.

A. Weisser, J. M. Buchholz, C. Oreinos, J. Badajoz-Davila, J. Galloway, T. Beechey, and G. Keidser. The Ambisonic Recordings of Typical Environments (ARTE) Database. *Acta Acustica united with Acustica*, 105:695–713, 2019. doi: https://doi.org/10.3813/AAA.919349.
