# Supplementary figures and images for: Reduced listening effort with adaptive binaural beamforming in realistic noisy environments
Source: Sci Rep. 2025 May 23;15:17998. doi: 10.1038/s41598-025-95045-3 (PMC12102238; doi:10.1038/s41598-025-95045-3)

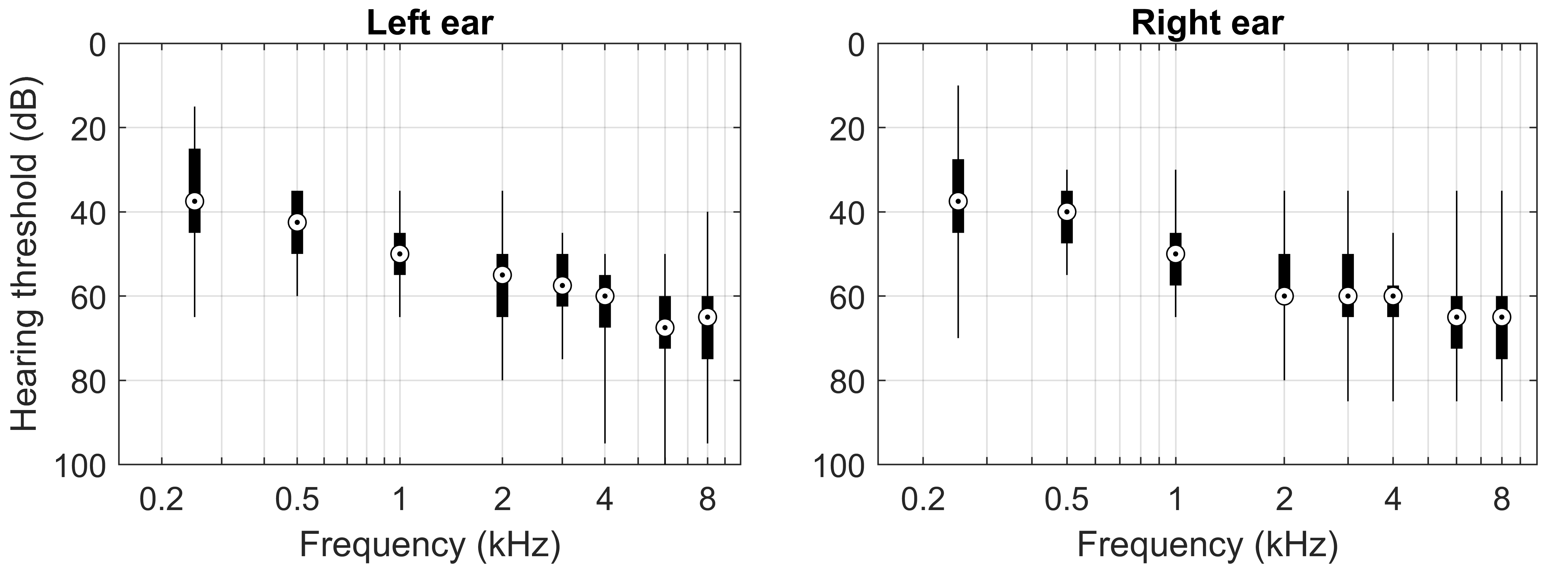

Supplement: Supplementary file 1 — Supplementary Information. [file 41598_2025_95045_MOESM1_ESM.zip › Appendix_B/Figure1/Figure1.png]

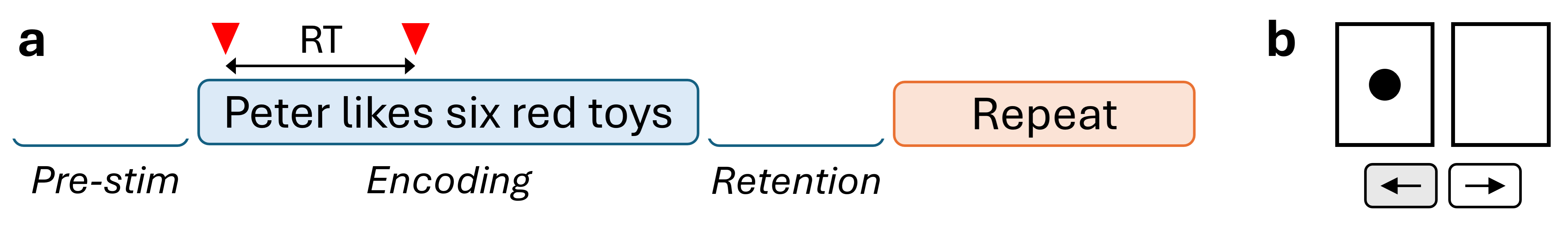

Supplement: Supplementary file 1 — Supplementary Information. [file 41598_2025_95045_MOESM1_ESM.zip › Appendix_B/Figure2/Figure2.png]

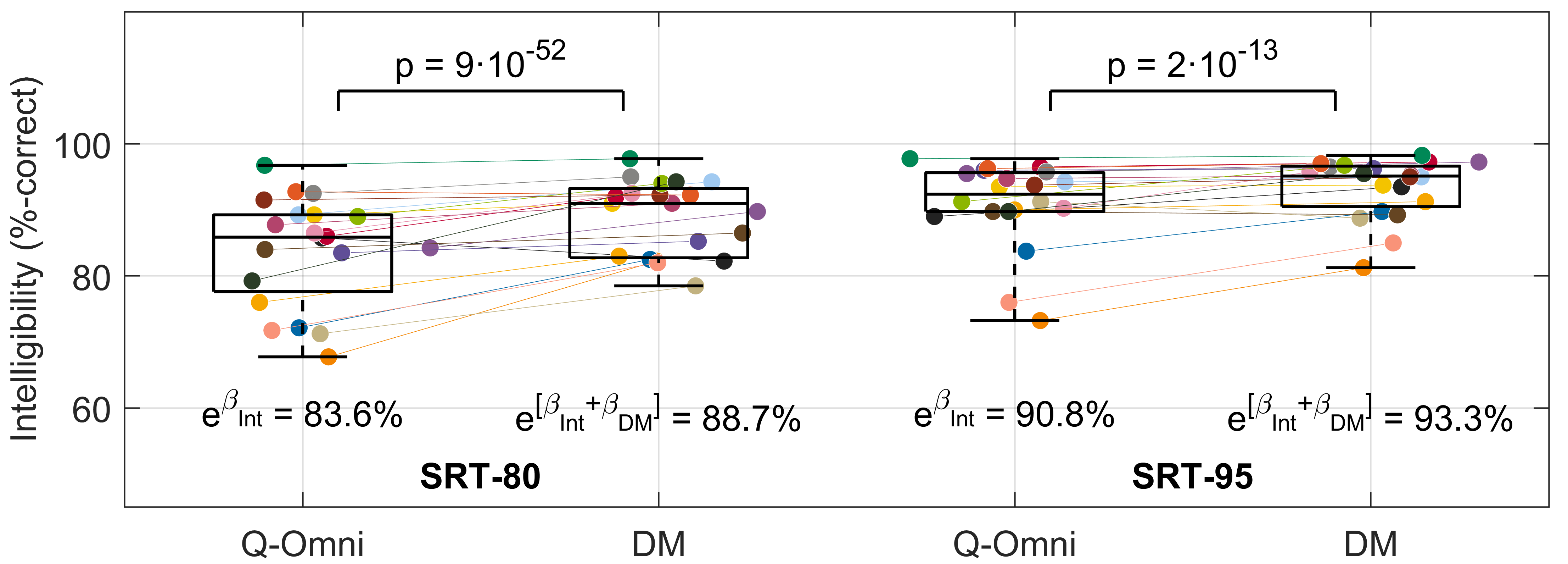

Supplement: Supplementary file 1 — Supplementary Information. [file 41598_2025_95045_MOESM1_ESM.zip › Appendix_B/Figure3/Figure3.png]

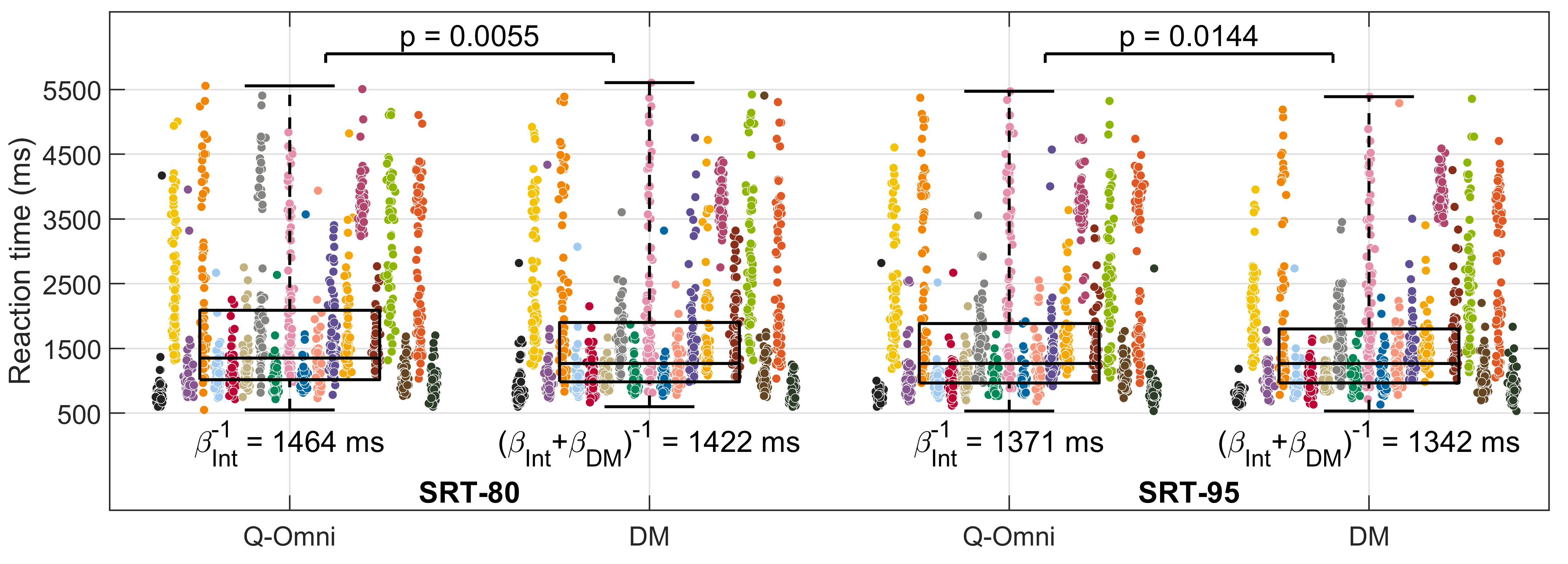

Supplement: Supplementary file 1 — Supplementary Information. [file 41598_2025_95045_MOESM1_ESM.zip › Appendix_B/Figure4/Figure4.png]

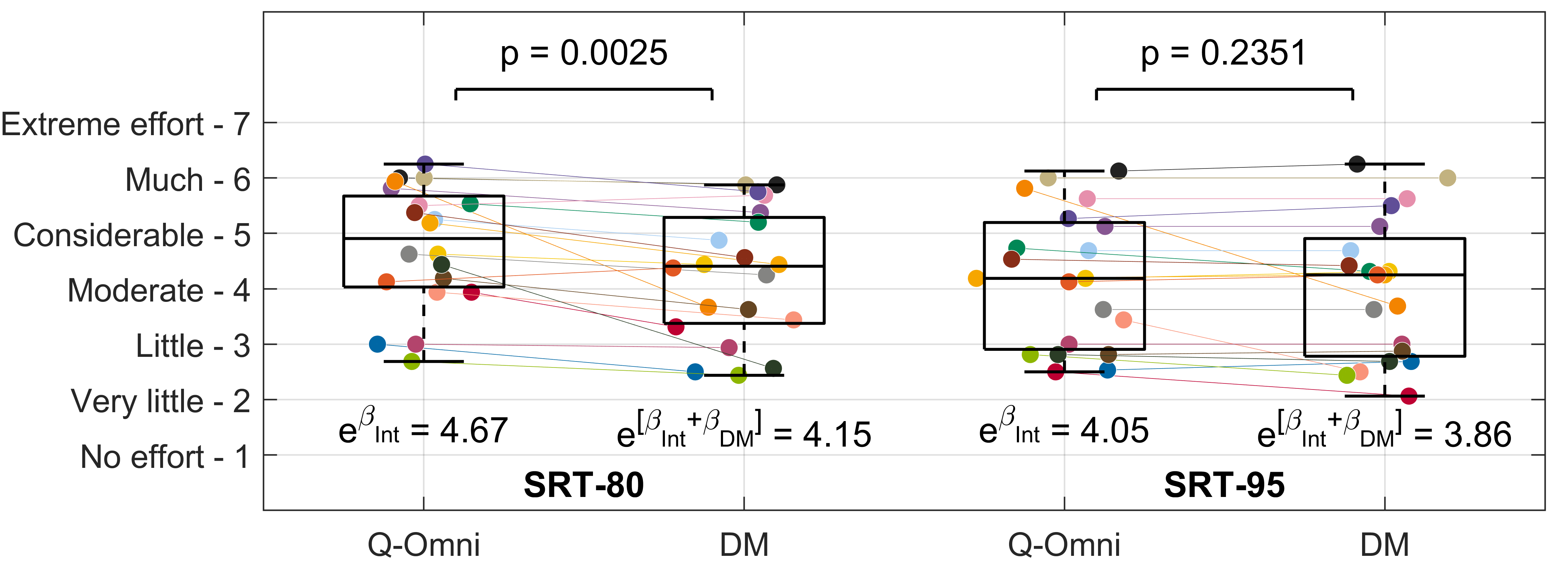

Supplement: Supplementary file 1 — Supplementary Information. [file 41598_2025_95045_MOESM1_ESM.zip › Appendix_B/Figure5/Figure5.png]

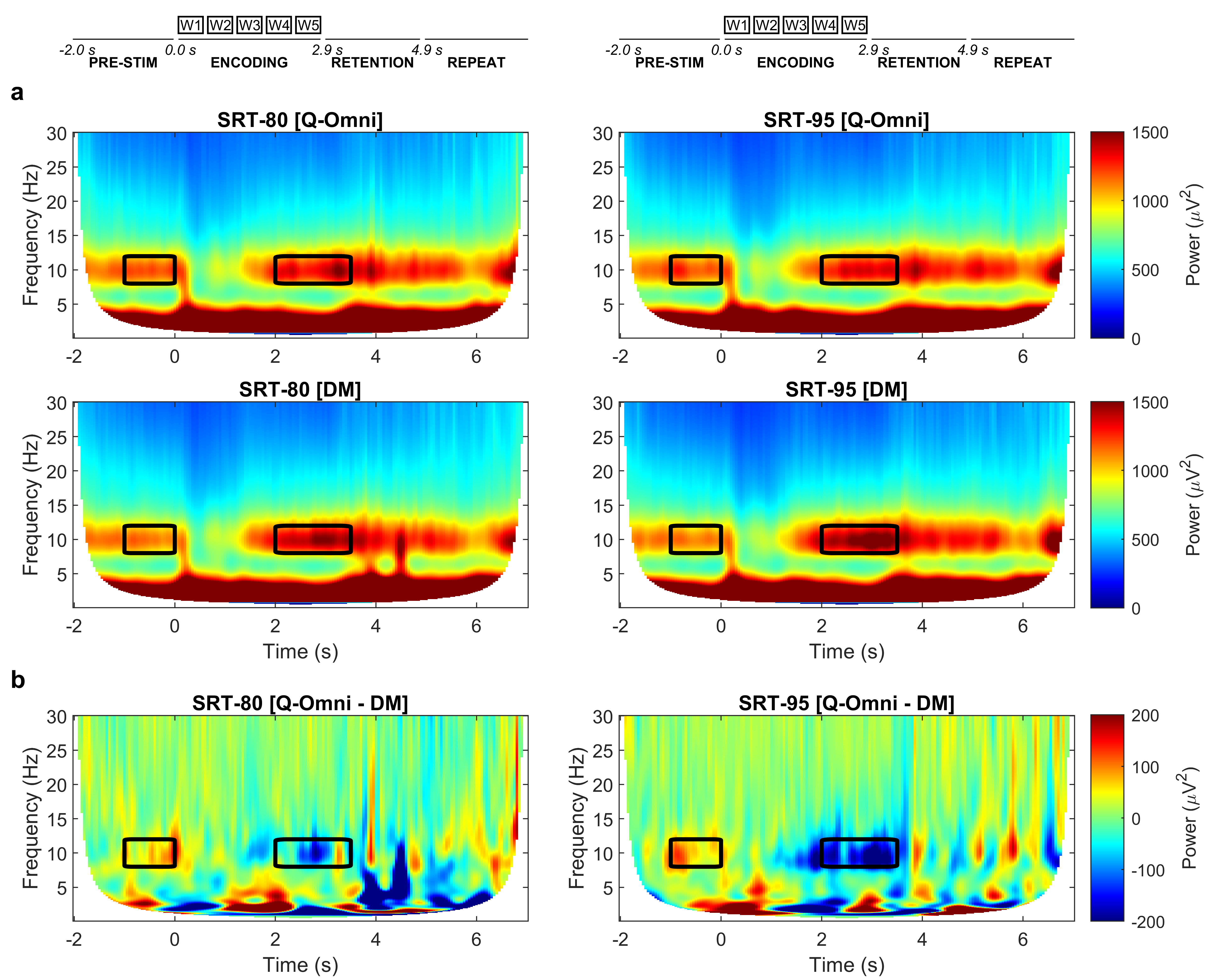

Supplement: Supplementary file 1 — Supplementary Information. [file 41598_2025_95045_MOESM1_ESM.zip › Appendix_B/Figure6/Figure6.png]

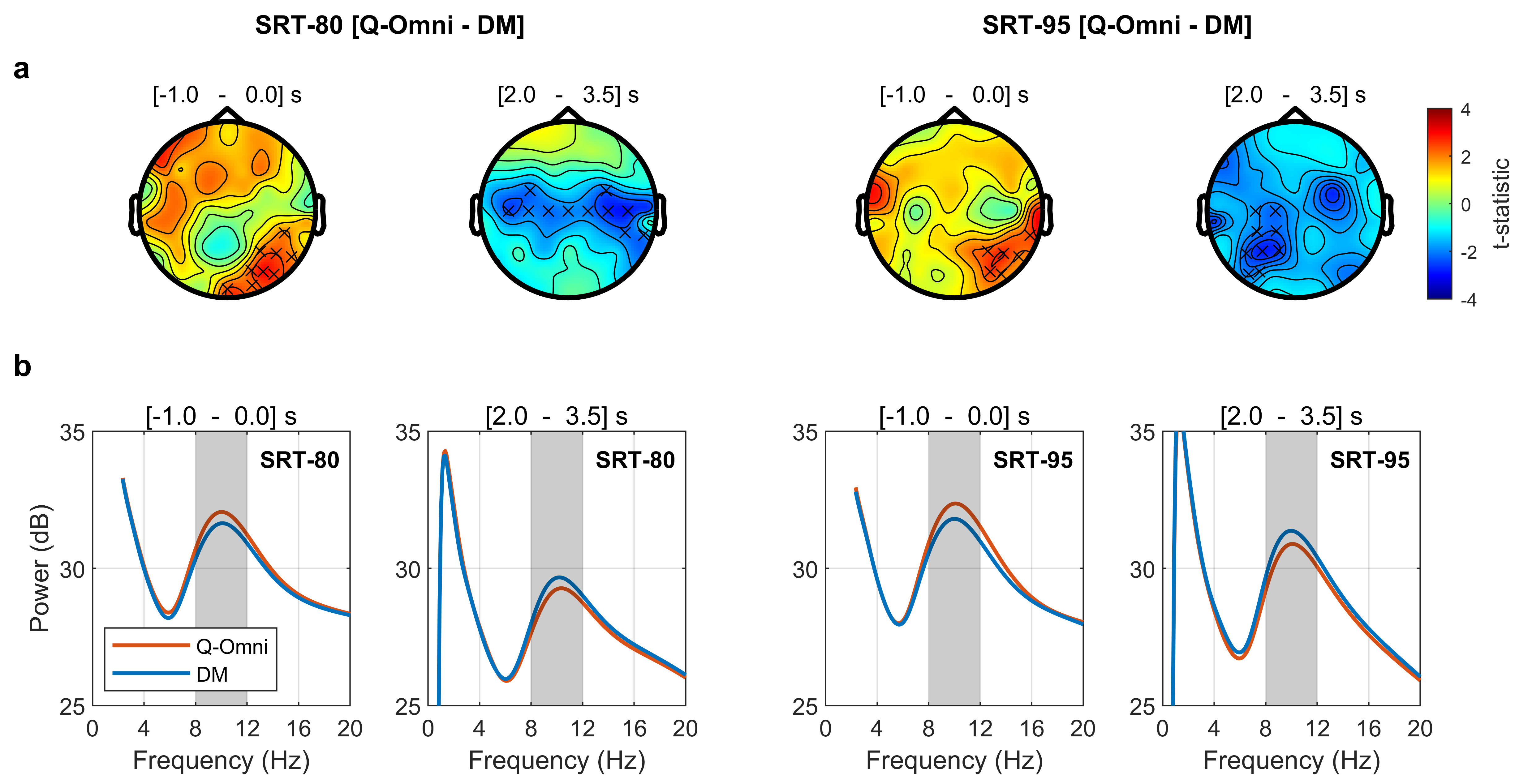

Supplement: Supplementary file 1 — Supplementary Information. [file 41598_2025_95045_MOESM1_ESM.zip › Appendix_B/Figure7/Figure7.png]

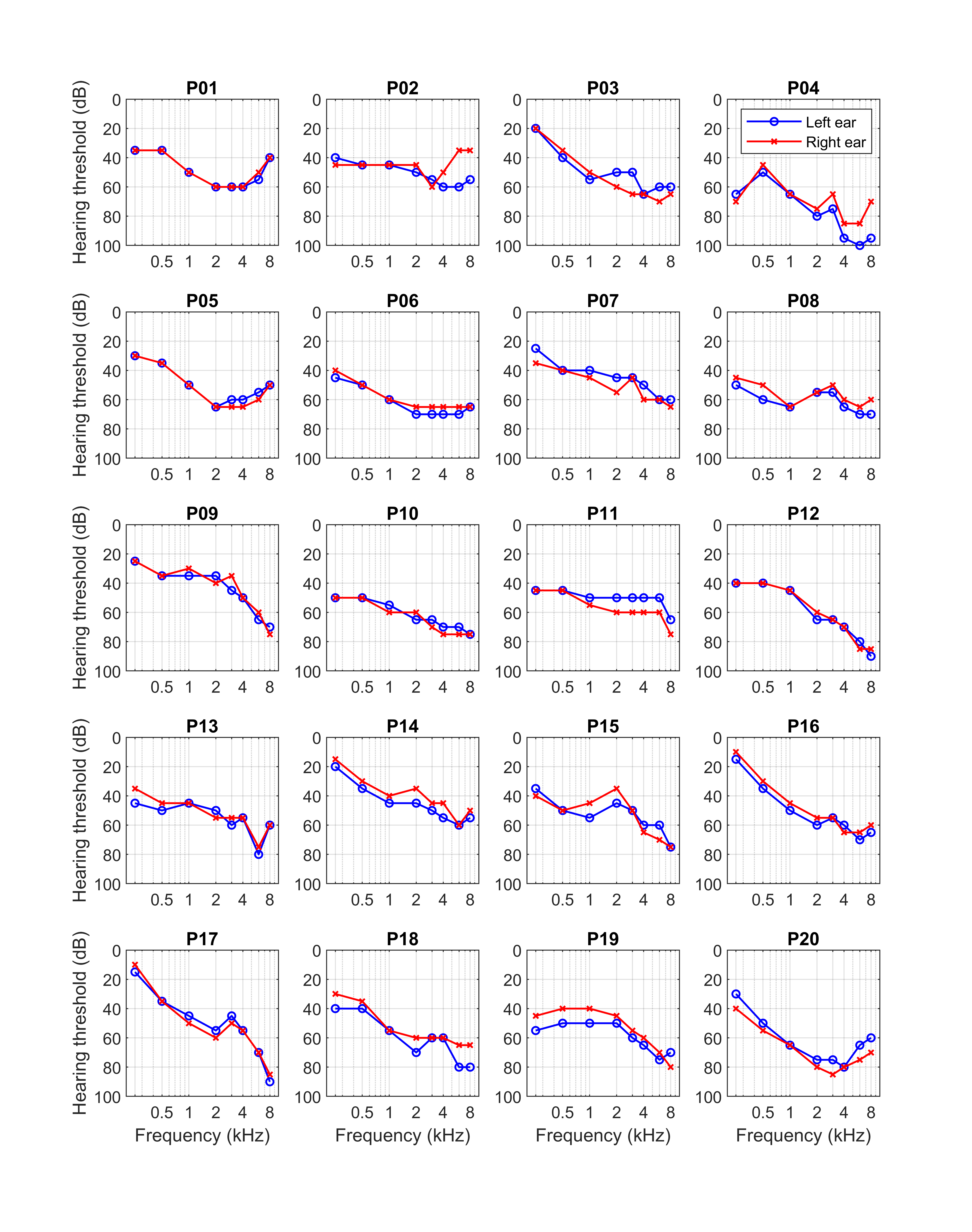

Supplement: Supplementary file 1 — Supplementary Information. [file 41598_2025_95045_MOESM1_ESM.zip › Appendix_B/FigureA1/FigureA1.png]

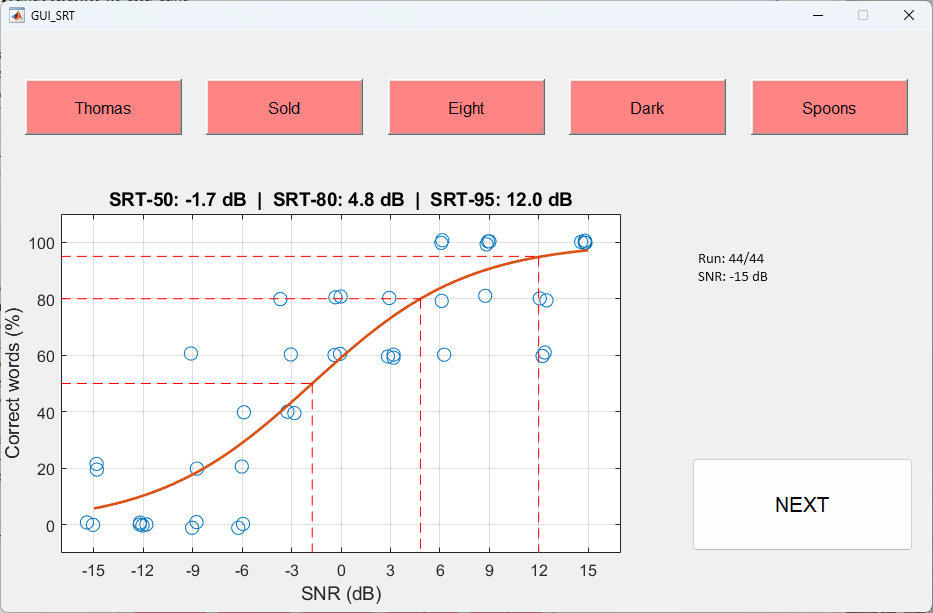

Supplement: Supplementary file 1 — Supplementary Information. [file 41598_2025_95045_MOESM1_ESM.zip › Appendix_B/FigureA2/FigureA2a.png]

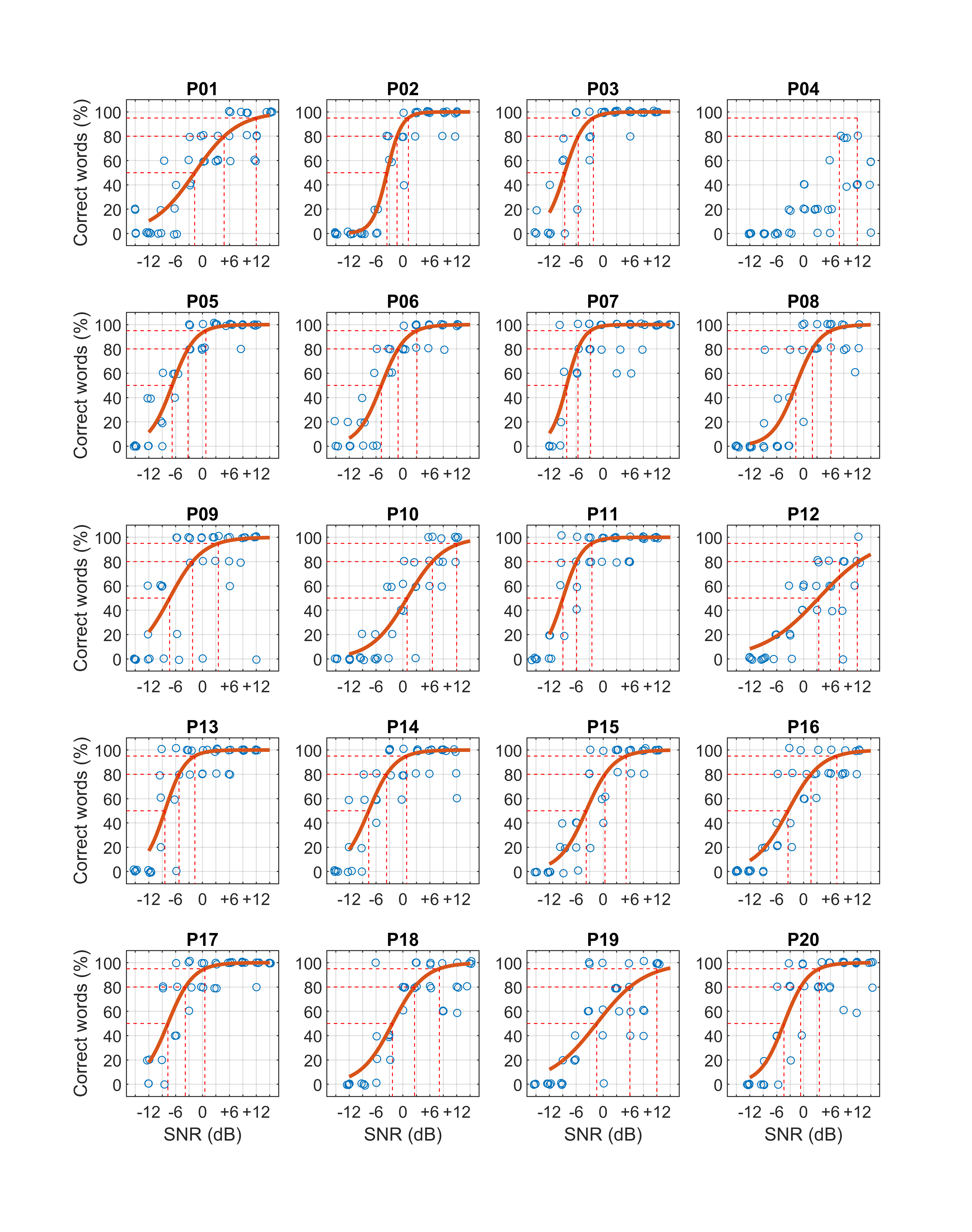

Supplement: Supplementary file 1 — Supplementary Information. [file 41598_2025_95045_MOESM1_ESM.zip › Appendix_B/FigureA2/FigureA2b.png]

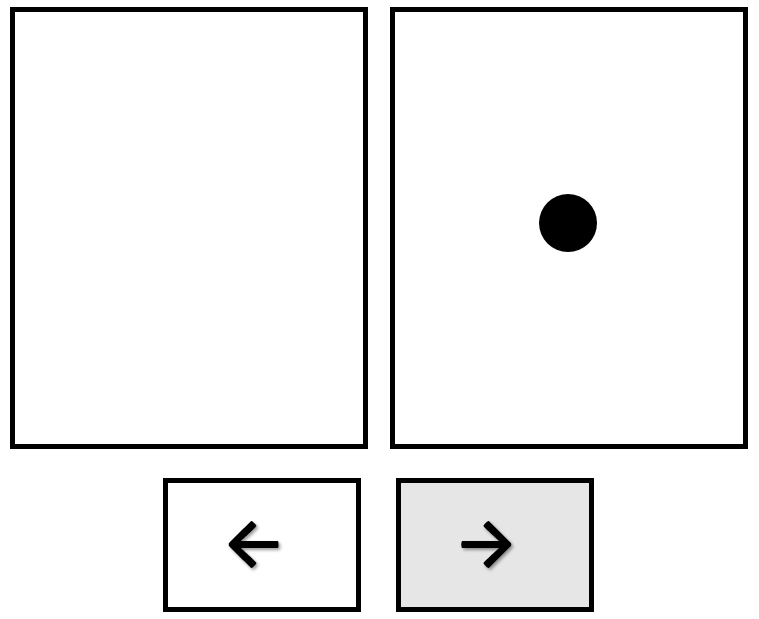

Supplement: Supplementary file 1 — Supplementary Information. [file 41598_2025_95045_MOESM1_ESM.zip › Appendix_B/FigureA3/FigureA3a.png]

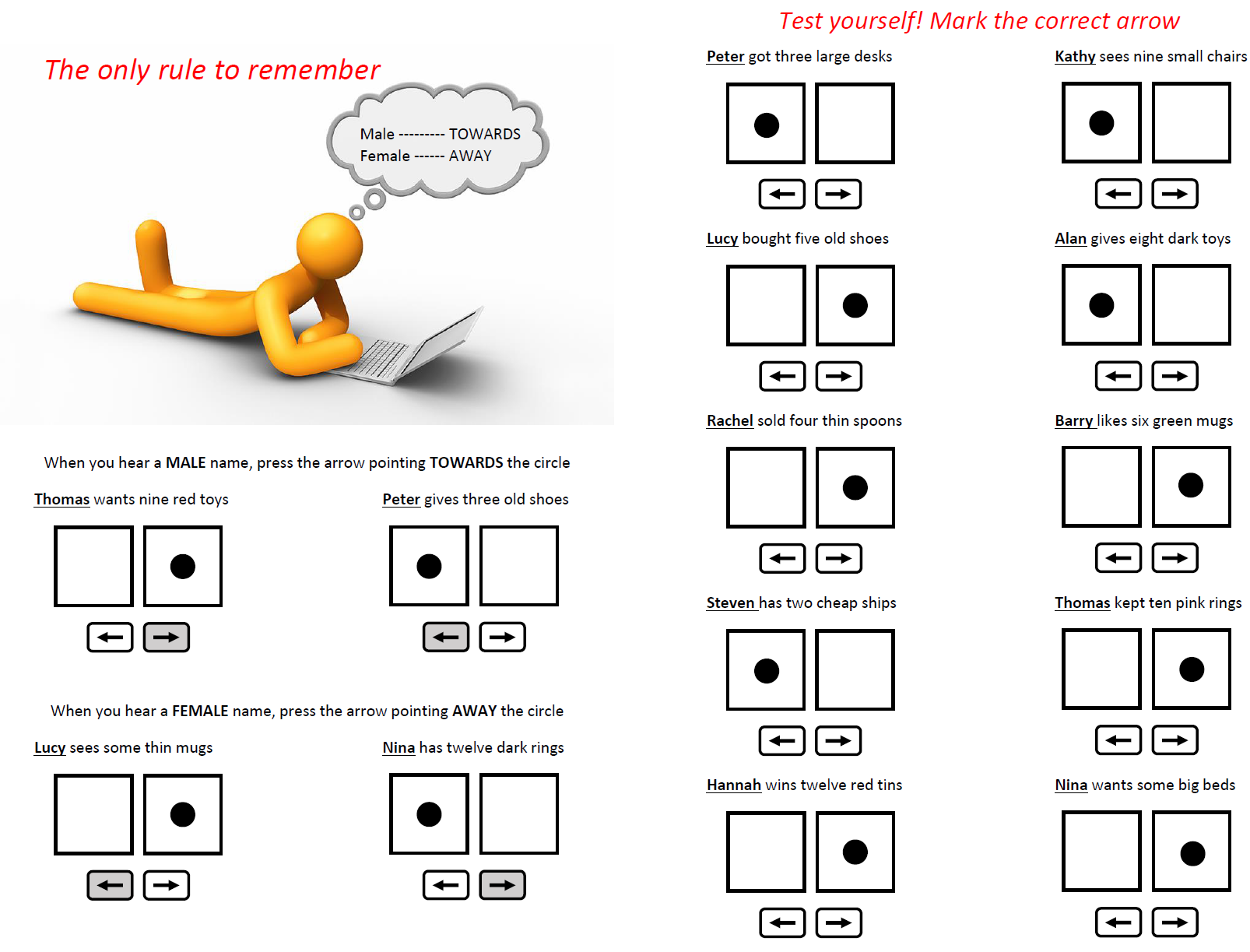

Supplement: Supplementary file 1 — Supplementary Information. [file 41598_2025_95045_MOESM1_ESM.zip › Appendix_B/FigureA3/FigureA3b.png]

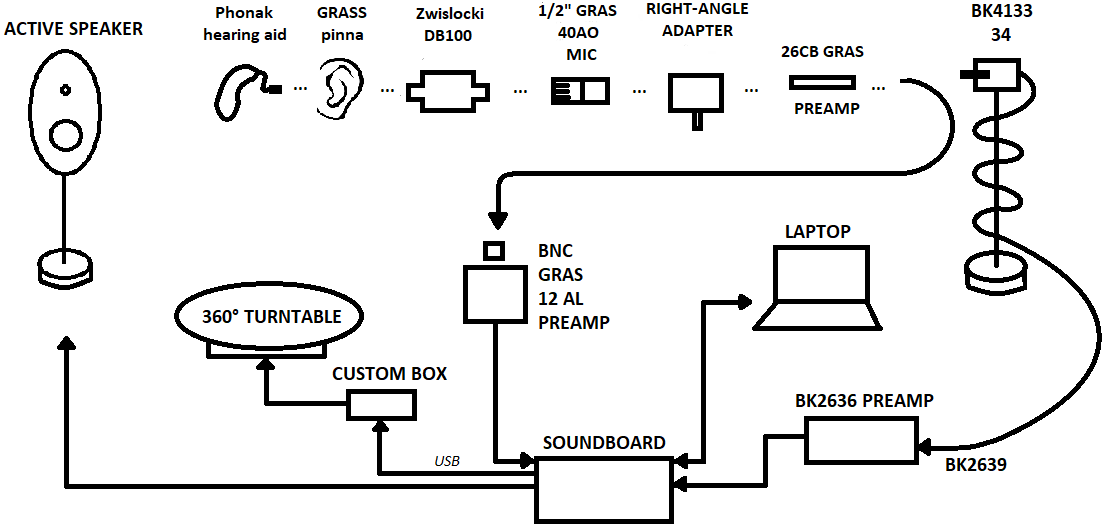

Supplement: Supplementary file 1 — Supplementary Information. [file 41598_2025_95045_MOESM1_ESM.zip › Appendix_B/FigureA4/FigureA4a.png]

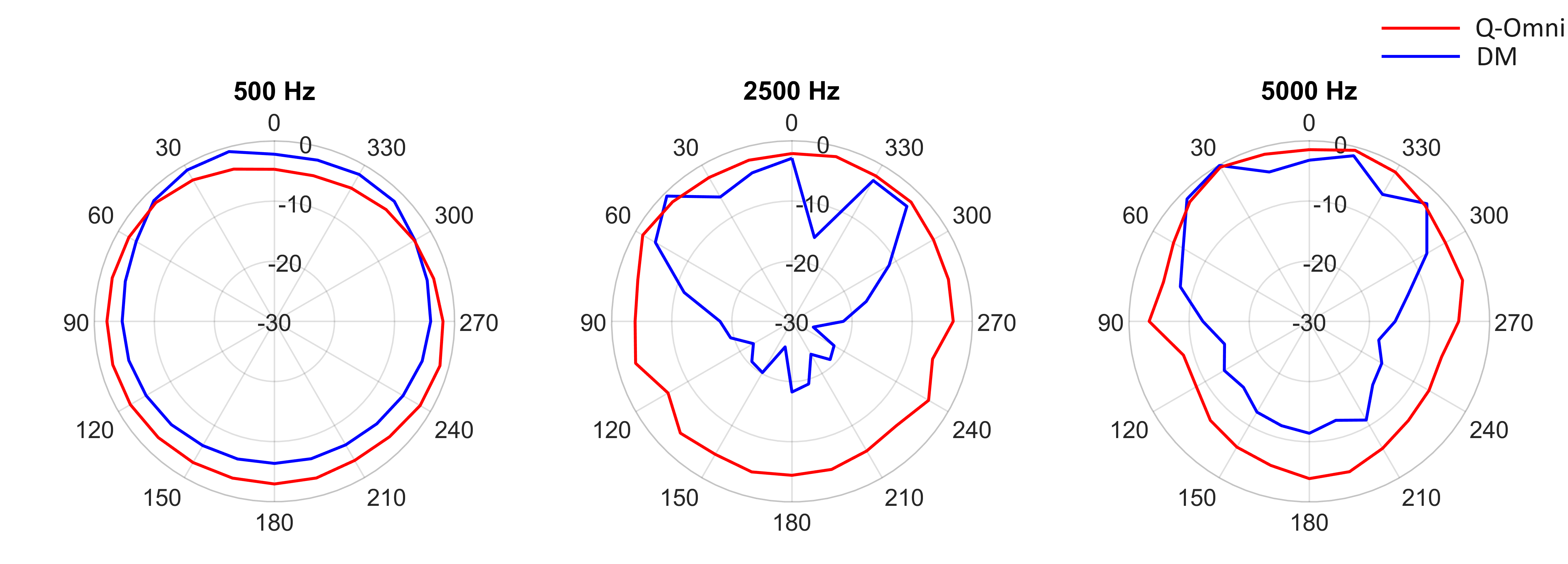

Supplement: Supplementary file 1 — Supplementary Information. [file 41598_2025_95045_MOESM1_ESM.zip › Appendix_B/FigureA4/FigureA4b.png]

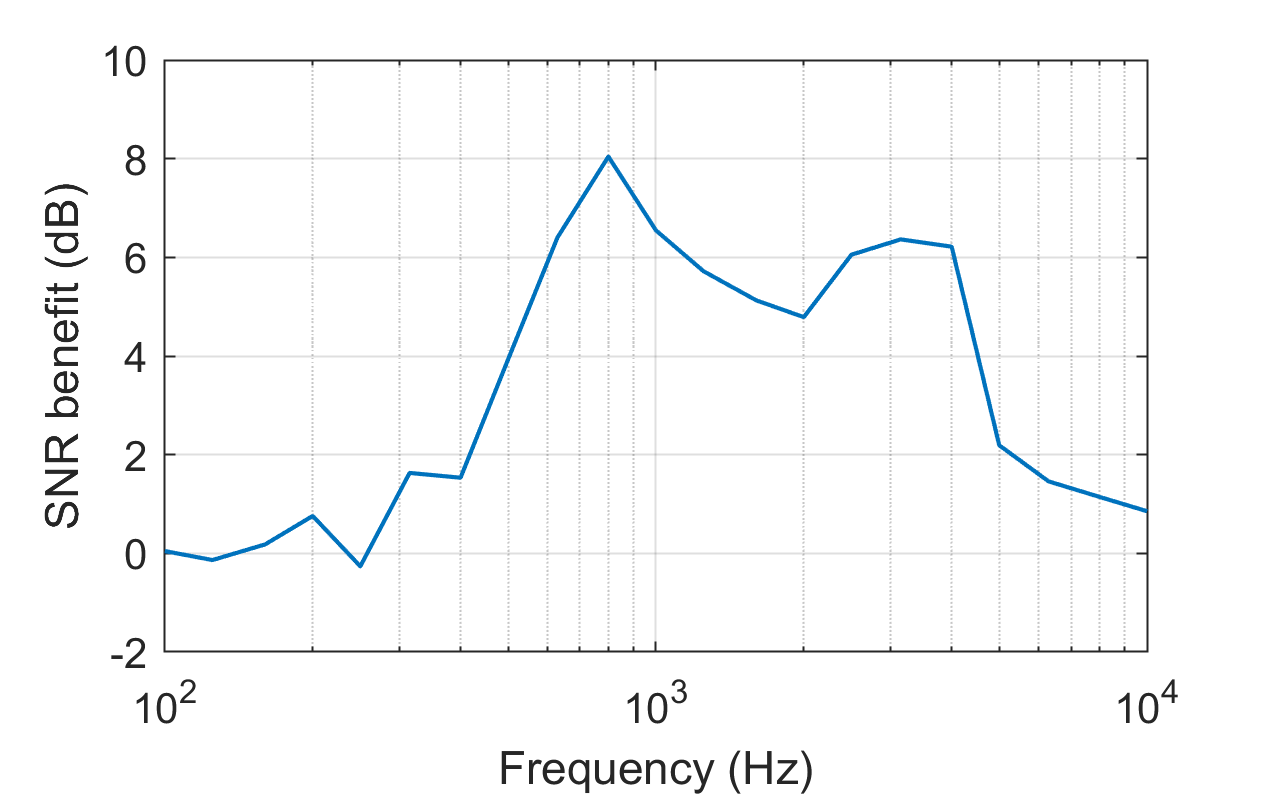

Supplement: Supplementary file 1 — Supplementary Information. [file 41598_2025_95045_MOESM1_ESM.zip › Appendix_B/FigureA4/FigureA4c.png]

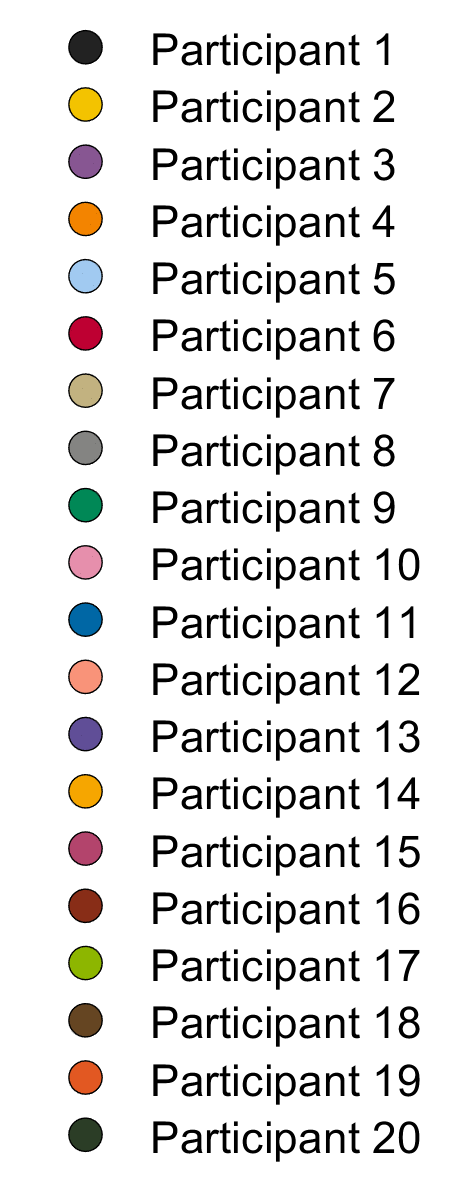

Supplement: Supplementary file 1 — Supplementary Information. [file 41598_2025_95045_MOESM1_ESM.zip › Appendix_B/FigureA7/Figure_A7.png]

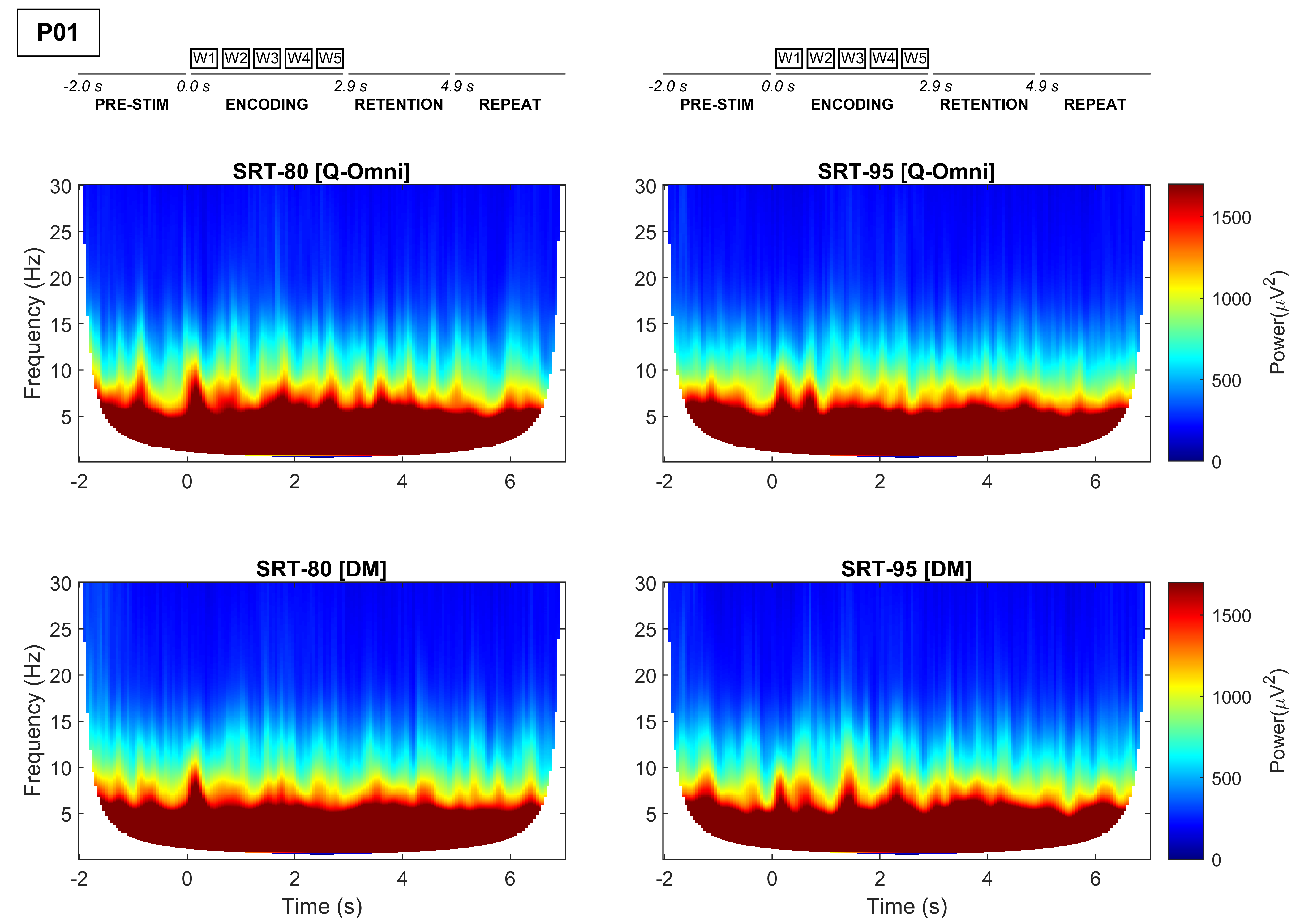

Supplement: Supplementary file 1 — Supplementary Information. [file 41598_2025_95045_MOESM1_ESM.zip › Appendix_B/FigureA8/Fig_P01.png]

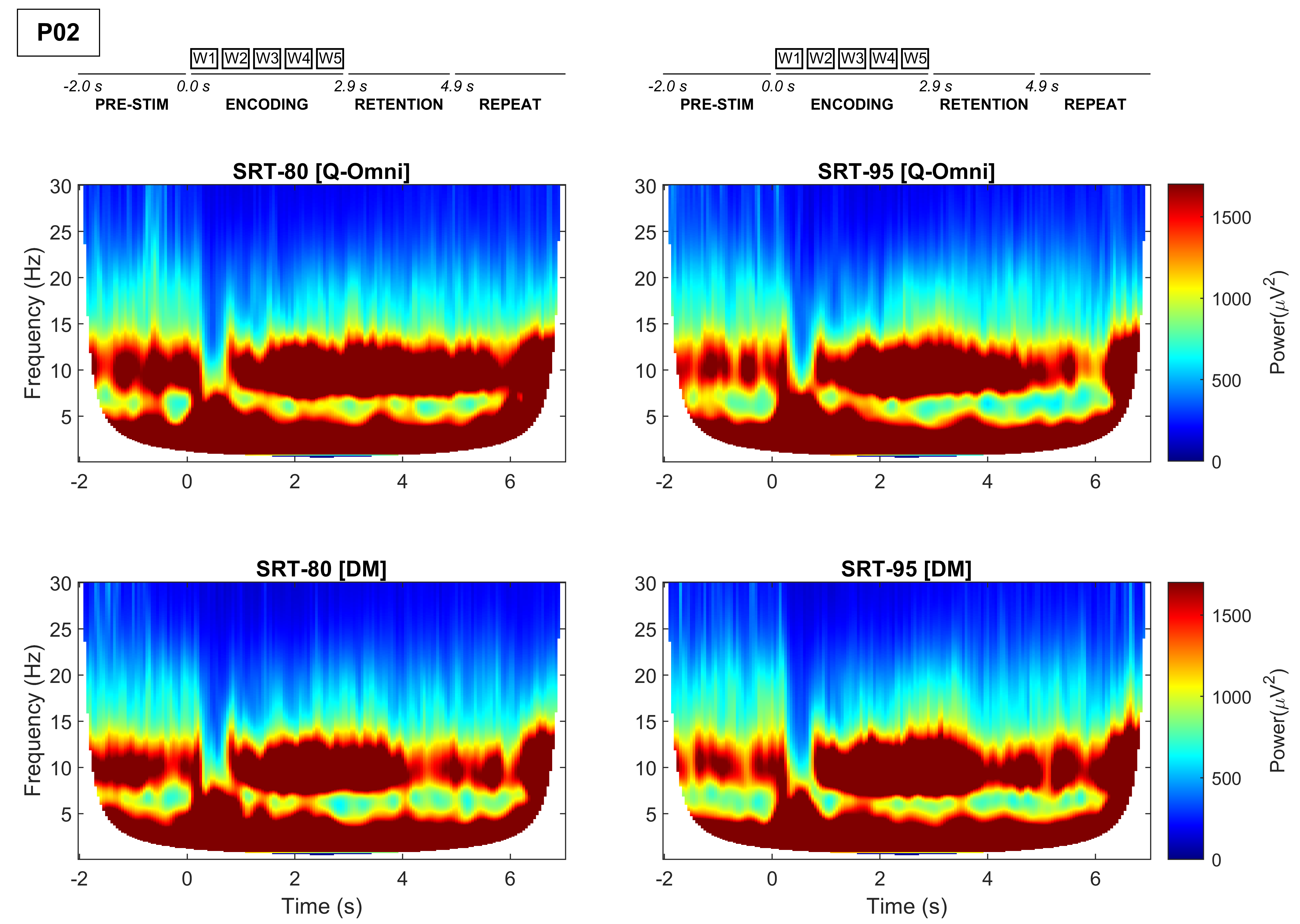

Supplement: Supplementary file 1 — Supplementary Information. [file 41598_2025_95045_MOESM1_ESM.zip › Appendix_B/FigureA8/Fig_P02.png]

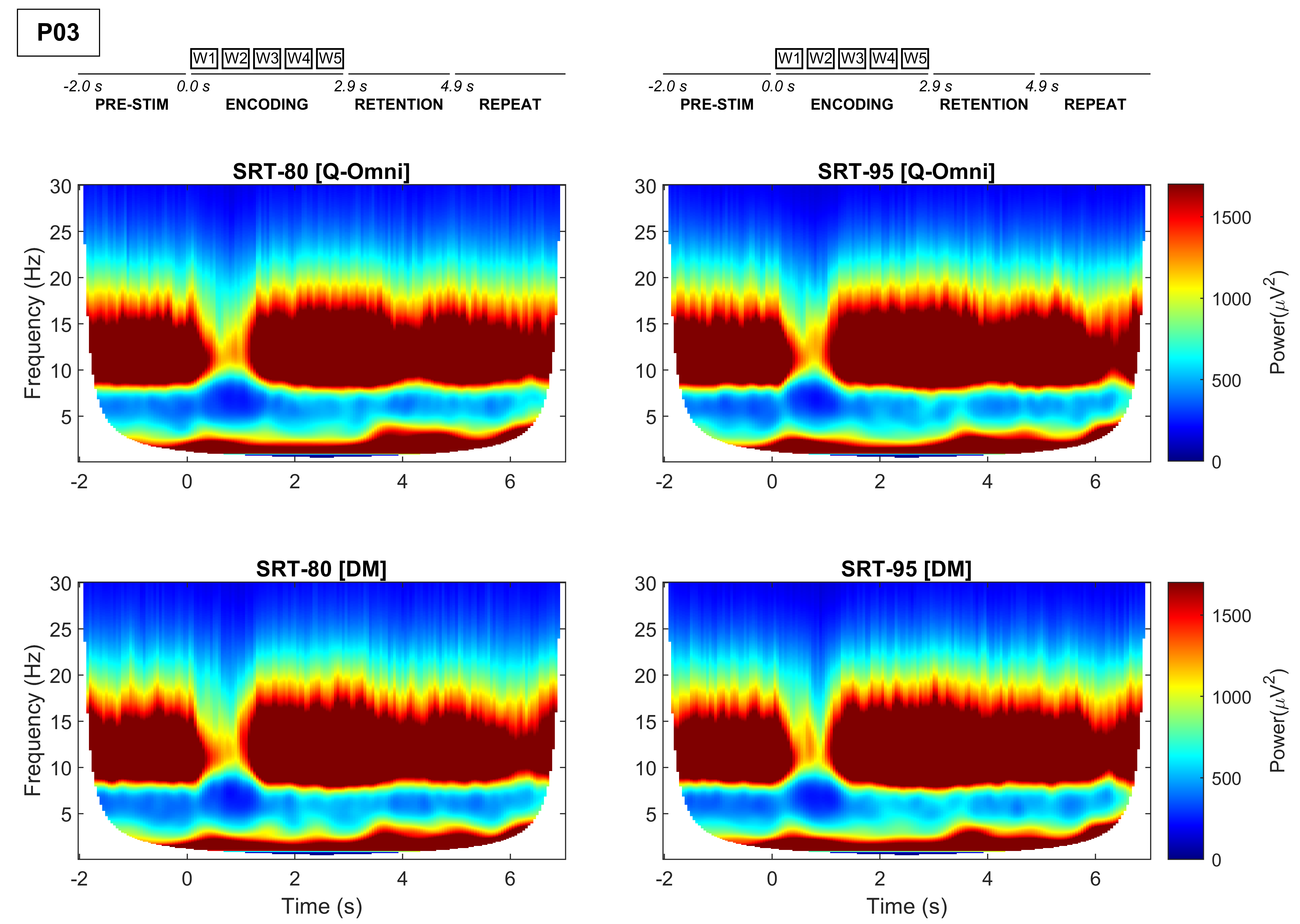

Supplement: Supplementary file 1 — Supplementary Information. [file 41598_2025_95045_MOESM1_ESM.zip › Appendix_B/FigureA8/Fig_P03.png]

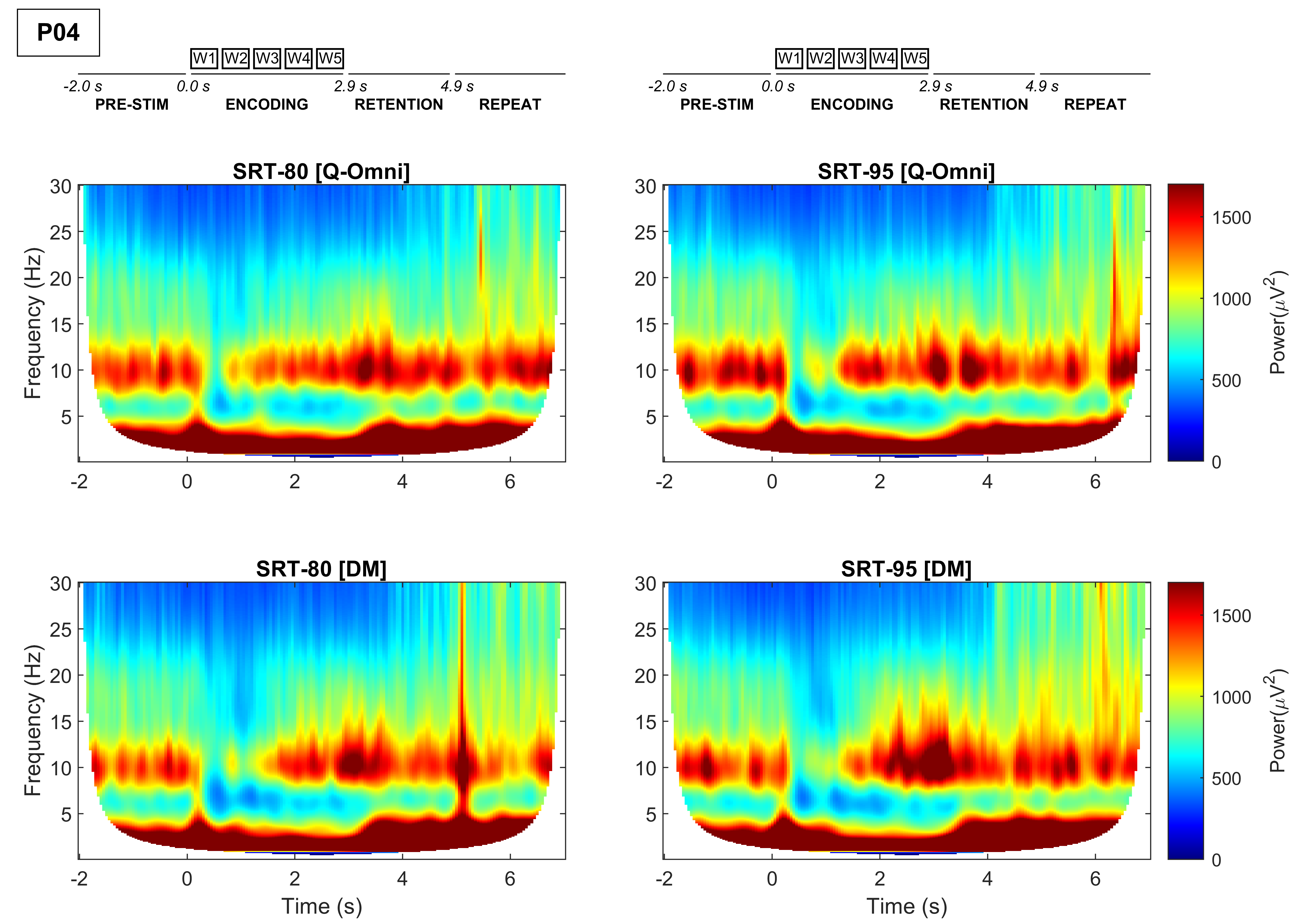

Supplement: Supplementary file 1 — Supplementary Information. [file 41598_2025_95045_MOESM1_ESM.zip › Appendix_B/FigureA8/Fig_P04.png]

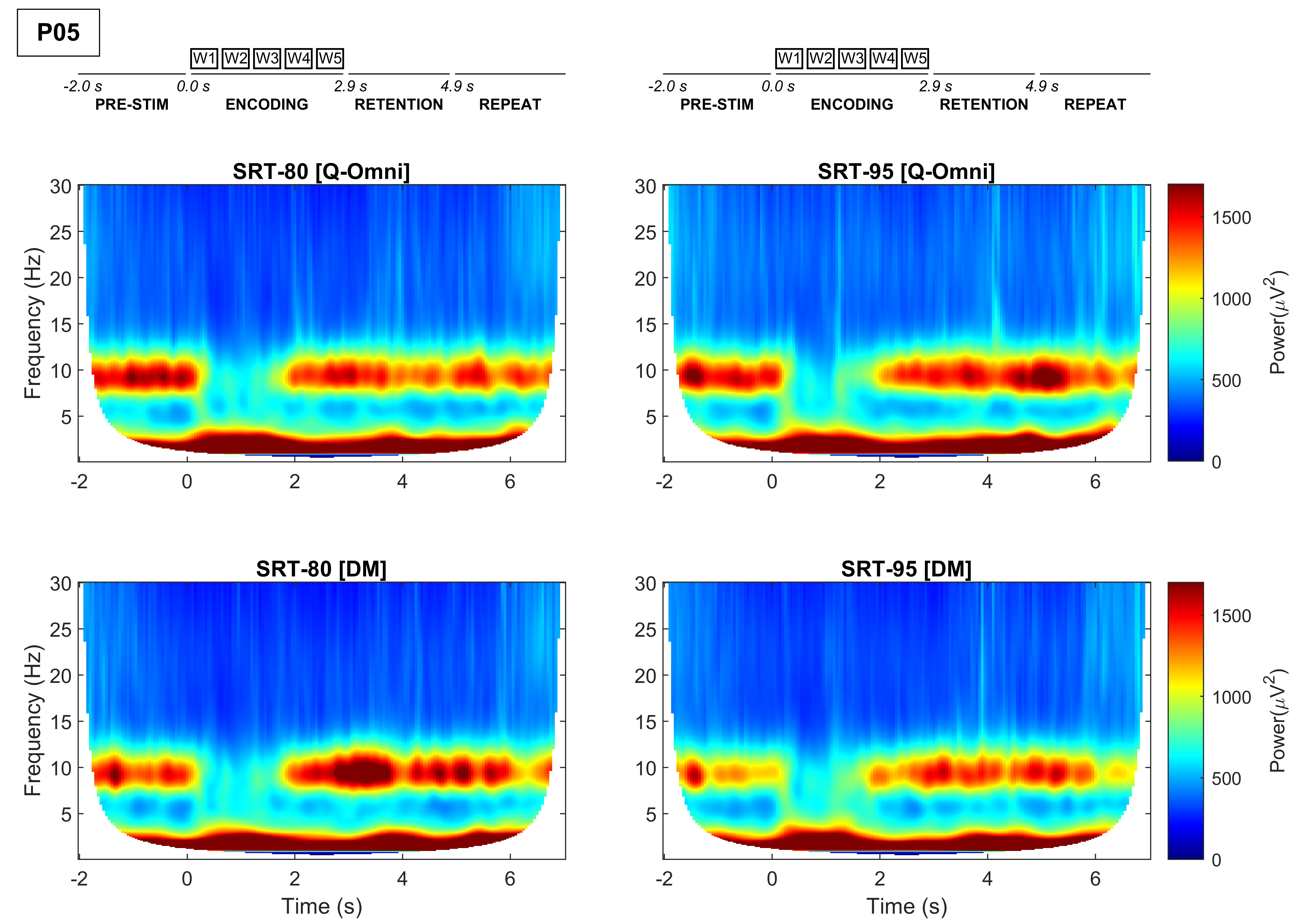

Supplement: Supplementary file 1 — Supplementary Information. [file 41598_2025_95045_MOESM1_ESM.zip › Appendix_B/FigureA8/Fig_P05.png]

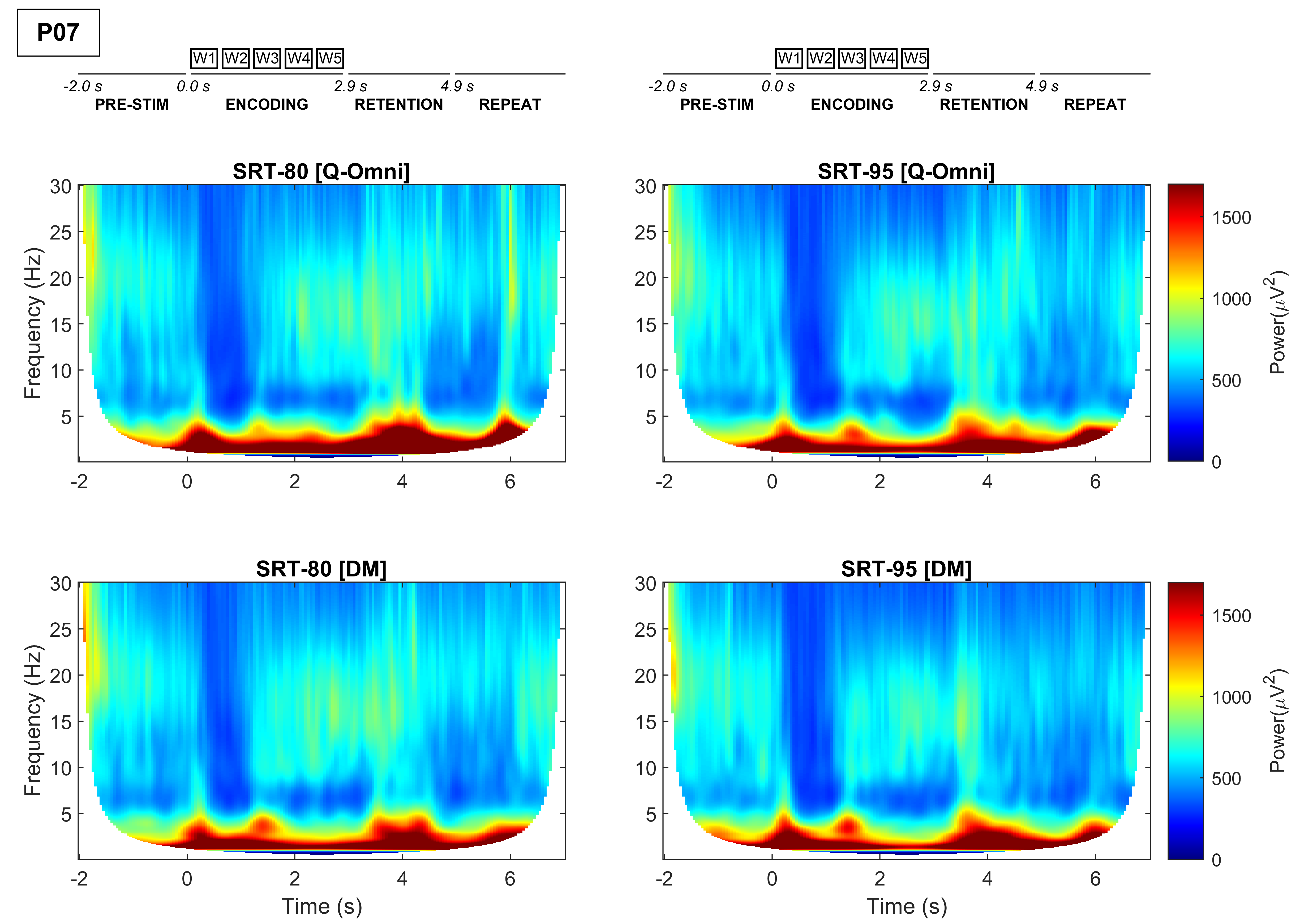

Supplement: Supplementary file 1 — Supplementary Information. [file 41598_2025_95045_MOESM1_ESM.zip › Appendix_B/FigureA8/Fig_P07.png]

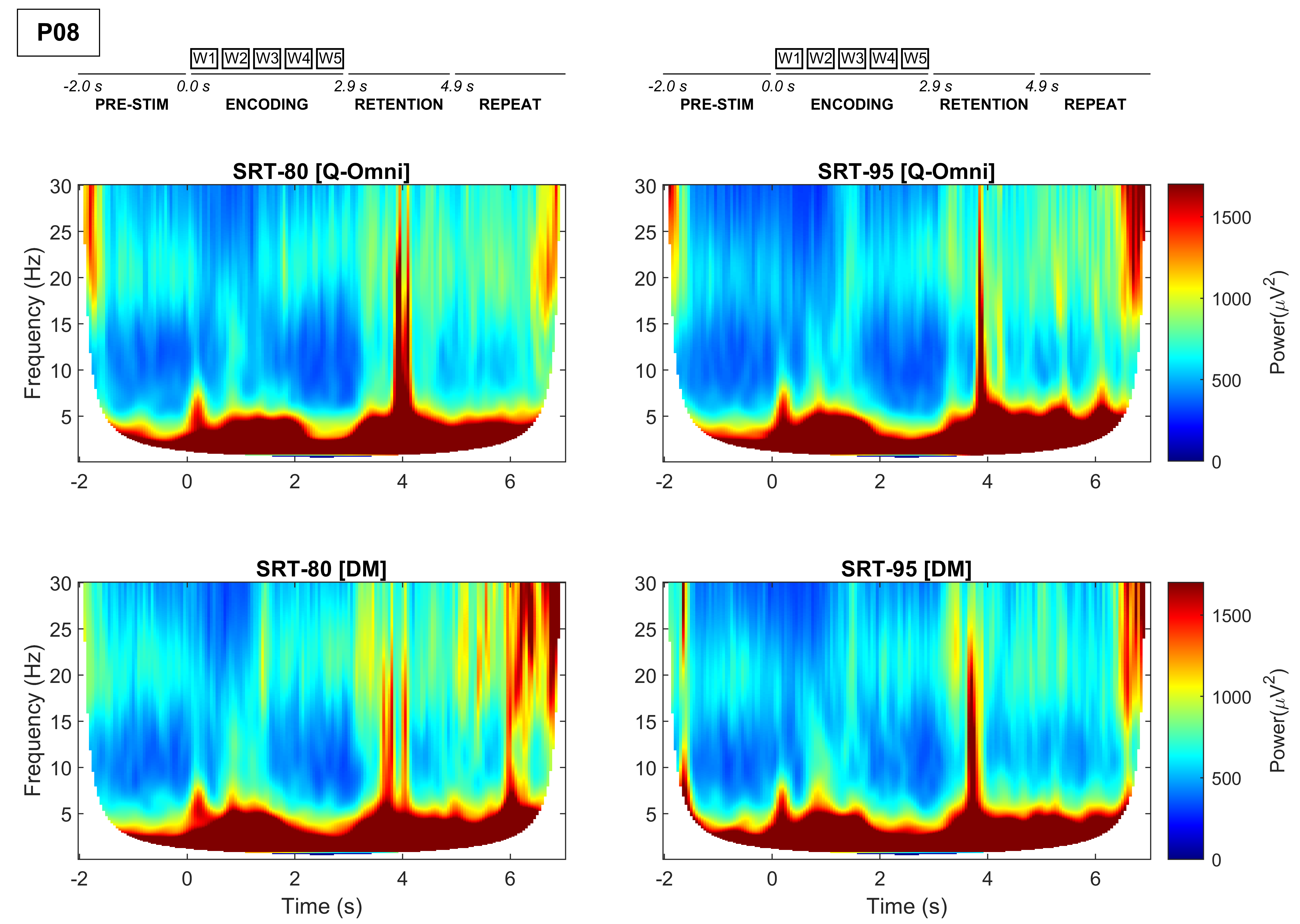

Supplement: Supplementary file 1 — Supplementary Information. [file 41598_2025_95045_MOESM1_ESM.zip › Appendix_B/FigureA8/Fig_P08.png]

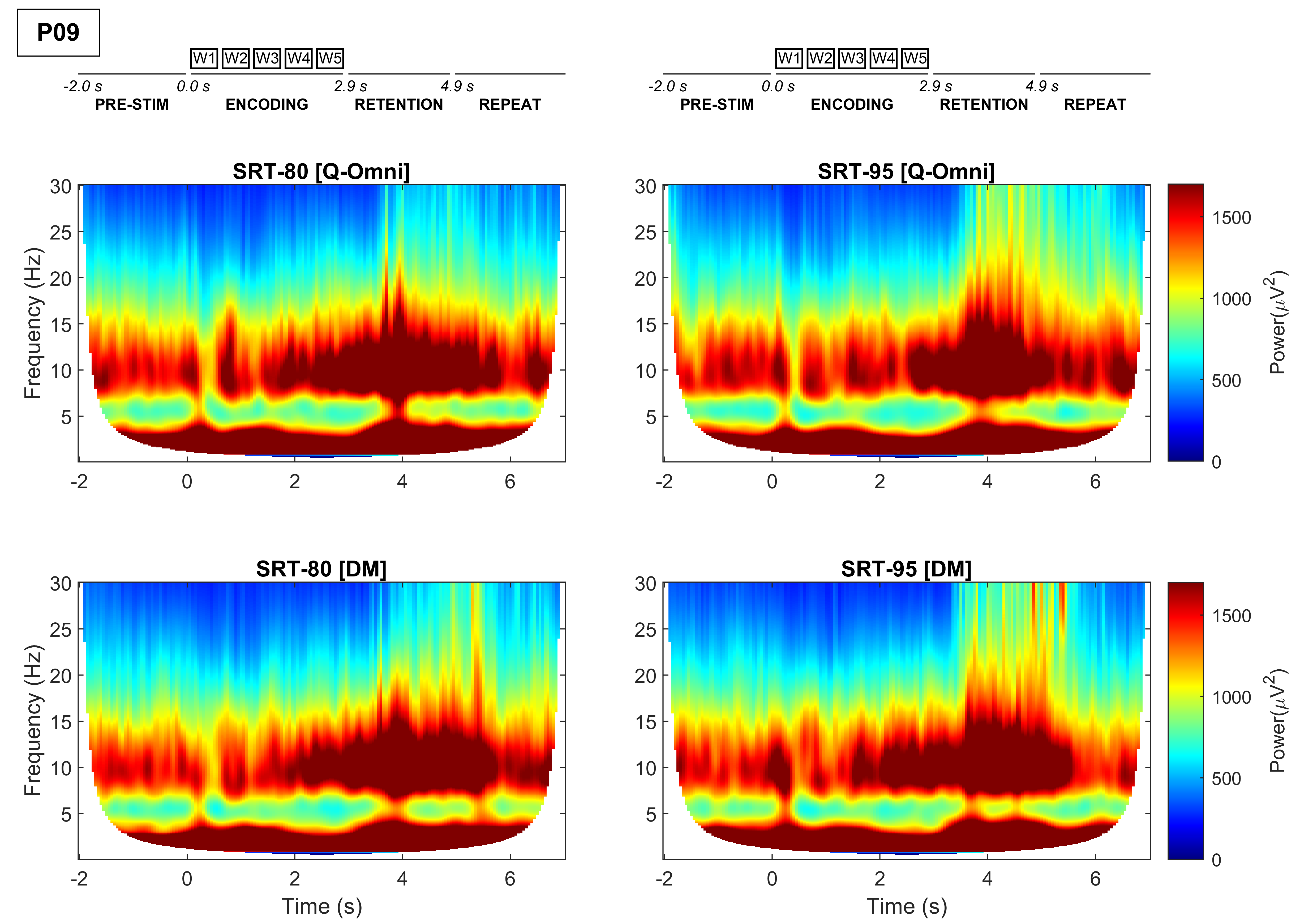

Supplement: Supplementary file 1 — Supplementary Information. [file 41598_2025_95045_MOESM1_ESM.zip › Appendix_B/FigureA8/Fig_P09.png]

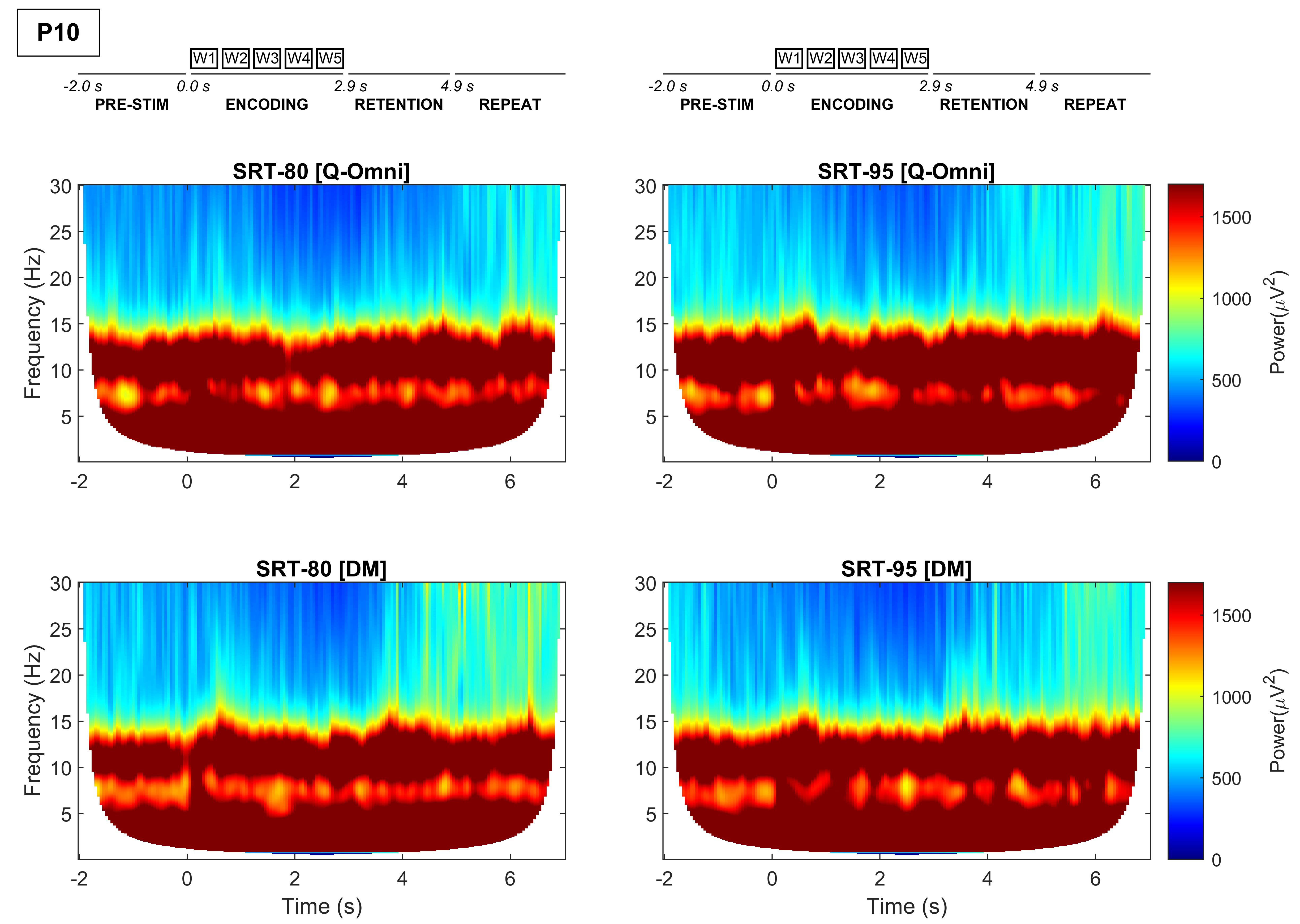

Supplement: Supplementary file 1 — Supplementary Information. [file 41598_2025_95045_MOESM1_ESM.zip › Appendix_B/FigureA8/Fig_P10.png]

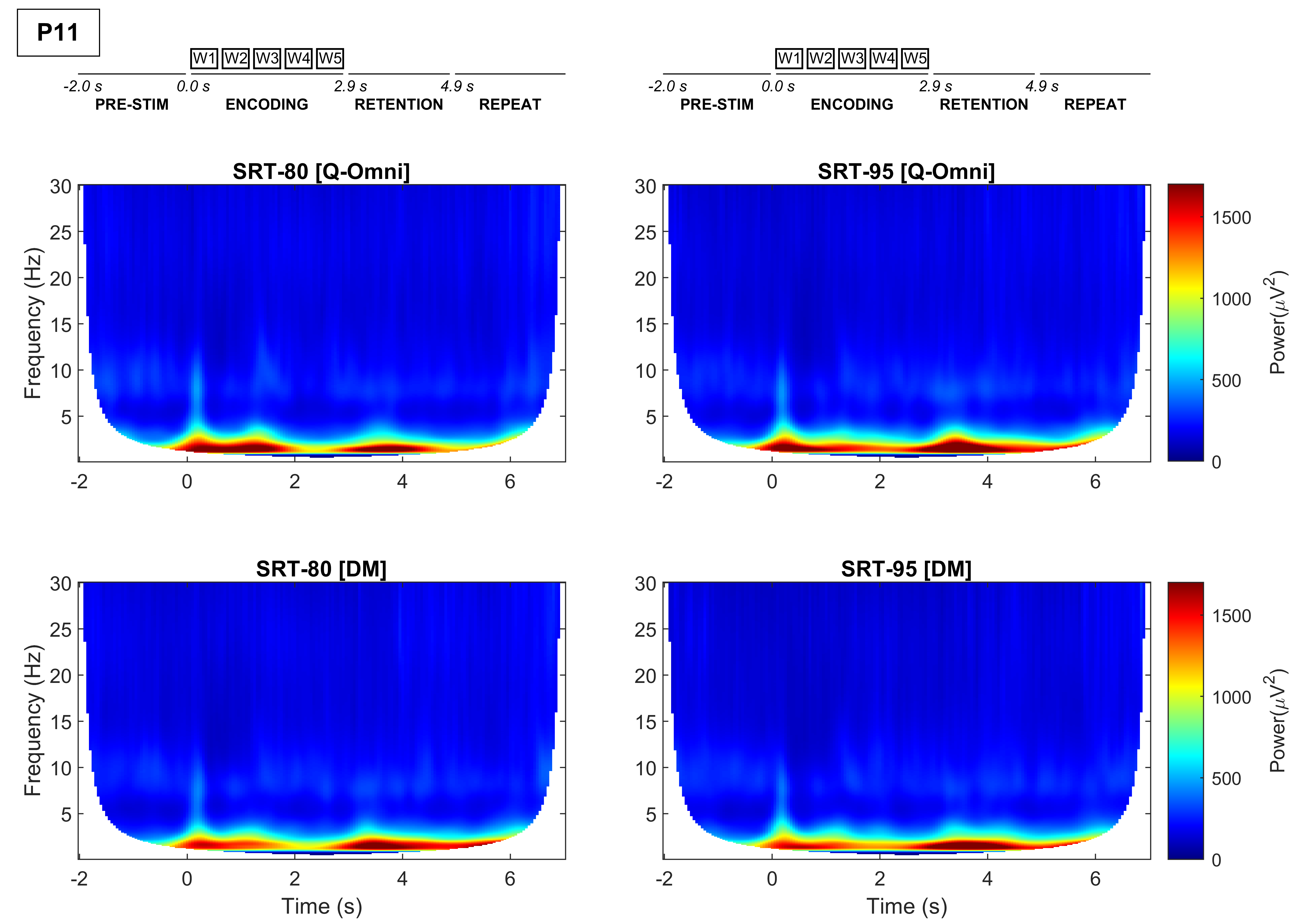

Supplement: Supplementary file 1 — Supplementary Information. [file 41598_2025_95045_MOESM1_ESM.zip › Appendix_B/FigureA8/Fig_P11.png]

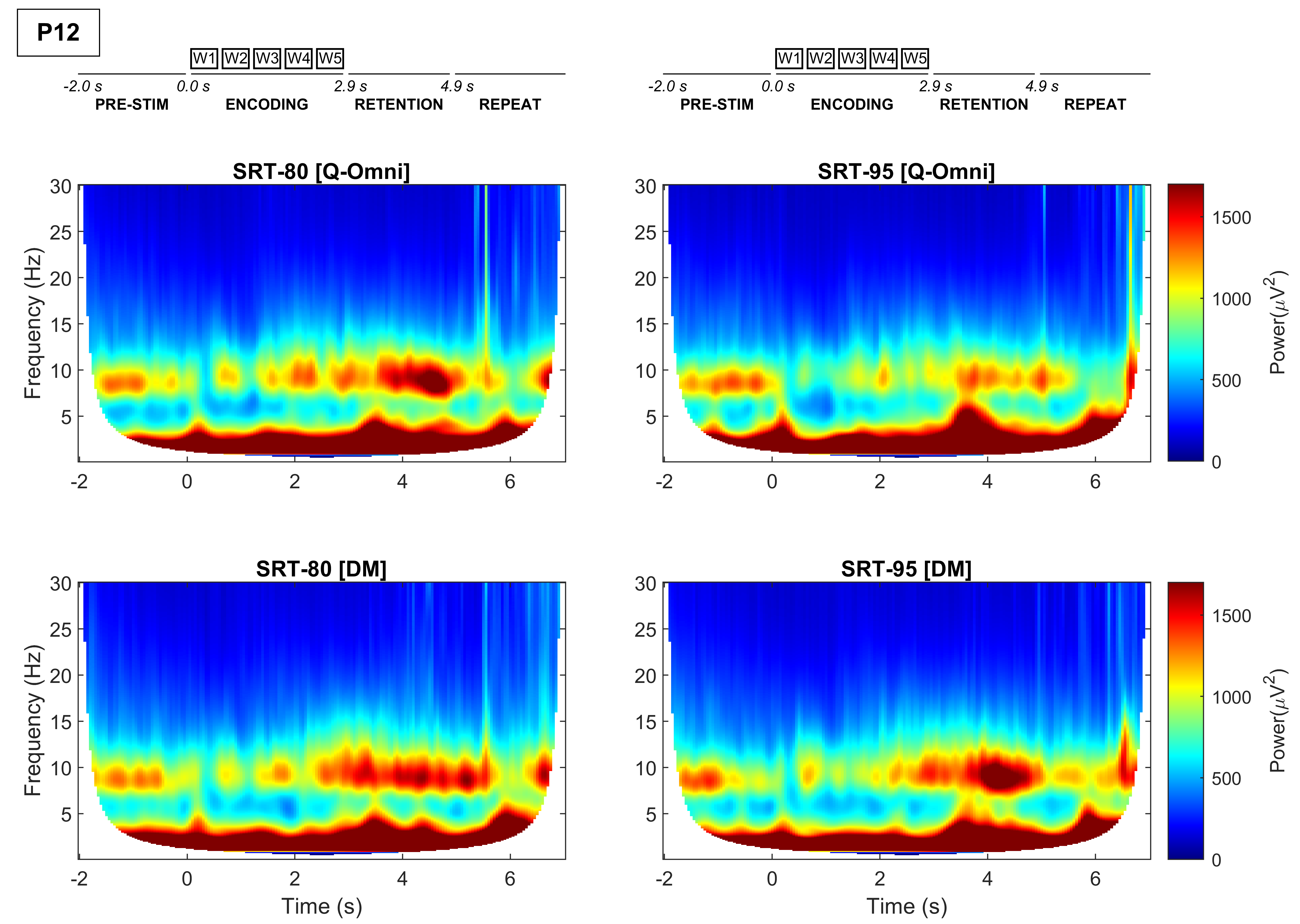

Supplement: Supplementary file 1 — Supplementary Information. [file 41598_2025_95045_MOESM1_ESM.zip › Appendix_B/FigureA8/Fig_P12.png]

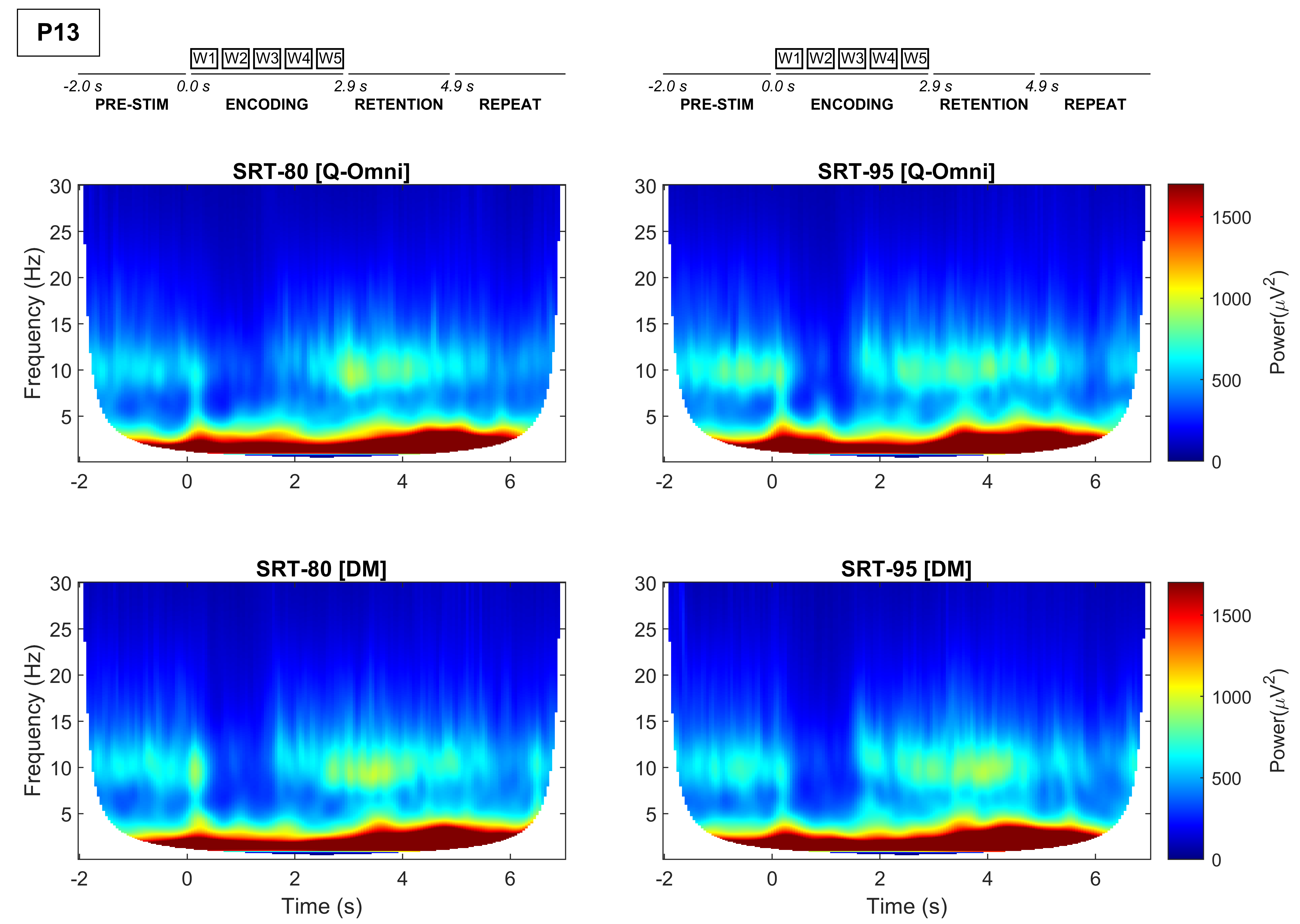

Supplement: Supplementary file 1 — Supplementary Information. [file 41598_2025_95045_MOESM1_ESM.zip › Appendix_B/FigureA8/Fig_P13.png]

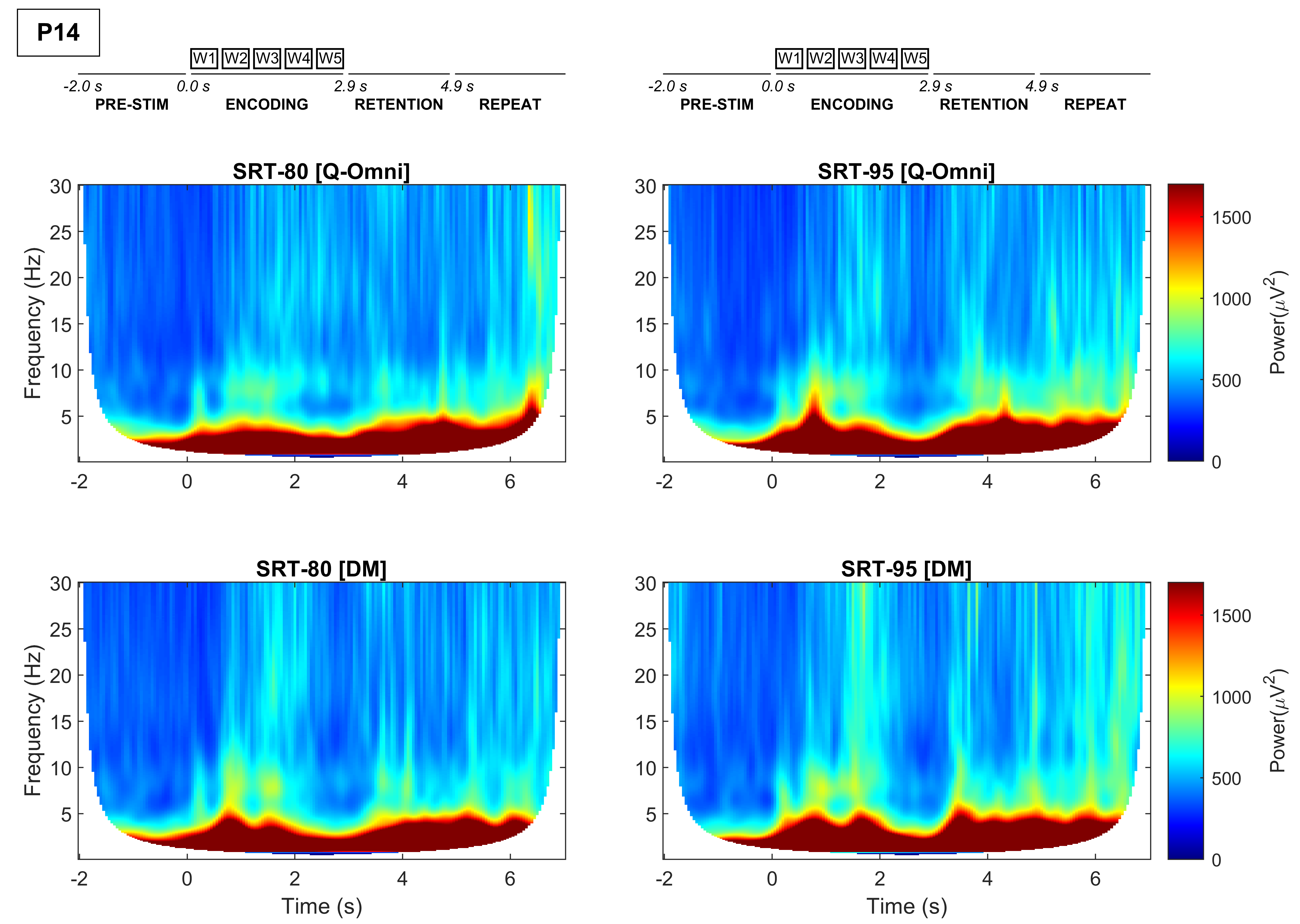

Supplement: Supplementary file 1 — Supplementary Information. [file 41598_2025_95045_MOESM1_ESM.zip › Appendix_B/FigureA8/Fig_P14.png]

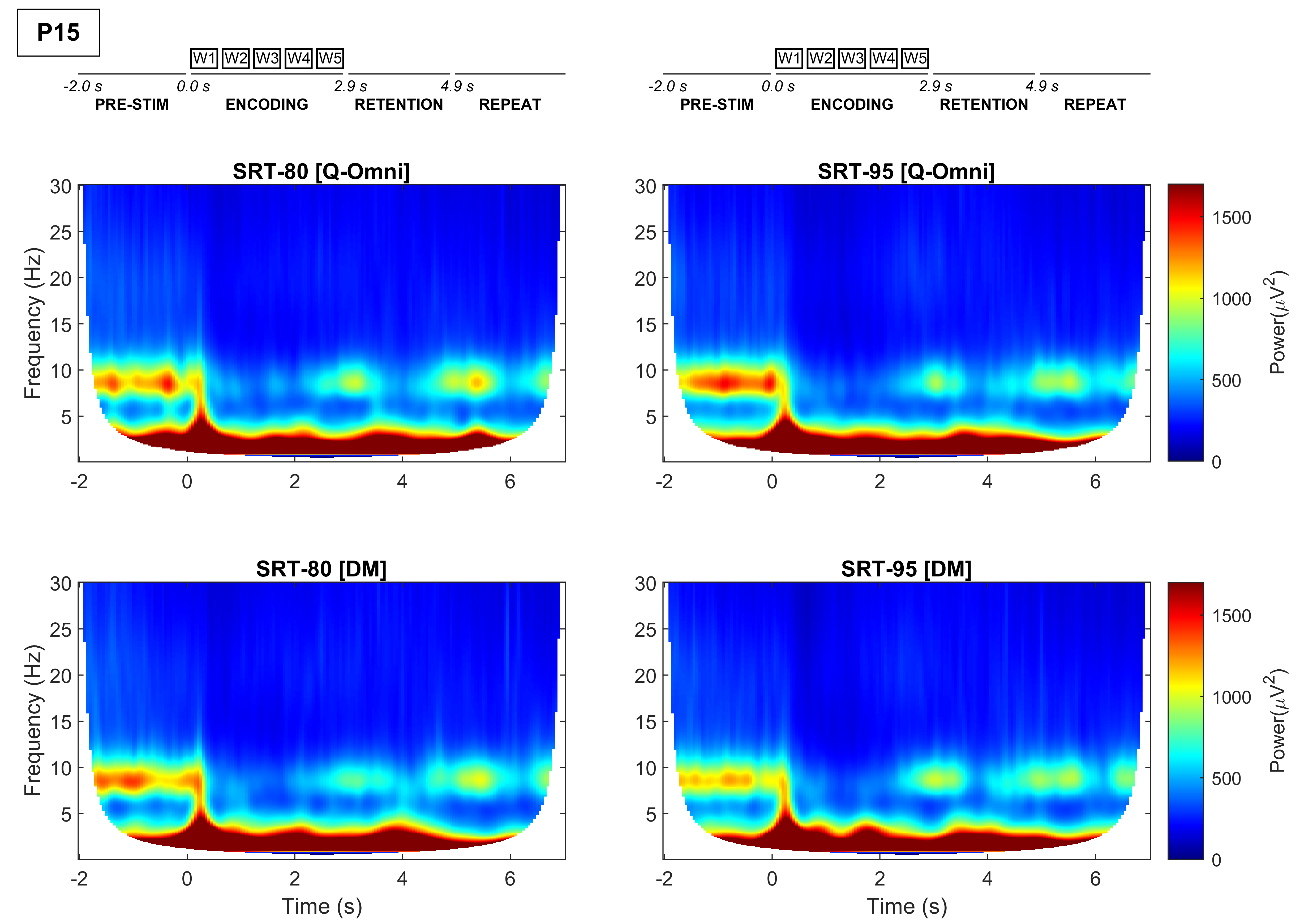

Supplement: Supplementary file 1 — Supplementary Information. [file 41598_2025_95045_MOESM1_ESM.zip › Appendix_B/FigureA8/Fig_P15.png]

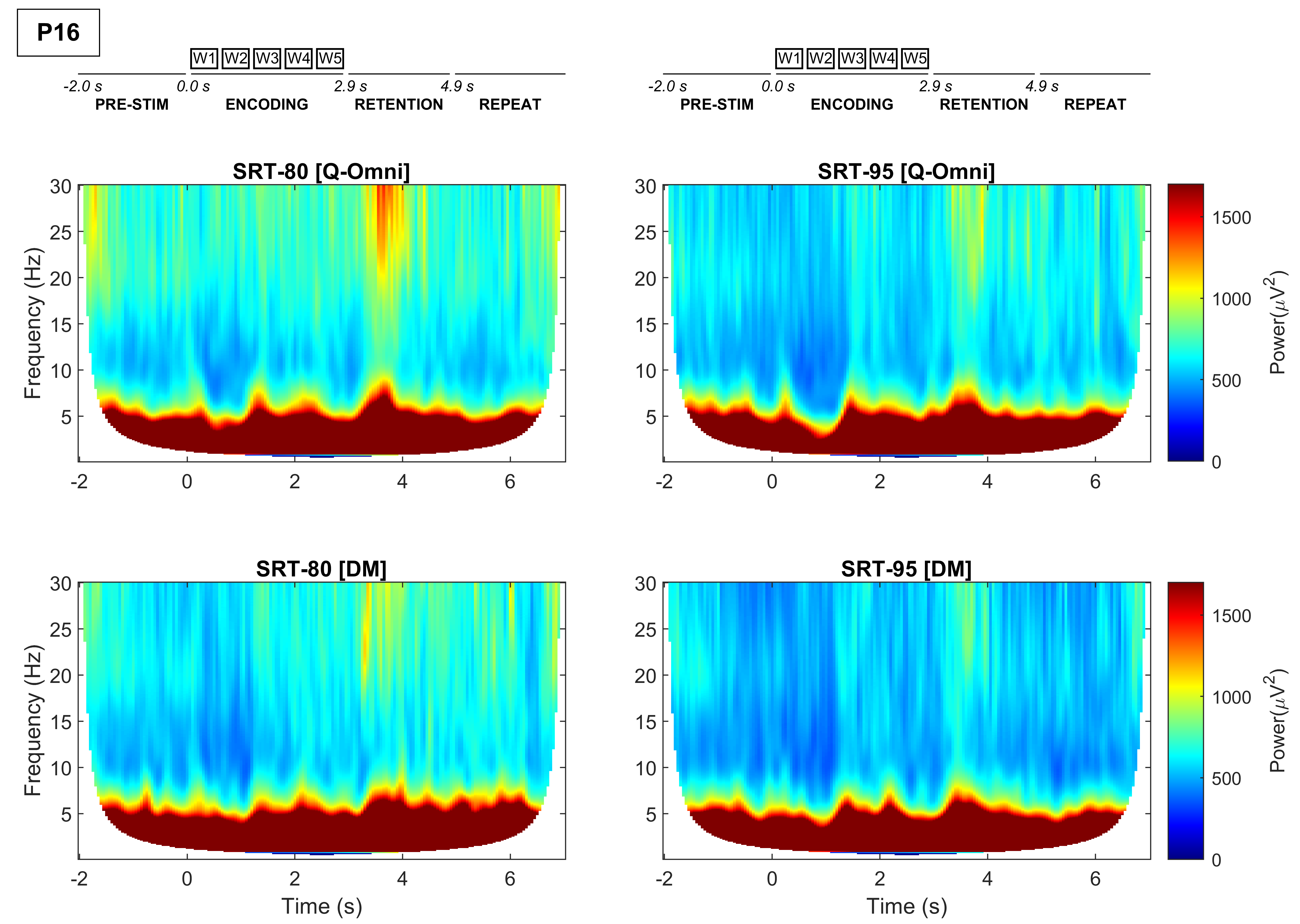

Supplement: Supplementary file 1 — Supplementary Information. [file 41598_2025_95045_MOESM1_ESM.zip › Appendix_B/FigureA8/Fig_P16.png]

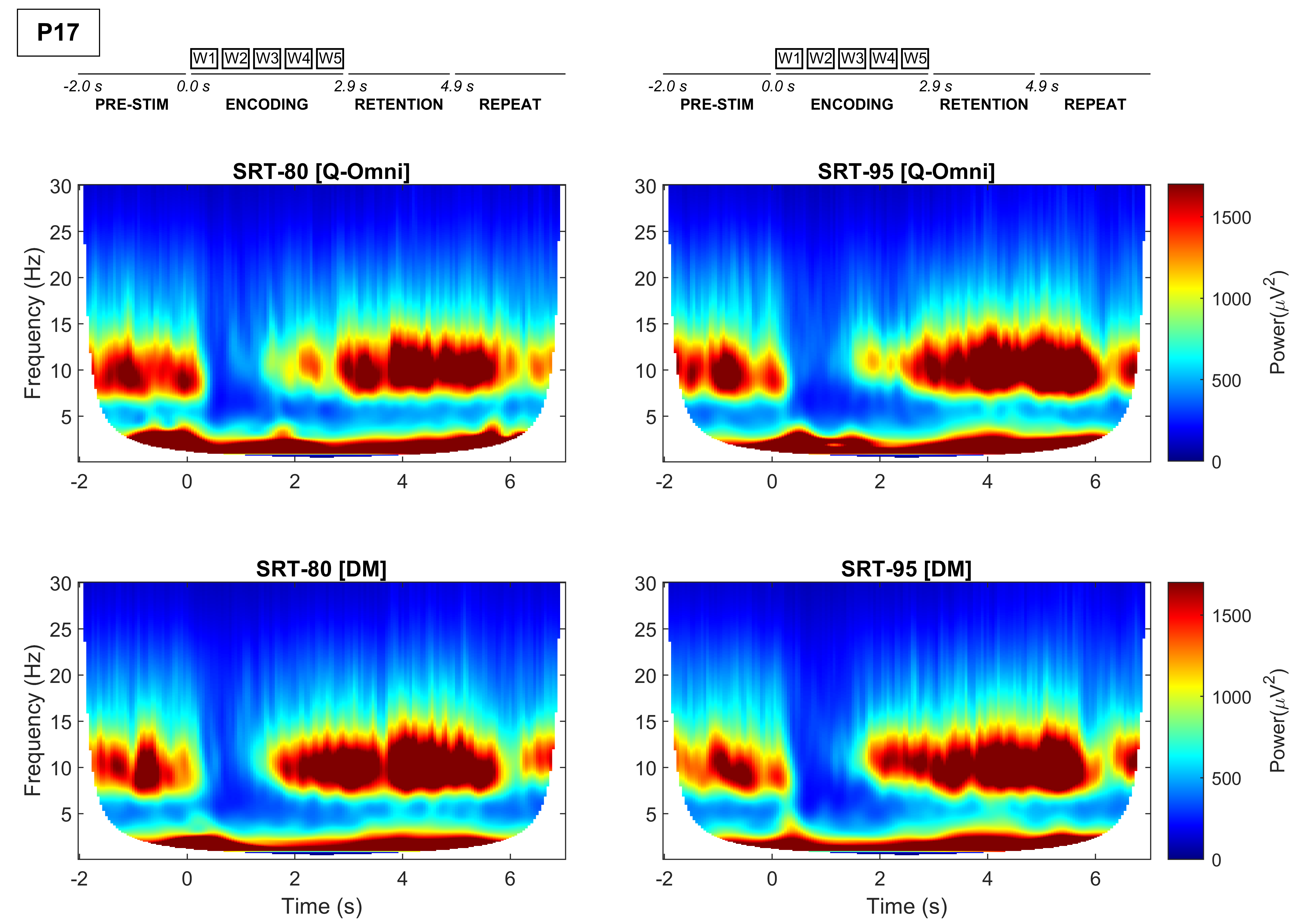

Supplement: Supplementary file 1 — Supplementary Information. [file 41598_2025_95045_MOESM1_ESM.zip › Appendix_B/FigureA8/Fig_P17.png]

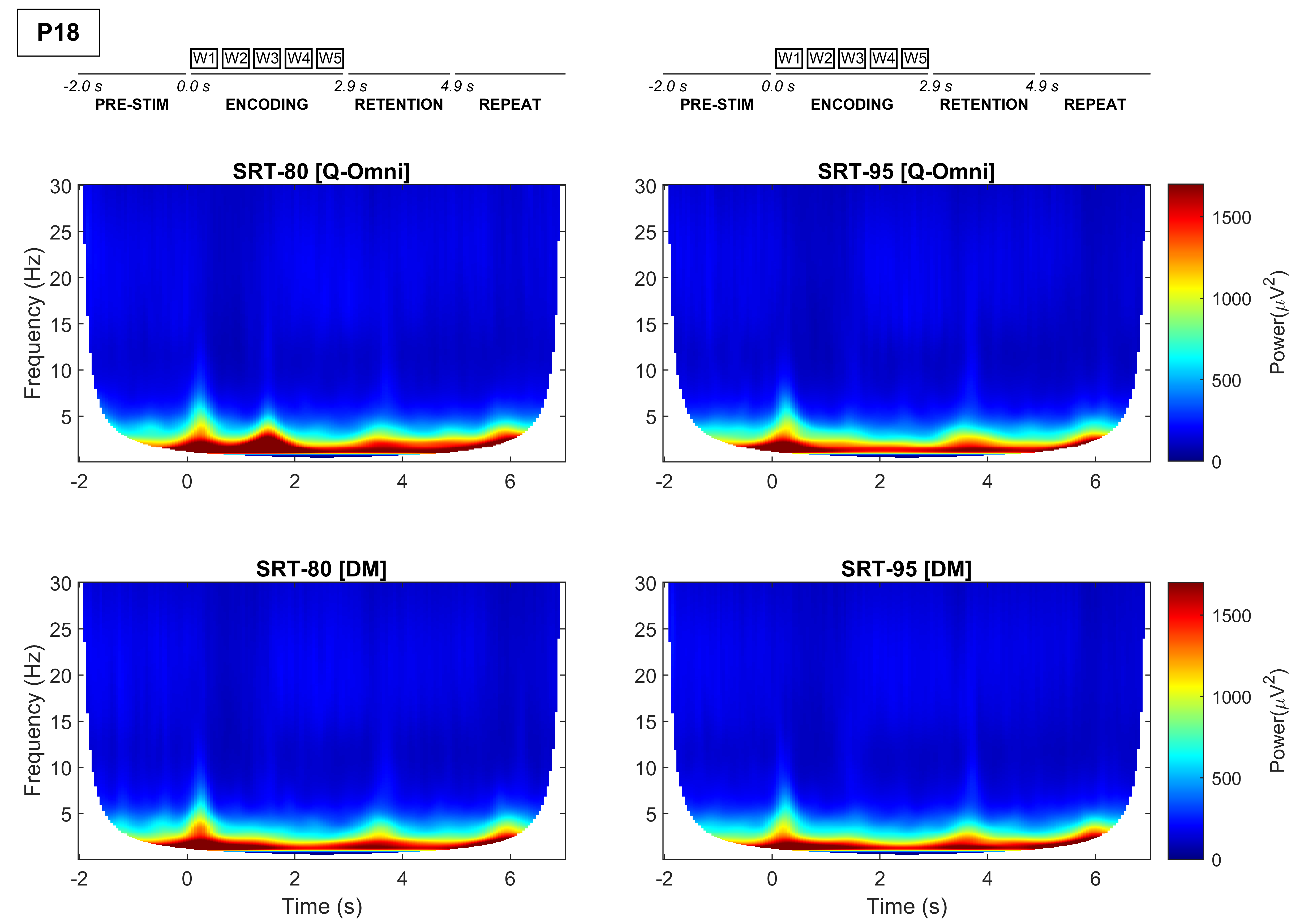

Supplement: Supplementary file 1 — Supplementary Information. [file 41598_2025_95045_MOESM1_ESM.zip › Appendix_B/FigureA8/Fig_P18.png]

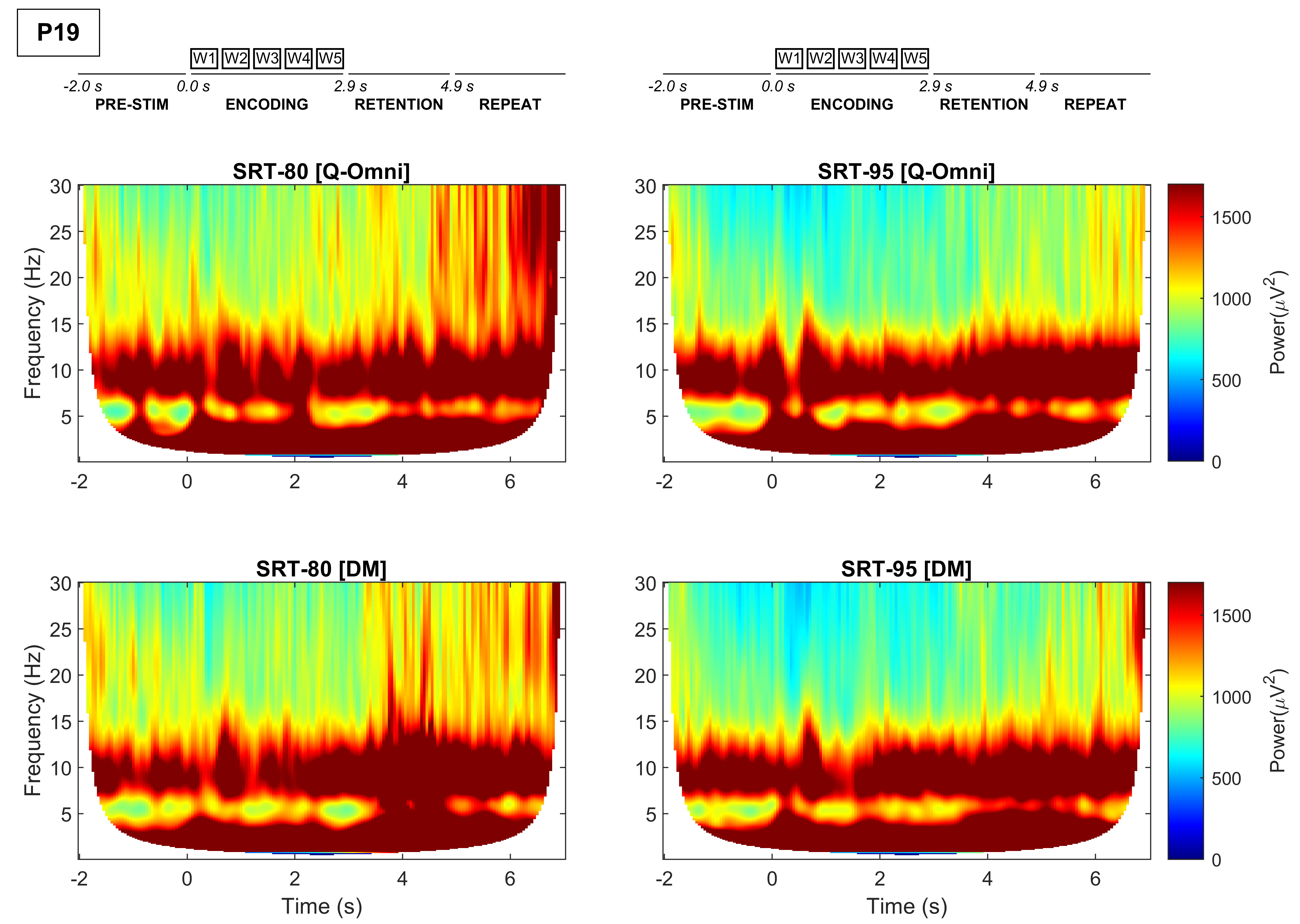

Supplement: Supplementary file 1 — Supplementary Information. [file 41598_2025_95045_MOESM1_ESM.zip › Appendix_B/FigureA8/Fig_P19.png]

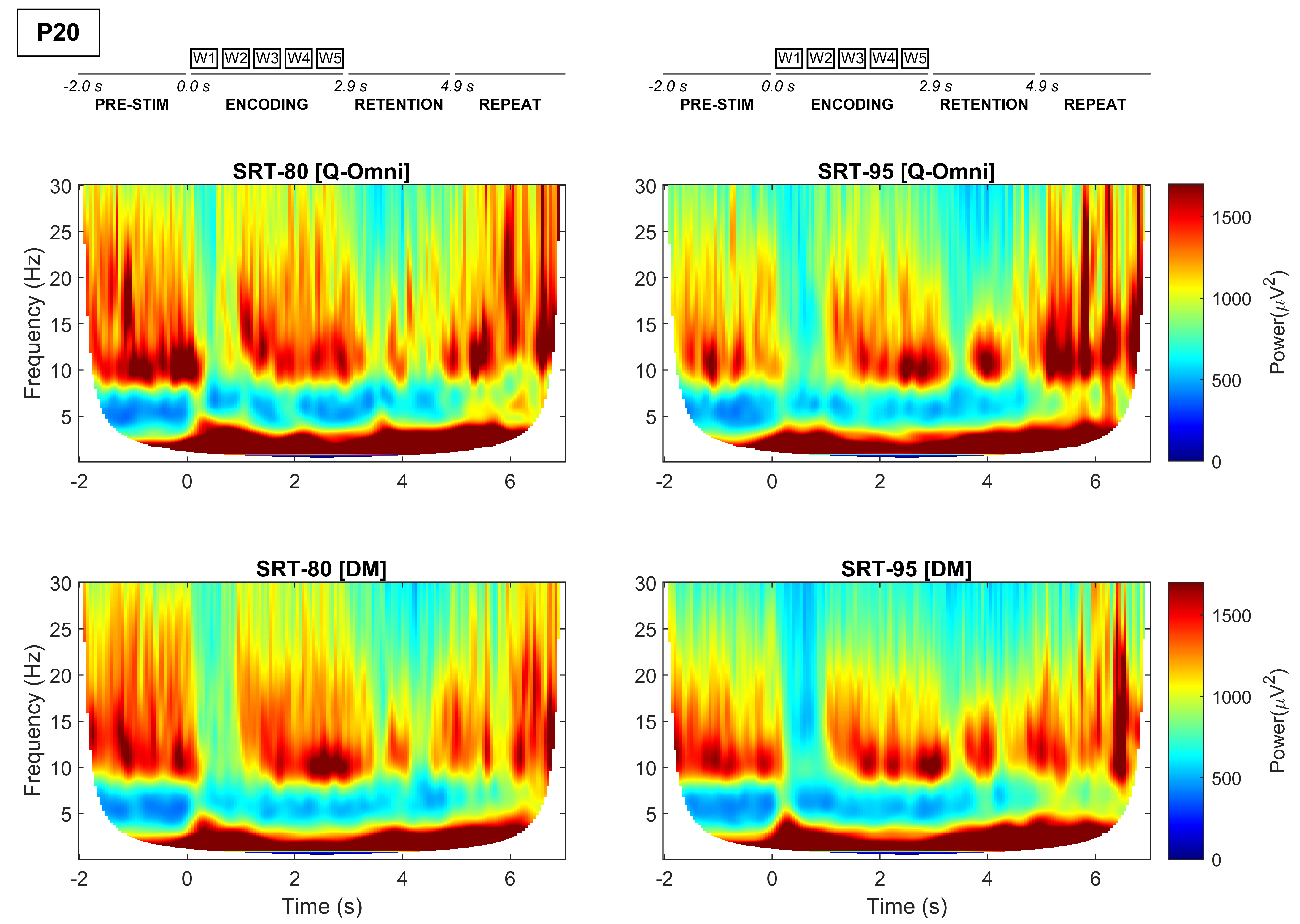

Supplement: Supplementary file 1 — Supplementary Information. [file 41598_2025_95045_MOESM1_ESM.zip › Appendix_B/FigureA8/Fig_P20.png]
